# An Icelandic pangenome reference

**DOI:** 10.1038/s41586-026-10924-7

**Published:** 2026-08-19

**Authors:** Guillaume Holley, Hannes P. Eggertsson, Snaedis Kristmundsdottir, Doruk Beyter, Astros Th Skuladottir, Kristjan H. S. Moore, Pall I. Olason, Arnaldur Gylfason, Olafur T. Magnusson, Asmundur Oddsson, Hreinn Stefansson, Agnar Helgason, Gisli Masson, Patrick Sulem, Daniel F. Gudbjartsson, Kari Stefansson, Bjarni V. Halldorsson

**Affiliations:** 1 deCODE genetics/Amgen, Reykjavik, Iceland; 2 Faculty of Medicine, School of Health Sciences, University of Iceland, Reykjavik, Iceland; 3 Department of Anthropology, University of Iceland, Reykjavik, Iceland; 4 School of Engineering and Natural Sciences, University of Iceland, Reykjavik, Iceland; 5 School of Technology, Reykjavik University, Reykjavik, Iceland

**Keywords:** Population genetics, Software, Genome-wide association studies, Parkinson's disease, Genome informatics

## Abstract

Reference bias is an issue that affects most genomic studies analysing short reads mapped to a reference genome^1,2^. It can be mitigated by mapping to multiple haplotypes represented in a pangenome^3–5^. Here we introduce two new methods to address reference bias: Emblask for pangenome construction and Weaver for mapping to pangenomes at scale. Emblask is a hybrid long- and short-read haplotype-resolved dual assembly pipeline for parent–offspring trio data. Using Emblask, we assembled 698 Icelandic haplotypes and added them to the Human Pangenome Reference Consortium (HPRC) pangenome^4^ to construct an Icelandic pangenome reference (HPRC-ICE) including 51.41 million small variants. We mapped the short reads of 57,630 Icelanders to HPRC-ICE with Weaver and called 98.96 million variants, representing a 6.17% increase over mapping to a linear reference. We uncovered new variants in low-mappability regions, including a pathogenic single nucleotide polymorphism (SNP) in *GBA1* that associates with early onset Parkinson’s disease and a missense SNP in *CBS* that is pathogenic for homocystinuria. We replicated the *GBA1* association in the UK Biobank^6^ with a targeted remapping of 429,193 British and Irish participants.

## Abstract (web summary)

Newly developed methods enable construction of Icelandic haplotypes and mapping of population-scale short reads to a pangenome by reducing reference bias and improving discovery in low-mappability regions, revealing many novel variants, including pathogenic alleles.

## Main

Analyses of human genetic sequences^7–9^ are predominantly based on short reads mapped to a single reference genome^10,11^, resulting in an inaccurate or an incomplete mapping owing to gaps in the reference sequences and a strong bias towards the reference allele^1,2^. The current human reference genome^11^ (GRCh38) has been updated several times since its initial release with alternate contigs that represent chromosomal sequences diverging from its primary scaffolds. Although those additional sequences contribute to more diversity in GRCh38, they are often discarded from genomics analysis because of their similarity with primary scaffolds, which often leads to mapping ambiguity and in turn decreased variant calling sensitivity. Furthermore, GRCh38 is still an incomplete representation of the human genome, as its primary scaffolds contain about 151 Mb of gaps and 9 Mb of erroneously duplicated or collapsed regions^12^. The recent advance of long read sequencing^13^ has enabled the first gapless assembly of a human genome^14^, T2T-CHM13, comprising near error-free assemblies of the 22 autosomes and chromosome X, later complemented by a chromosome Y assembly^15^. Although T2T-CHM13 adds more than 182 Mb of sequence with respect to GRCh38, including 99 novel protein-coding genes, it does not capture any sequence differences between haplotypes or individuals.

Computational pangenomics is a field of study dedicated to the joint analysis and usage of genomic sequence collections over traditional linear references^3,16–22^. To this end, the HPRC has sequenced the genomes of 47 individuals from various ethnic groups and assembled them into contiguous high-quality haplotype-resolved assemblies^4^. The genetic variations of these individuals have been extracted from their assemblies and combined into a pangenome represented as a graph (Methods). Overall, the HPRC pangenome contributes an additional 90 Mb of non-reference sequences derived from structural variants and 119 Mb of euchromatic sequences with respect to GRCh38. Despite this, widespread adoption of pangenomes has been hindered due to their structural complexity, usage of new file formats and more computational requirements than required for linear reference mapping.

**Fig. 1:**
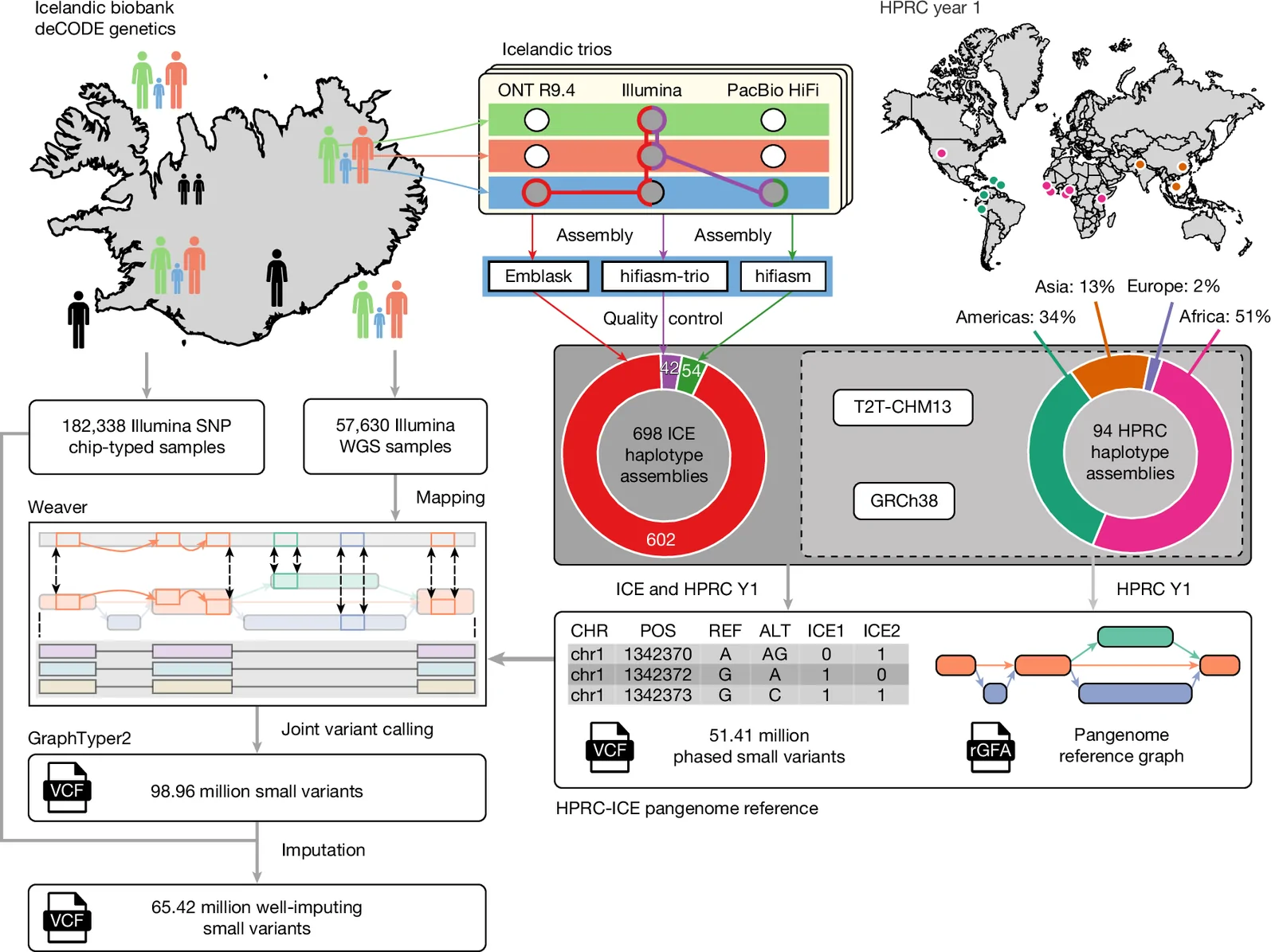
Overview of the study. Long and short reads from sequenced Icelandic individuals were used to create the Icelandic pangenome reference. For this purpose, we developed Emblask to assemble haplotype-resolved dual assemblies with ONT R9.4 and Illumina reads from parent–offspring trios. After quality control, 698 Icelandic haplotype assemblies were combined with the HPRC, GRCh38 and T2T-CHM13 assemblies to form the HPRC-ICE pangenome reference. We developed Weaver to map short reads of 57,630 Icelanders to the HPRC-ICE and subsequently joint-called 98.96 million small variants. We imputed these variants using the long-range phasing of 182,338 chip-genotyped Icelanders, resulting in 65.42 million well-imputing variants. WGS, whole-genome sequencing. Maps downloaded from https://mapsvg.com/ (CC0 1.0).

Here we used the Emblask pipeline to assemble 698 high-quality Icelandic haplotypes and combined them with the HPRC assemblies to create the HPRC-ICE pangenome reference, composed of 788 haplotypes (Fig. 1). Weaver maps to pangenome references composed of hundreds of haplotypes, such as HPRC-ICE, and outputs read mappings in a linear coordinate system such as GRCh38 or T2T-CHM13. As Weaver uses existing file formats, it can simply replace any linear reference mapper in existing analysis pipelines. The linear coordinate system built into the pangenome reference enables direct access to available genome annotations and enables comparisons with linear reference callsets. We showed that Weaver mapping to the HPRC-ICE on a cohort of 57,630 Icelanders resulted in a 6.17% increase of reliably called variants over linear mapping, including variants that associate with disease, demonstrating the recovery of variation previously camouflaged by reference bias.

## Emblask: hybrid dual assembly pipeline

Emblask is a diploid genome assembly pipeline that produces two haplotype assemblies, also called dual assembly, from parent–offspring trio data. Unlike most haplotype-resolved dual assembly tools, which rely primarily on accurate long reads^23,24^, Emblask is a hybrid approach that uses both noisy long reads of the offspring and accurate short reads of all three trio members, specifically Oxford Nanopore Technologies (ONT) R9.4 and paired-end Illumina reads in this study.

Emblask first uses Ratatosk^25^ to perform genome-wide and targeted corrections of long reads using graphs built from the short reads as reference (Supplementary Methods 3). This reduces the long read error rate to less than 1%, while preserving the phase of each read. Next, the corrected long reads are assembled with Flye^26^ into haplotype-resolved sequences of unknown parental origin. At this stage, the assembly is incomplete and fragmented. The long reads are then realigned to the assembly, phased variant calling is performed with PEPPER-Margin-DeepVariant^27^ (PMDV) for error detection and filtering. The haplotype-resolved sequences and corrected long reads mapping to them are assigned to the paternal or maternal haplotypes on the basis of their similarity to the parental short reads. Finally, each set of haplotype-assigned corrected long reads is assembled and polished independently.

**Fig. 2:**
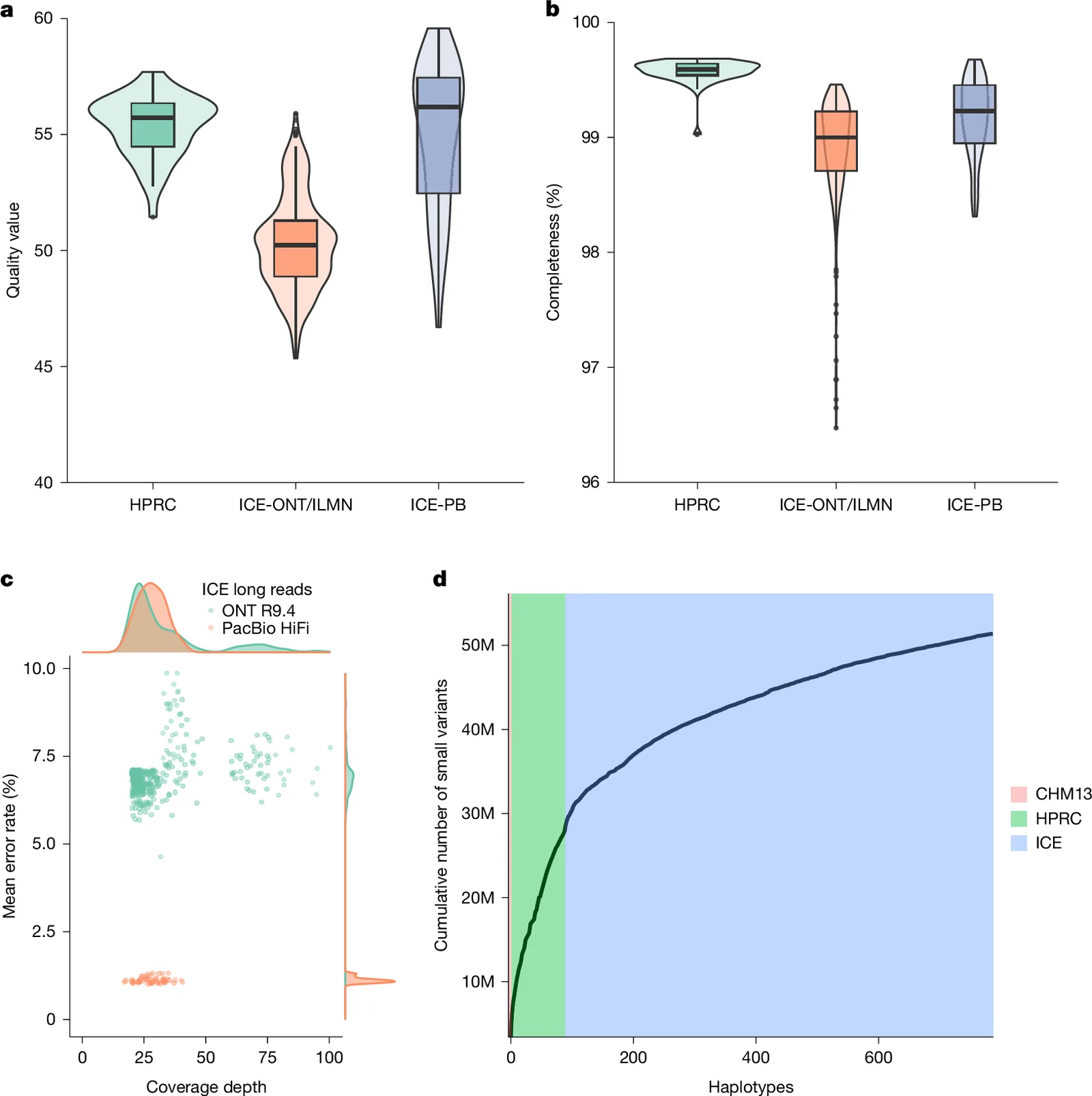
Summary statistics of HPRC and ICE assemblies. **a**, Log-scaled base-level accuracy of the HPRC (*n* = 42), Icelandic ONT plus Illumina (ICE-ONT/ILMN; *n* = 329) and Icelandic PacBio (ICE-PB; *n* = 48) haplotype assemblies. **b**, Percentage of the individual genome sequences correctly captured by the same assemblies shown in **a**. **c**, Mean error rate and coverage depth of the long reads used for the ICE haplotype-resolved dual assemblies. **d**, Cumulative number of small variants added to the HPRC-ICE pangenome reference for each haplotype. Boxes in **a**,**b**, represent the interquartile range (IQR), with the median shown as a line, whiskers extending to values within 1.5× IQR and outliers shown beyond the whiskers.

We evaluated Emblask with HG002 data and compared it to leading ONT R9.4 assembly pipelines. Our results show superior assembly quality and completeness (Q49.35 and 99.01%, respectively). Compared with variants called directly from mapped ONT R9.4, our HG002 assemblies yielded 1.34–9.76% more variants, longer phased regions (6.157 Mb switch NGC50) and more than 30% higher insertion–deletion (indel) accuracy (Supplementary Methods 7). We also assembled offspring haplotypes of 329 Icelandic trios using long reads with a mean error rate of 7% and short reads. The resulting 658 haplotype assemblies had 1 error per 100,000 bases (Q50) and 98.9% completeness on average (Fig. 2a–c). Additional analysis of contiguity, BUSCO (benchmarking universal single-copy orthologues) completeness, false duplications and phasing (Supplementary Methods 6) demonstrates that Emblask hybrid assembly substantially improves upon ONT R9.4-only dual assemblies^28^.

## An Icelandic pangenome

We constructed an Icelandic pangenome reference (HPRC-ICE) from high-quality haplotype-resolved assemblies of Icelanders (ICE), assemblies of individuals with heterogeneous ancestries (HPRC), T2T-CHM13 and GRCh38. The ICE set consists of 698 haplotype assemblies from 361 Icelanders whose genomes were sequenced with Illumina short reads as well as PacBio HiFi (*n* = 49) or ONT R9.4 long reads (*n* = 312). PacBio and ONT–Illumina samples were assembled with hifiasm or hifiasm-trio^23^ and Emblask, respectively. The HPRC set includes 94 high-quality haplotype assemblies (mean Q55.36 and 99.57% completeness) from diverse populations^4^.

The HPRC-ICE pangenome reference is composed of a sequence graph initially built using a single reference assembly, GRCh38 in this study, to which the structural variants from T2T-CHM13 and 94 HPRC haplotype assemblies were incrementally added (Supplementary Methods 8). Since adding structural variants increases graph complexity and the HPRC assemblies were of higher quality and contiguity than the ICE assemblies, we chose not to add structural variants from Icelandic individuals to the graph. In addition, HPRC-ICE contains 51,418,313 phased small variants from the ICE, HPRC and T2T-CHM13 haplotype assemblies (*n* = 788). HPRC-ICE has about twice the number of small variants compared to the HPRC pangenome (Fig. 2d), accounting for 66.7 Mb of inserted or deleted base pairs relative to GRCh38 and 39 Mb of substituted bases. Specifically, 75.87% (39,011,184) of the small variants are SNPs, 22.82% (11,735,493) are indels and 1.30% (671,636) are other types such as multiple nucleotide polymorphisms. Singleton variants represent 36.88% (14,388,640) of the SNPs and 38.87% (4,562,237) of the indels.

## Pangenome mapping with Weaver

Weaver maps short reads to a pangenome reference and outputs alignments within the coordinates of the pangenome reference assembly sequence. The pangenome includes rare small variants whereas previous methods such as Giraffe^17^ and vg-map^29^ recommend excluding variants with allele frequency below 10% and 1%, respectively. Rare variants that are absent in the mapped sample may introduce ambiguity, adversely affect the mapping accuracy and increase mapping time^30^. Yet these variants may strongly affect the regulation of gene expression with significant clinical consequences^31^. We demonstrate that including rare variants in our mapping model enabled the discovery of pathogenic variants in Iceland and in the UK. The negative impact of rare variants in Giraffe can be mitigated by using personalized pangenome references^5^ (GiraffePPR). This approach constructs personalized subgraphs incorporating variants across the entire frequency spectrum based on per-sample *k*-mer counts, thereby improving rare variant detection at the expense of storage and preprocessing computation.

Whereas several previous studies only included either structural variants or small variants in their pangenomes^17,29,32^, the data model used in Weaver represents small variants in phased variant call format^33^ (VCF) and structural variants as a sequence graph in reference Graphical Fragment Assembly (rGFA) format^16^. This dichotomy has several benefits. First, sequence graphs are adapted to represent large and complex variants while VCF is widely used for small variant analysis. This separation avoids the pitfalls of loading all types of variants into a single index, which can drastically increase the structural complexity of the graph and cause longer running time, memory usage and disk footprints^30^. Second, generating a pangenome reference that includes all types of variants is a non-trivial problem that requires extensive computational resources as variants can be nested into larger ones^21,34^. However, pangenome construction can scale to hundreds of human haplotypes if restricted to non-nested small variants.

**Fig. 3:**
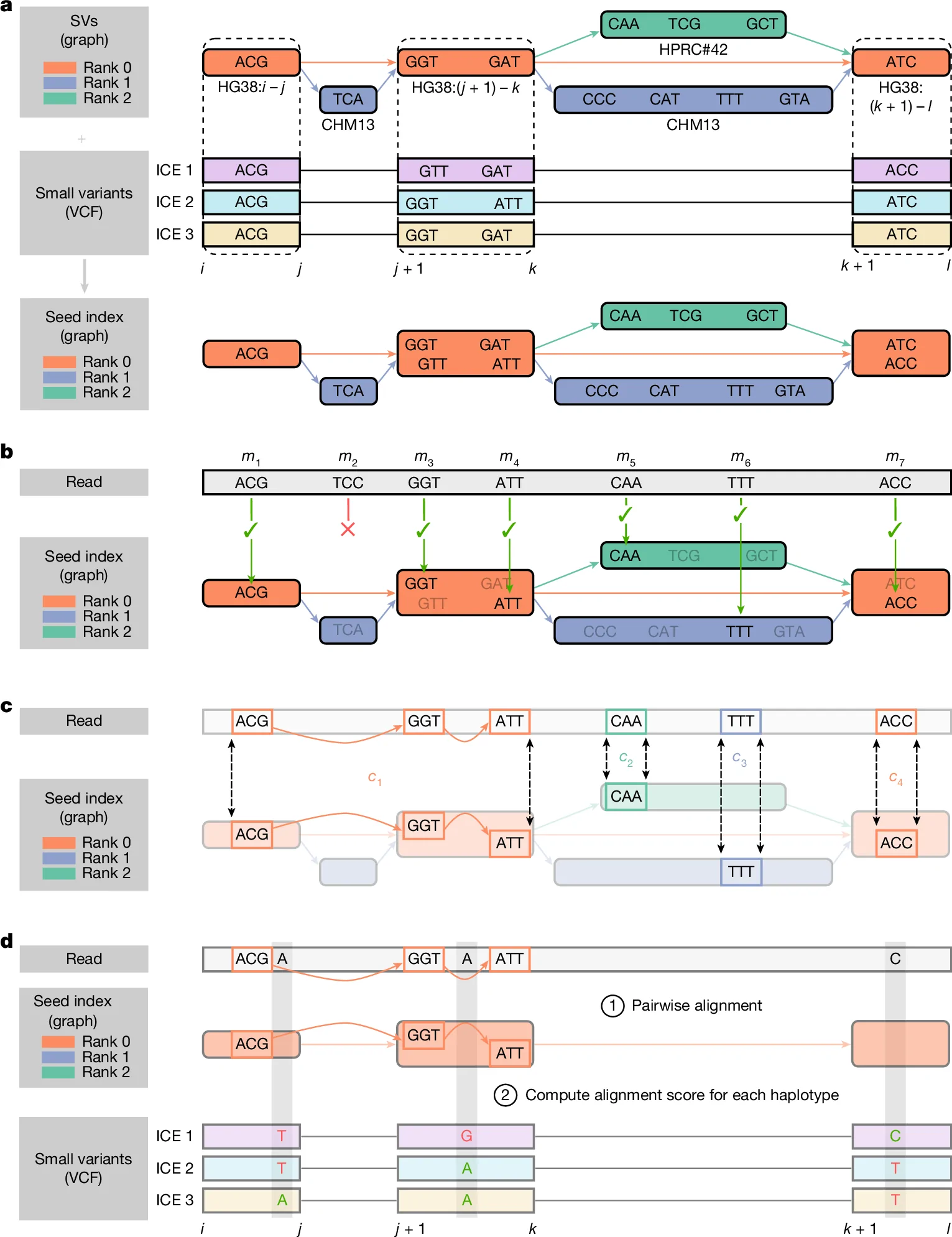
Weaver seed-and-extend mapping algorithm. **a**, A pangenome reference composed of a graph constructed from three haplotype sequences (HG38, CHM13 and HPRC#42) and small variants of three other haplotype sequences (ICE#1–3). Minimizers of length 3 are extracted from the sequences in the graph and the ICE#1–3 haplotype sequences. They are added to a seed index along with their graph location. SVs, structural variants. **b**, Minimizers *m*_1_ to *m*_7_ are extracted from a read sequence and anchored onto the graph using the seed index. **c**, Chains *c*_1_, *c*_2_, *c*_3_ and *c*_4_ are formed by linking together adjacent seeds in the read that match a path of same rank in the graph. **d**, The longest chain or chains (*c*_1_ shown) are extended by walking paths in the graph with same rank as the chain and aligning the spelled-out sequences to the read sequence.

Weaver extends the traditional seed-and-extend mapping paradigm to pangenome graphs using minimizers (Supplementary Methods 2). Minimizers of the read sequence are anchored onto the graph to form seeds (Fig. 3a,b). Vertices in the graph have a rank corresponding to the haplotype assembly that they are a sub-sequence of. Adjacent seeds of a read are chained together if they map along a path of the same rank (Fig. 3c). A chain is progressively extended by traversing paths of the same rank as the chain and aligning the read to the sequences spelled out by these paths. The alignment score of a chain considers all of the phased small variants that overlap the same path as the chain (Fig. 3d). The highest-scoring alignment is selected as the primary sequence.

## Weaver improves variant calling

**Fig. 4:**
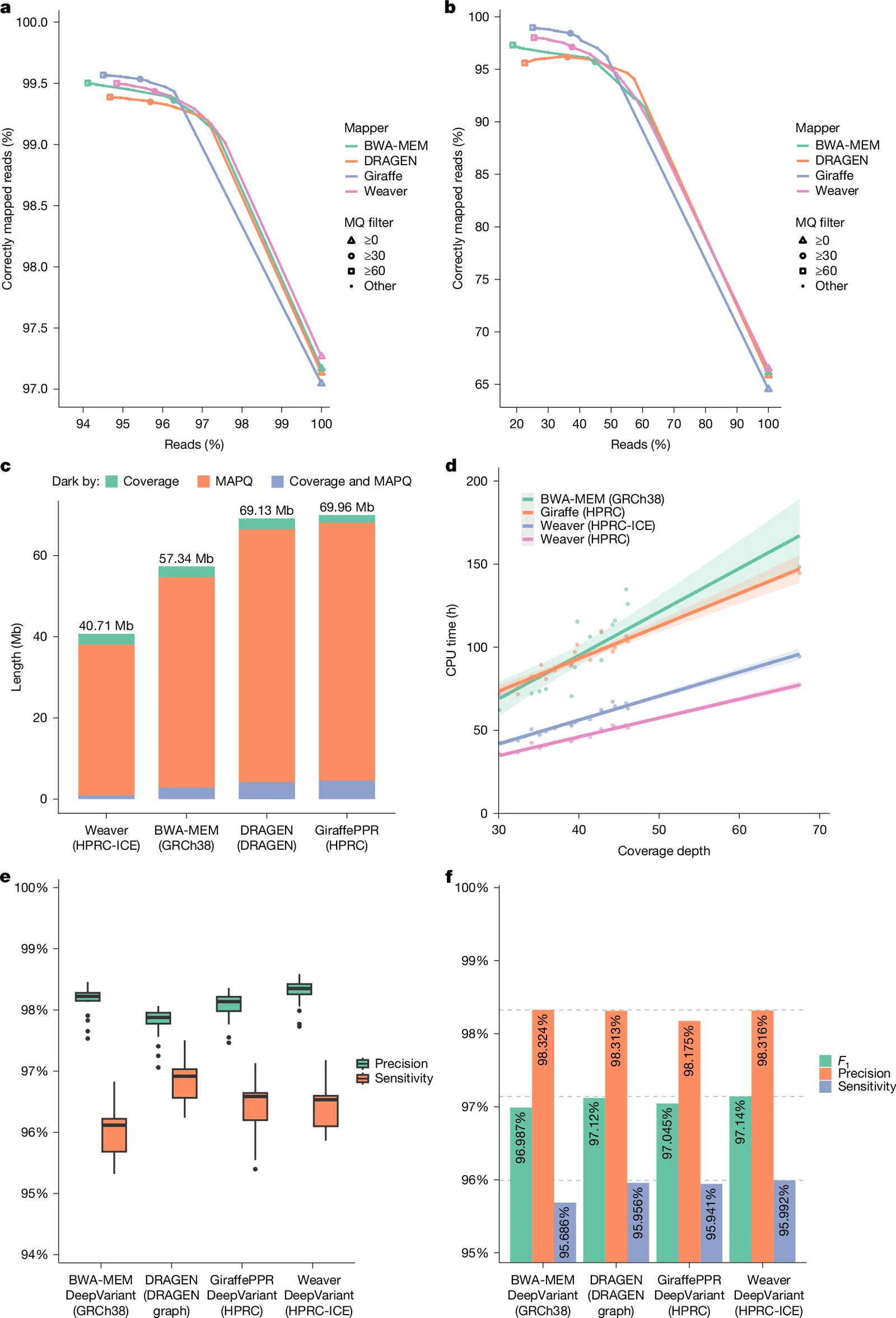
Single sample performance of Weaver-DeepVariant, BWA-DeepVariant, Giraffe-DeepVariant and DRAGEN. **a**,**b**, Proportion of correctly mapped simulated reads using all reads (**a**) and only using reads in the GIAB-stratified low-mappability regions (**b**). The data points represent different mapping quality (MQ) filters: MQ ≥ 0 (all reads), MQ ≥ 5,…, MQ ≥ 60. **c**, Regions stratified as dark and longer than 30 bp computed by aggregating together coverage, base qualities and number of reads with low mapping qualities from the aligned short reads from 19 Icelanders, and then stratifying regions based on average values. **d**, CPU time with respect to short read sequence coverage, measured using 19 Icelanders. Data for DRAGEN are not shown because its calculations are performed with a field programmable gate array rather than a CPU. Solid lines show least-squares linear regression fits with shaded areas representing 95% confidence intervals. **e**, Precision and sensitivity of different mapping methods across the whole genomes of 19 Icelanders. Boxes represent the IQR, with the median shown as a line, whiskers extending to values within 1.5× IQR and outliers shown beyond the whiskers. **f**, *F*_1_ metric, precision and sensitivity of benchmarked methods, genome-wide (high-confidence and low-confidence regions), compared with HG002 T2T-Q100 v1.1.

We benchmarked Weaver against BWA-MEM^35^ (v0.7.16a), DRAGEN^20^ (v4.2.4) and two Giraffe^17^ (v.1.51.0) workflows (Giraffe and GiraffePPR), reporting only the better-performing Giraffe workflow for each experiment. We used the most recently published versions of each method. Mapping performance was evaluated on simulated short reads from both haplotypes of the T2T-Q100 HG002 dual assembly^36^ (Supplementary Methods 9). Compared with Giraffe, DRAGEN and BWA-MEM, Weaver mapped more reads correctly (Fig. 4a), particularly in the Genome in a Bottle (GIAB)-stratified low-mappability regions^37^ (Fig. 4b).

We also benchmarked the mappers on the short reads of 19 Icelanders that were also sequenced with ONT and PacBio. We computed for all mappers the dark regions^38^ in GRCh38 primary assembly scaffolds. Dark regions represent loci with consistently low mapping quality or coverage. These regions represent challenging genomic contexts in which the advantages of pangenome mapping are expected to be the most pronounced. Excluding gaps, 40.71 Mb was dark after Weaver mapping, and 57.34 Mb, 69.13 Mb and 69.96 Mb was dark after BWA-MEM, DRAGEN and GiraffePPR mappings, respectively (Fig. 4c). The 23.32 Mb of regions that were dark after BWA-MEM mapping but not after Weaver mapping overlapped 1,374 protein-coding genes, 61 of which are medically relevant genes^39^, as well as 360 coding DNA sequences among those genes. Weaver mapped the reads to the HPRC-ICE reference in 40% less time than existing methods; Giraffe was the second fastest method (Fig. 4d), despite Weaver mapping reads to more than eightfold more haplotypes.

To evaluate the small variant calling performance of the mappers, we curated a baseline list of safe small variants called from haplotype-resolved dual assemblies made with PacBio HiFi long reads and parental Illumina short reads in 19 Icelandic genomes (Supplementary Methods 8). Small variant calling was performed with DeepVariant^40^ using models tailored to each mapper except for DRAGEN, which embeds its own variant caller. Weaver-DeepVariant performed the best with a mean *F*_1_ score of 97.34% genome-wide, followed by DRAGEN with 97.30%, GiraffePPR-DeepVariant with 97.24% and BWA-MEM-DeepVariant with 97.06% (Fig. 4e). Within high- and low-confidence regions delineated by the GIAB small variant truth set^41^, the *F*_1_ scores of Weaver-DeepVariant are 99.776% and 88.951%, respectively (Extended Data Table 1).

We also assessed the small variant calling performance on HG002 short reads mapped with Weaver to the HPRC-ICE using variants within the autosomal confident regions of the T2T-Q100 HG002 dual assembly^15^ as ground truth (Fig. 4f). The variants were separated into high-confidence and low-confidence regions according to the GIAB HG002 truth set. The methods performance was similar in the high-confidence regions with an *F*_1_ score of 99.36% for DRAGEN, 99.31% for Weaver-DeepVariant, 99.31% for GiraffePPR-DeepVariant and 99.21% for BWA-MEM-DeepVariant. The disparity of results was more distinct in the low-confidence regions with an *F*_1_ score of 83.28% for Weaver-DeepVariant, 82.79% for DRAGEN, 82.70% for BWA-MEM-DeepVariant and 82.62% for GiraffePPR-DeepVariant.

Although we were unable to evaluate GiraffePPR with the HPRC-ICE reference owing to the high number of haplotypes, we mapped reads with Weaver to the HPRC pangenome and still achieved a superior *F*_1_ score (83.26%) in low-confidence regions over existing methods (Supplementary Methods 13). This indicates that these variant calling improvements can be attributed both to the mapping algorithm and the extended pangenome reference.

## Weaver scales to biobank-size analysis

From this point onward, we no longer compare methods under identical inputs. Instead, we fix the methods to quantify the consequences of replacing a linear reference with a pangenome reference. Based on the above benchmarks, our methods offer improvement over existing state-of-the-art mapping methods in terms of computing resources, mapping accuracy and variant calling performance. We therefore selected Weaver mapping to HPRC-ICE for our next cohort of Icelanders. The cohort consists of 57,630 whole-genome-sequenced Icelandic samples, including 6,286 parent–offspring trios, 446 monozygotic twin pairs and 355 duplicated samples. We compared our results to a previous cohort of Icelanders mapped to a linear reference genome with BWA-MEM.

**Fig. 5:**
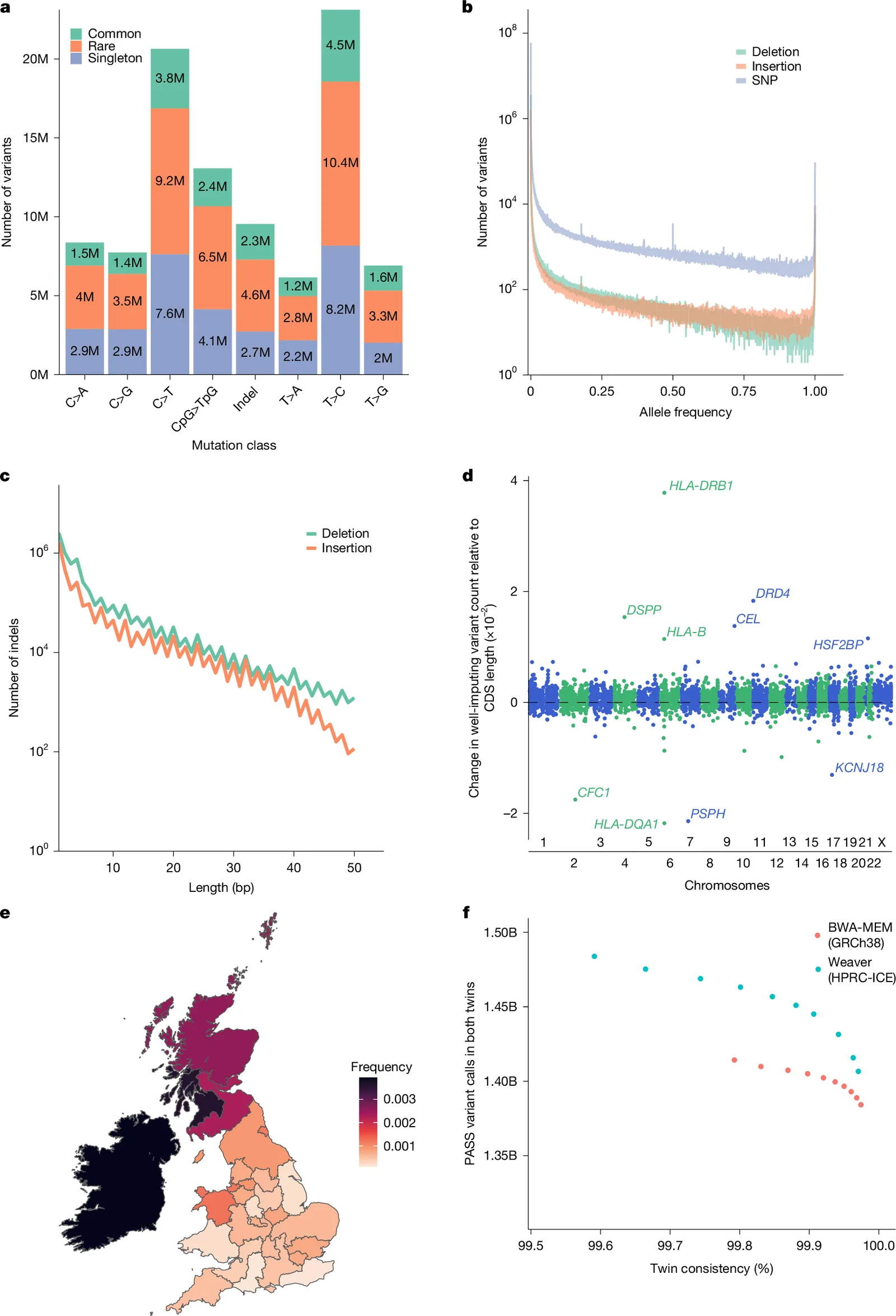
Weaver callset of 57,630 Icelanders mapped to HPRC-ICE. **a**, Distribution of variant counts by mutation class. The counts of each mutation class are partitioned by frequency: singleton; rare (not singleton and allele frequency <0.1%); and common (allele frequency ≥ 0.1%). **b**, Allele frequency distribution for the SNPs and indels. **c**, Indel length distribution. **d**, Change in well-imputing variant counts of protein-coding genes in the Weaver set compared with the BWA-MEM set, relative to coding DNA sequence (CDS) length. Only genes with connections to phenotypes in the OMIM database are shown (*n* = 4,905). **e**, Minor allele frequency geographical interpolation of chr. 21:42861633:G>T (GRCh38) in the British Isles. **f**, Consistency of genotype calls among the same monozygotic twin pairs (*n* = 88) in two Icelandic callsets, using different genotype quality thresholds. B, billion. Map in **e** downloaded from https://gadm.org/maps/GBR.html.

Running the mapping pipeline on this cohort, including Weaver mapping, alignment sorting, CRAM compression and Crumble^42^ quality value compression, took 9.37 million central processing unit (CPU) hours for samples with a mean coverage of 38.94× (standard: 9.29×). Small variant calling was then performed from the produced alignments with GraphTyper^32^ on the autosomes and chromosome X in 0.9 million CPU hours, resulting in 98,961,991 variants after applying the recommended GraphTyper filters (Supplementary Methods 4). Compared to the single-sample evaluations for which DeepVariant was the most appropriate variant caller, GraphTyper is designed for the joint genotyping of population-scale cohorts and places less emphasis on single-sample calling performance. Furthermore, genotyping the cohort with DeepVariant was estimated to require an order of magnitude more computation time than GraphTyper, exceeding 10 million CPU hours, and therefore GraphTyper was used for this task, consistent with our previous cohort of Icelanders. Most variants we genotyped were uncommon across all mutation classes (Fig. 5a), with 34.71% of the SNPs being singletons and 46.28% being rare (allele frequency less than 1% but not a singleton). Similarly, 28.53% and 47.81% of the indels were singletons and rare, respectively (Fig. 5b). As expected, we observed a periodic pattern of indel sizes, attributable to highly mutable short tandem repeats^43^ (Fig. 5c).

Our Icelandic Weaver-GraphTyper callset performed well across various quality assessments. The ratio of transition to transversion was 1.94, close to the expected 2.0–2.1 for genome-wide sets in humans^44^, although lower ratios may be seen in large cohorts owing to the saturation of transitions. We also assessed the genotype calls with genotype quality above 40 in parent–offspring trios, monozygotic twins and duplicated samples. In particular, the callset had a 99.980% Mendelian consistency rate for offspring genotype calls in trios for which both parents have homozygous genotype calls. Additionally, the monozygotic twins and duplicated samples genotype consistencies measure the proportion of matching genotypes in pairs of twins and duplicated samples for which a sample in the pair has a non-reference genotype. In our set, variants have a mean genotype consistency of 99.969% and 99.967% for monozygotic twins and duplicated samples, respectively.

GraphTyper uses a logistic regression model based on variant calling metrics to assign a score, denoted AAscore^7^, to each variant representing the probability of a true variant call. We refer to variants with AAscore greater than 0.5 as reliable. We compared the reliable small variant calls of 57,630 Icelandic samples, mapped with Weaver to the HPRC-ICE pangenome reference, to the reliable small variant calls of our previous variant calling iteration composed of 63,460 Icelandic samples, mapped with BWA-MEM to the GRCh38 linear reference. These sets, referred to as the Weaver and BWA-MEM sets respectively, share 47,306 samples with 10,324 additional samples unique to Weaver and 16,154 additional samples unique to BWA-MEM. Both sets were genotyped with GraphTyper and comparisons are restricted to the primary scaffolds for the autosomes and the chromosome X in GRCh38.

**Extended Data Fig. 1:**
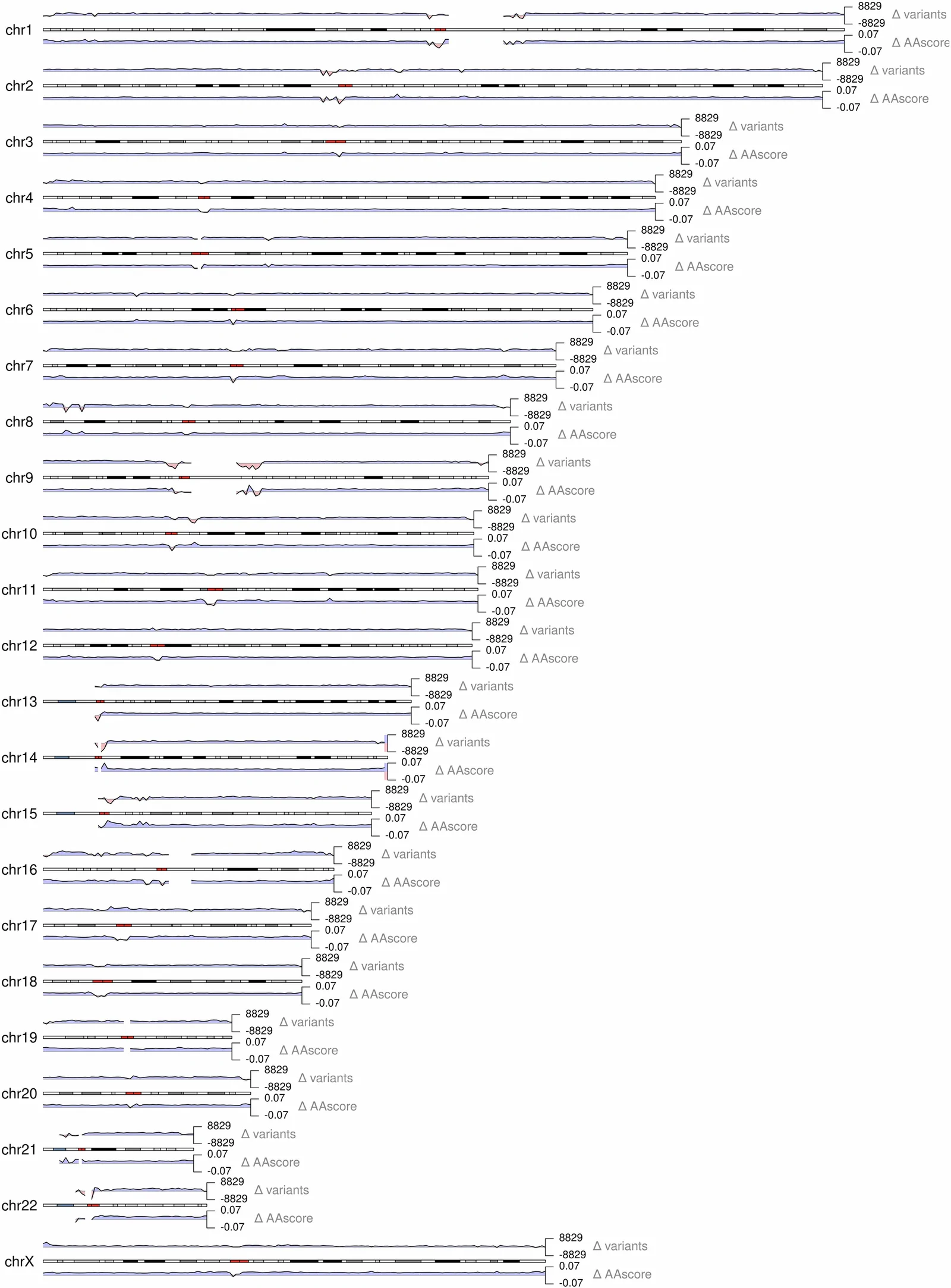
Difference of AAscore and number of variants between the reliable callset of 57,630 Icelandic samples mapped with Weaver to the HPRC-ICE and the reliable callset of 63,460 Icelandic samples mapped with BWA-MEM to GRCh38. A blue area corresponds to an increase of mean AAscore (bottom panels) or number of reliable variants (top panels) in the Weaver set with respect to the BWA-MEM set while a read area shows a decrease. The mean AAscores and number of variants differences were stratified by non-overlapping windows of 1 Mbps.

The Weaver variant set contained 6.17% (5,751,644) more reliable small variants than the BWA-MEM set despite having 5,830 fewer samples (Fig. 5d). Most loci across all chromosomes in the Weaver set exhibited an improved AAscore, with a mean of 0.937 compared with 0.915 in the BWA-MEM set (Extended Data Fig. 1). The increase of the mean AAscore between the variants of the two sets was similar across exonic, intronic and intergenic regions. Only regions near centromeres and telomeres showed no improvement, as these regions are difficult to map to with short reads because of their high-order repetitive nature, structural complexity and GC content profile (Extended Data Fig. 1). The increase in the number of reliable variants was the largest in intergenic regions as they are less conserved than exons.

We analysed 88 monozygotic twin pairs present in both sets using the same reads. In those samples, the Weaver set contained 4.9% more reliable variants in which at least one sample was a variant carrier. The Weaver set had more consistent calls but lower twin consistency rate (Fig. 5f), probably owing to the increase of variants in low-mappability regions where calls are less confident. After applying genotype quality filters, the Weaver set contained more passing variant calls for the same twin consistency rates.

The Weaver set also contained an additional 483,408 reliable variants in 858 contigs that are not primary scaffolds in GRCh38 and for which 90% of the samples have at least 50 reads mapping to them. These supplementary contigs add 15.14 Mb of sequences to GRCh38, 69% of which are non-dark. Because of their variable ploidy, lack of available functional annotations and limited support in downstream methods, we excluded them from other analyses.

The unfiltered variant calls in the Weaver and the BWA-MEM sets were imputed into a larger set of chip-genotyped individuals using long-range phasing^45^. The number of chip-genotyped Icelanders was 173,025 for the BWA-MEM set and 182,338 for the Weaver set. Despite containing fewer sequenced samples, the Weaver set contained 11.88% (6,947,790) more well-imputing variants across the genome and 9.96% (53,999) more in coding regions. We then restricted our analysis to well-imputing variants with a minimum frequency of 10^−5^ in coding regions for both sets, for which the Weaver set contained 6.27% (26,100) more than the BWA-MEM set. Across protein-coding genes, the Weaver set had more such variants than in the BWA-MEM set for 11,237 genes, whereas 3,843 genes had more such variants in the BWA-MEM set compared to the Weaver set. In particular, 66 genes in the Weaver set and 28 genes in the BWA-MEM set had no such variants in the other set. Several members of the USP17L family of genes contain up to 25 times more variants than in the BWA-MEM set. Genes with a connection to a phenotype in the Online Mendelian Inheritance in Man (OMIM) database^46^ displayed up to three times more variants, such as *EMD*, *TNNI3*, *MDM2* and *CBS*, while genes such as *DSPP*, *HLA-DRB1*, *CEL* and *DRD4* had the largest increase of variant counts with respect to the length of their coding regions (Fig. 5d). The Weaver set also contained 2.51% more variants (38) classified only as pathogenic or likely pathogenic in ClinVar^47^ than the BWA-MEM set.

## Weaver uncovers disease associations

More than 76 SNPs and indels in *GBA1* are classified in ClinVar^47^ as ‘pathogenic’ or ‘likely pathogenic’ for Gaucher’s disease and Parkinson’s disease. Yet *GBA1* has proved difficult to access from short reads because of the *GBAP1* pseudogene, which shares 96% sequence identity with the *GBA1* coding sequence. Long reads have been used^48,49^ to overcome the high sequence similarity of the two genes in attempts to find pathogenic variants.

**Extended Data Fig. 2:**
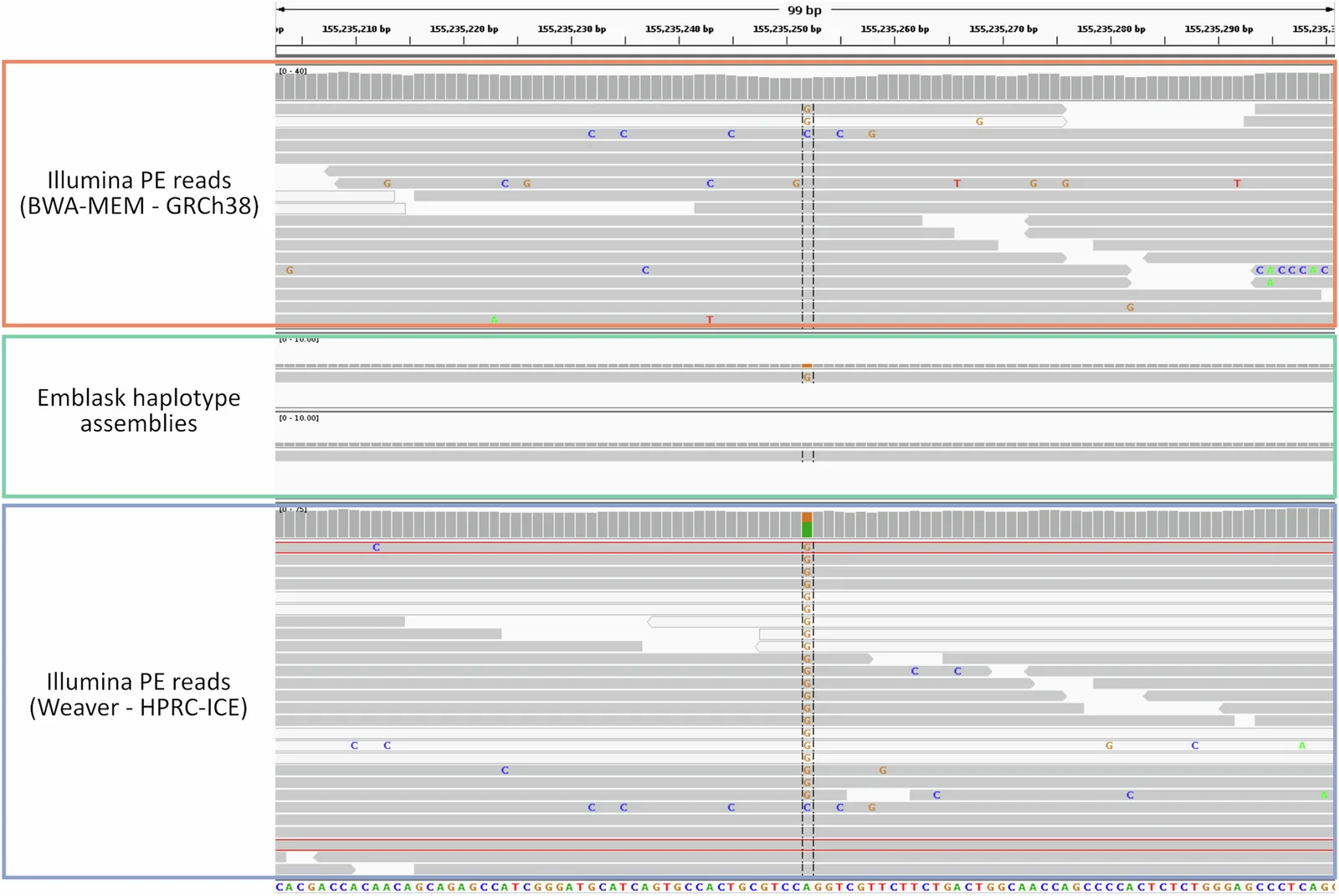
IGV visualization of mapped Illumina PE reads from an Icelandic carrier of p.Leu483Pro in GBA1. Grey horizontal bars are mapped reads with mapping quality (MQ) ≥ 0 while white horizontal bars are ambiguous mapping with MQ = 0. Top: Illumina PE reads mapped with BWA-MEM to GRCh38. Only one read with MQ > 0 supports the p.Leu483Pro allele. Middle: Haplotype assemblies made from Illumina PE and ONT R9.4 reads with Emblask. One haplotype supports the p.Leu483Pro allele. Bottom: Illumina PE reads mapped with Weaver to the HPRC-ICE pangenome ref. ^13^ reads with MQ > 0 support the p.Leu483Pro allele.

By mapping to a pangenome rather than a linear reference, we uncovered missense variants in *GBA1* that were systematically inaccessible to short read analyses based on GRCh38. Among those, chromosome (chr.) 1:155235252:A>G (GRCh38) encoding p.Leu483Pro had been documented as pathogenic for Parkinson’s disease in ClinVar and OMIM. It was reliably called from Weaver alignments (Extended Data Fig. 2) with a risk allele frequency of 0.22%, but was not found in our Icelandic BWA-MEM set or in the UK Biobank^7^. The risk allele failed quality control filters in gnomAD^9^ 4.1.0 and was underrepresented in European (non-Finnish) ancestry with only 89 risk alleles, whereas 2,600 were expected based on our estimates in Iceland. The allele was present in two of the ICE haplotypes in the HPRC-ICE small variant set but not in the HPRC haplotypes. The imputed p.Leu483Pro variant has an imputation information^50^ of 0.98 and associated with Parkinson’s disease (*P* = 2.94 × 10^−6^, *χ*^2^ test, odds ratio (OR) = 2.55, 95% confidence interval [1.72, 3.78]) and early-onset Parkinson’s disease under 60 years of age (*P* = 4.69×10^−8^, *χ*^2^ test, OR = 7.33, 95% confidence interval [3.12, 17.20]). The variant was missed or failed quality filters in the short reads of the two Icelanders whose assemblies had the variant after mapping and genotyping with BWA-MEM-DeepVariant, GiraffePPR-DeepVariant and DRAGEN. The other uncovered missense SNP, chr. 1:155235217:C>G (GRCh38), was rare (allele frequency = 0.010%) and did not associate with any Parkinson’s disease phenotypes in Iceland.

We remapped the short reads overlapping *GBA1* and *GBAP1* for 429,193 British and Irish participants in the UK Biobank using Weaver to the HPRC-ICE. After variant calling, p.Leu483Pro was found in the new callset with allele frequency 0.10%. We successfully replicated our association of the variant to Parkinson’s disease (*P* = 1.25 × 10^−11^, *χ*^2^ test, OR = 4.91, 95% confidence interval [2.58, 9.34]). This association supports the hypothesis that p.Leu483Pro is present in the UK, although it was missed or had failed the cohort quality filters in both the BWA-MEM and the DRAGEN mappings^6^ to GRCh38. The combined association to Parkinson’s disease in both Iceland and the UK Biobank was *P* = 1.30 × 10^−15^, *χ*^2^ test and OR = 3.36 (95% confidence interval [2.49, 4.53]).

Homocystinuria is an autosomal recessive disorder that is characterized mainly by eye and skeletal anomalies, abnormal vascular events and issues with the central nervous system^51^. The missense SNP chr. 21:43062988:C>T (GRCh38) encoding p.Gly307Ser in *CBS* has been identified as a common cause of homocystinuria in patients with Celtic ancestry^52^. It is also well characterized as pathogenic in ClinVar and OMIM but located within a low-mappability region. While the variant was not called in our BWA-MEM set nor in the UK Biobank, and was filtered out of gnomAD due to the low quality of its genotypes, it was present in three of the Icelandic haplotype assemblies in HPRC-ICE. The variant was called in the Weaver set, then it was imputed in Iceland with an imputation information of 0.95 and allele frequency 0.31%. The only homozygous carrier in the imputed set had been previously diagnosed with a disorder of sulfur-bearing amino acid metabolism, which includes homocystinuria, and presents several phenotypic features of homocystinuria. We found that p.Gly307Ser was in strong linkage disequilibrium (*r*^2^ = 1.00 in HPRC-ICE, *r*^2^ = 0.91 in the Weaver set) with a non-coding SNP (chr. 21:42861633:G>T in GRCh38) located downstream of the segmental duplication encapsulating *CBS*. As a result, this variant is not located in a low-mappability region. It imputes similarly in our two Icelandic callsets (both have allele frequency 0.36% and imputation information greater than 0.99) and we found it in the UK Biobank BWA-MEM callset (allele frequency 0.097% in British and Irish groups) with no homozygous carriers. It was more common in Ireland and Scotland than in other parts of the British Isles (Fig. 5e), consistent with p.Gly307Ser being more common among individuals of Celtic ancestry^52^.

## Discussion

Pangenome references instigate a major paradigm shift in the whole-genome sequencing era by unlocking difficult to access loci of the human genome and enabling scientists to fully exploit the potential of short read sequencing. However, the complexity of pangenome references presents many challenges that have prevented their widespread usage, especially at a population scale. In this work, we introduced two practical and scalable methods, Emblask and Weaver, to replace linear references with pangenome references. Emblask and Weaver seamlessly integrate in existing analysis pipelines with minimal modifications. Using these new tools, we demonstrated the potential of pangenome references to discover pathogenic variants that were previously concealed due to the reference bias caused by linear mapping.

We constructed the HPRC-ICE pangenome reference from 788 haplotype assemblies, from which we called 51.41 million small variants. This pangenome reference replaces GRCh38 to enable variation-aware mapping with Weaver while still producing alignments in GRCh38 coordinates, retaining compatibility with existing annotations and downstream analysis tools. In our benchmarks, Weaver consistently improved mapping performance, particularly in low-mappability regions. Additionally, it mapped reads faster than other mappers, despite having an order of magnitude more haplotypes in its reference than the other mappers. In our benchmark of 19 samples, Weaver called 11.5% fewer false positives and required 40% less time than GiraffePPR, which was the best performing previous method. Our contributions in the Emblask assembly pipeline, the Weaver mapper and the Icelandic pangenome reference HPRC-ICE are made available to the research community.

Mapping the short reads of 57,630 Icelanders to HPRC-ICE enabled the discovery of pathogenic variants that were camouflaged in segmental duplications. We identified a previously undetected *GBA1* variant association to early-onset Parkinson’s disease in Iceland and in the UK Biobank. We also uncovered a SNP in *CBS* that is likely to be pathogenic for homocystinuria in an Icelandic participant. These findings demonstrate that reference bias is not a minor issue but a systematic impediment to comprehensive genetic analyses of diseases. Both uncovered variants were detected only in Icelandic assemblies, emphasizing the importance of including rare variants in the pangenome and building pangenomes from sequenced individuals affected with genetic disorders. This endeavour introduces several complications due to the increased risk of false mappings to rare sequences. A promising direction towards solving this issue is the usage of a personalized pangenomes^5^ in which pangenome sequences unrelated to the sequenced individual are not considered. In addition, although ONT R9.4 is being discontinued for the more accurate R10 chemistry, our hybrid assemblies with Emblask reached Q50 quality and near 99% completeness, indicating that they can be included in any future pangenome built on ONT R10 assemblies. With our contributions offering a readily available solution to replace reference genomes with pangenome references at population scale, we are looking forward to integrating personalized pangenomes and ONT R10 assemblies into our pangenome pipeline to enhance HPRC-ICE with the increased genetic diversity of the next HPRC pangenome iteration. Together, our results demonstrate that population-scale pangenome mapping is not a technical refinement but a necessity for comprehensive discovery of disease variants from short reads.

## Methods

### Pangenome reference

The pangenome reference used in this study is a sequence graph augmented with phased small variants. It is stored in two data files: a sequence graph in reference rGFA format^16^ containing the structural variants and a supplementary phased VCF^33^ file containing small variants. The rGFA sequence graph is constructed first from haplotype assemblies, then the small variants are extracted from the assemblies and added to the VCF file^30^.

The sequence graph *G*(*V*,*E*) has vertices *v* *ϵ* *V* and edges *e* *ϵ* *E*. A segment is a sub-sequence *s* of one or more input haplotype assemblies. Each segment is made of two vertices, having respectively the forward and reverse directions. Traversing the forward vertex spells out *s* while traversing the reverse vertex spells out its reverse complement. An edge *e* connects two segments if their respective sub-sequences are adjacent in one or more input assemblies. The edges are bidirected such that each end of an edge is either pointing forward or reverse. The direction determines the traversal directions of the connected segments. Thus, there are four forms of edges: forward–forward, forward–reverse, reverse–forward and reverse–reverse.

The rGFA graph is incrementally constructed from a set of input haplotype assemblies (Supplementary Methods). The first input assembly serves as the reference assembly, which is the basis of the coordinate system. When another haplotype assembly is inserted in the graph, existing segments might be split up and additional vertices with new sequences introduced. The sequences spelled out by paths in the graph from a previous insertion can always be spelled out in the updated graph. Since these sequences do not change, they are denoted stable. To keep the sequences stable across insertions, we give each edge and vertex a rank corresponding to the input haplotype assembly that it is originally from. Traversal of a path of rank *r* spells out a sub-sequence in the *r*-th haplotype assembly sequence.

### Weaver

Weaver maps paired-end short reads in FASTQ format to a pangenome reference, such as HPRC-ICE. The reference can either be linear in FASTA format or a graph in rGFA format with an optional phased VCF file containing small variants. It outputs alignments in Sequence Alignment/Map (SAM) format^53^, typically compressed as a BAM or CRAM file. Before mapping, Weaver preprocesses the pangenome reference once in an indexing step.

#### Indexing

The Weaver index has two components. The first component is a seed index that stores the minimizers found in the pangenome reference alongside their locations. A minimizer is a sub-sequence of length *k* (23 by default), called a *k*-mer, with the minimum hash value in a sliding window containing *w* overlapping *k*-mers (11 by default). We use the minimap2 (ref. ^54^) hash function, which returns the same hash value for a *k*-mer and its reverse complement. As a result, the same set of minimizers is extracted regardless of the orientation of sequences in the pangenome reference. The minimizers are extracted from all haplotype sequences represented in the graph and in the VCF. They are then added to a seed index along with their graph locations (Fig. 3a). The minimizers may therefore contain bases from alternative alleles in the VCF file. The second component is the ICU index, which stores all pairs of vertices that see each other. We define a vertex *u* as seeing vertex *v* if there exists a path from *u* to *v* in the graph, which is *d* bp (*d* = 1,500 by default) or less.

#### Mapping

During Weaver read mapping, minimizers are extracted from the read sequence in the same way as during indexing. The minimizers are then anchored to the graph using the seed index (Fig. 3b). Weaver links together seed index hits to form chains (Fig. 3c) when the seeds anchor onto vertices of same rank and are interspersed by the same distances in the read and the graph. If both reads in a pair have long chains that see each other, other small chains that do not see chains on the other read are discarded (Supplementary Methods 2). The chains are then extended along the stable sequence by traversing paths of the same rank in the graph. Then we perform a pairwise alignment between the stable sequence and the read sequence (Fig. 3d). An alignment score *S* is calculated as

1 $${S}_{h}=A{n}_{{\rm{m}}{\rm{a}}{\rm{t}}{\rm{c}}{\rm{h}}}-B{n}_{{\rm{m}}{\rm{i}}{\rm{s}}{\rm{m}}{\rm{a}}{\rm{t}}{\rm{c}}{\rm{h}}}-C{n}_{{\rm{c}}{\rm{l}}{\rm{i}}{\rm{p}}}-{O}_{{\rm{g}}{\rm{a}}{\rm{p}}{\rm{O}}{\rm{p}}{\rm{e}}{\rm{n}}}-E{n}_{{\rm{g}}{\rm{a}}{\rm{p}}{\rm{E}}{\rm{x}}{\rm{t}}{\rm{e}}{\rm{n}}{\rm{d}}}$$

where *h* is the haplotype sequence, and *A* = 1, *B* = 4, *C* = 6, *O* = 7 and *E* = 1 by default. *n*_match_ and *n*_mismatch_ are the numbers of sequence matches and mismatches in the alignment, respectively. *n*_clip_ is the number of soft clips at the beginning or end of the read sequence. Gaps in the alignment are penalized using an affine cost, where *n*_gapOpen_ and *n*_gapExtend_ are the numbers of gap openings and extensions, respectively. We choose the alignment maximizing this score with *h* as the stable haplotype sequence and, in case of a tie, we use the one that has gaps as far left as possible.

#### Variation-aware scoring

After all chains have been aligned, Weaver estimates which of them are most likely at their correct genomic location. Comparing the previously calculated alignment scores would be biased towards the arbitrary selected stable sequence. Instead, Weaver calculates a weighted average alignment score, *wS*, across all haplotype sequences *h* *ϵ* *H*

2 $${wS}=\sum _{h{\epsilon }H}P(h| {S}_{h}){S}_{h}$$

where the weight *P*(*h*|*S*_*h*_) is the probability that the read was sequenced from haplotype sequence *h*. Common variants are observed in many haplotypes and thus impact the alignment score more than rare variants. The haplotypes *h* *ϵ* *H* include the stable sequence and all the sequences represented in the VCF file. Weaver selects the alignment with the maximum *wS* as the primary alignment.

### Emblask

Emblask is a diploid genome assembly pipeline that produces a set of two haplotype assemblies, also called dual assembly, from the offspring of a parent–offspring trio (Supplementary Methods 3). The method is a hybrid approach using both noisy long reads and accurate short reads, specifically ONT R9.4 and paired-end Illumina reads in this study. Emblask takes as input long reads for the genome of the offspring as well as short reads for the three members of the trio.

In the following, we refer to long reads as LRs and short reads as SRs. We define cov(*A*) and cov(*A*,*s*) as the LR coverage of assembly *A* and the coverage of sequence *s* ∈ *A*, respectively. Phasing refers to mapping LRs to a sequence or a set of sequences, calling small variants with PMDV^27^ and assigning the alleles of the called heterozygous variants to a haplotype *H*_1_ or *H*_2_ using the LR overlap between adjacent variants. A phase set delineates a region in which two or more heterozygous variants are phased. In a phase set, the haplotype for which the phased variants have the most reference alleles is the reference haplotype *H*_*r*_ while the other haplotype is the alternate haplotype denoted *H*_*a*_. Haplotagging refers to assigning a haplotype tag *H*_1_ or *H*_2_ to LR alignments in a phase set based on the phased variants they overlap.

#### Global error correction

The first step in Emblask is to decrease the LR error rate using the SRs with Ratatosk^25^. After correcting the LRs, Emblask trims sub-sequences with low correction scores reflecting uncorrected or low-quality corrected bases.

#### Collapsed assembly

Emblask assembles the corrected LRs into a collapsed assembly *A*_*c*_ with Flye^26^. Each assembled sequence, called a contig, contains a combination of alleles from the paternal and maternal haplotypes. Each locus is therefore represented in at most one contig.

#### Local error correction

Emblask improves the corrected LR error rate by mapping SRs and corrected LRs to the contigs of *A*_*c*_ to perform local corrections. Non-overlapping segments of 200 kb are defined on *A*_*c*_ to split the read alignments into different windows that are corrected separately. A paired-end SR can occur in multiple windows if it cannot be mapped uniquely on *A*_*c*_.

#### Haplotype-resolved assembly

Emblask assembles the corrected LRs into a haplotype-resolved assembly *A*_*r*_ with Flye. The output haplotigs represent sub-sequences of the paternal or maternal haplotype but without distinction to which of the two haplotypes each sequence is from. Furthermore, the assembly is still fragmented and incomplete because for any locus, only one of the two parental haplotypes might have been assembled into a haplotig.

#### Haplotig cleaning

Each haplotig must be assigned to either the paternal or maternal haplotype to create the final assembly. Errors in the haplotigs can lead to an incorrect parental assignment that would result in fragmentation, gaps and false duplications in the final assembly. Therefore, haplotigs must be filtered, split and polished before performing the parental assignment. The coverage of each haplotype assembly is initially expected to be half the coverage of the haplotigs in *A*_*c*_:

3 $$c=\frac{{\rm{c}}{\rm{o}}{\rm{v}}({A}_{c})}{2}.$$

Corrected LRs are mapped to *A*_*r*_ and only haplotigs *h*′ with coverage within the range $\frac{c}{2} < \mathrm{cov}({A}_{r},{h}^{{\prime} }) < 3c$ are kept to eliminate haplotigs that are the result of erroneous duplications or collapsing during assembly. Corrected LRs mapping to the remaining haplotigs are then phased and haplotagged with Margin^55^. Haplotigs with coverage greater than 50% of *c* are annotated as collapsed coverage (CC) candidates. CC candidates containing multiple phase sets are split between phase sets to ensure haplotype phasing integrity. CC candidates are also split by removing the sub-sequences of phase sets for which at least 25% of the heterozygous SNPs have a phase inconsistent with the reference and alternate haplotypes. The resulting haplotigs are then polished with Flye using only the untagged and reference-tagged alignments. Additional fine-grained haplotig cleaning takes place by refining the expected haplotype coverage with the coverage of high-quality phased SNPs in the polished haplotigs. The final set of cleaned and polished haplotigs is denoted *A*_*r*_′.

#### Haplotig trio binning

A set of haplotigs *B*_*r*_′ that closely approximates the missing haplotigs of *A*_*r*_′ is produced by haplotagging LRs with respect to *A*_*r*_′ and polishing haplotigs of *A*_*r*_′ with the alternate-tagged and untagged alignments. Haplotigs of *A*_*r*_′ and *B*_*r*_′ are then assigned to the paternal or maternal haplotype with which they share the most sub-sequences. For any trio-binned haplotig *h*_*a*_ in *A*_*r*_′, if there exists an approximated alternate haplotig *h*_*b*_ in *B*_*r*_′ assigned to the same parent as *h*_*a*_, the parental assignment with the lower confidence is flipped (Supplementary Methods 3).

#### LR trio binning

Haplotigs in *A*_*r*_′ have been assigned to a parental haplotype but *A*_*r*_′ is still incomplete and fragmented. To resolve both issues, LRs are mapped to *A*_*r*_′, phased and haplotagged. For each haplotig assigned to a parental haplotype *H*, LRs from the reference-tagged primary alignments are assigned to *H* while LRs from alternate-tagged primary alignments are assigned to the other parental haplotype. LRs from untagged primary alignments outside of phase sets are assigned to *H* or evenly distributed between the two parental haplotypes if the local coverage indicates the presence of two haplotypes (Supplementary Methods 3).

#### Dual assembly

Each group of LRs assigned to either parental haplotype is assembled independently with Flye, resulting in two preliminary haplotype assemblies, which are filtered further with the aim of removing erroneous duplications caused by incorrect parental assignment. Each haplotype assembly is then polished with the LRs and the SRs.

### HPRC-ICE

The HPRC-ICE pangenome reference is first composed of the HPRC year 1 pangenome graph^4^ built with Minigraph^16^ using GRCh38 as the reference assembly. Non-reference vertices present in fewer than nine assemblies were removed from the graph. The HPRC-ICE is also composed of a VCF file representing phased small variants called from the ICE, HPRC Y1 and T2T-CHM13 assemblies. In the following, we refer to the Icelandic assemblies created from PacBio HiFi reads as ICE-PB and the assemblies created from ONT-Illumina reads as ICE-ONT/ILMN (Supplementary Methods 5).

#### Genome assembly

All PacBio HiFi samples were assembled using hifiasm or hifiasm-trio if the parental short reads were available. All ONT R9.4 samples were assembled with trio short reads using the Emblask pipeline (Supplementary Methods 3, 5 and 6). ONT datasets were automatically downsampled to 50× by Emblask prior to each assembly step in the pipeline to keep running time and memory usage tractable.

#### Variant calling

Phased small variants were called from all the ICE dual assemblies with a modified version of dipcall^56^ using minimap2 (ref. ^54^) v2.24 and wider *z*-drop score parameters to improve the contiguity of the assembly alignments^57^. The output variant calls were left-aligned and normalized, and multi-allelic variants were split into bi-allelic. Furthermore, all structural variants, variants with a star allele and variants with a missing genotype or a half-missing half-reference were filtered out. Variant calls from the ICE-ONT/ILMN assemblies were then filtered and polished using the trio short reads (Supplementary Methods 8). Finally, each diploid genotype was split into two haploid genotypes, one for each haplotype assembly.

#### Quality control

Small variants from the ICE haplotype assemblies fulfilling all the following criteria were merged into the HPRC-ICE set: minimum 95% *k*-mer completeness, 90% BUSCO single-copy completeness, QV45, 0.8 Mb haplotig N50, in addition to maximum 2% phase switch error rate and 2.5% false duplication rate. In the ICE-ONT/ILMN set, 636 haplotype assemblies (96.65%) passed all the quality control criteria and 22 assemblies (3.35%) failed at least one quality control requirement. In the ICE-PB set, 44 assemblies passed all quality control requirements and 54 passed all quality control requirements except the phase switch error rate, which cannot be computed without parental Illumina reads. Among the 17 individuals with retained ICE-PB and ICE-ONT/ILMN assemblies, variant calls from the ICE-PB assemblies of 16 individuals were merged and the remaining individual was set aside for validation purposes.

#### Small variants merging

We used the HPRC Y1 Minigraph-Cactus v1.1 VCF file, which contains variants converted to VCF format from the sequence graph produced by the Minigraph-Cactus pipeline for the haploid T2T-CHM13 assembly and the HPRC dual assemblies with many variants nested in a snarl. To obtain non-overlapping sites, bubbles were popped with vcfbub^58^ and only sites with alleles shorter than 100 kb were initially kept. All structural variants and variants with a star allele were then removed, followed by a left-alignment and normalization of all remaining variants. Diploid genotypes were then split into haploid genotypes. The HPRC haploid variant calls were merged with their ICE haploid counterparts using bcftools^59^. The resulting multi-sample VCF contains 787 haplotype samples: 602 samples from the ICE-ONT/ILMN haplotype assemblies, 96 samples from the ICE-PB haplotype assemblies, 88 samples from the HPRC haplotype assemblies (HG002, HG005 and NA19240 were set aside for internal validation) and the haploid T2T-CHM13 assembly. Complex variants were decomposed into simpler primitives with vcfwave^60^ and duplicated primitives were merged.

### Icelandic DNA data

Whole-genome sequencing of 57,630 Icelanders followed standard Illumina TruSeq PCR-Free methodology using HiSeqX, NovaSeq and NovaSeqX machines. All the samples were sequenced with minimum 20× coverage. Illumina SNP chip-typing was performed on 182,338 Icelanders for long-range phasing^61^ and imputation, as described previously^62^. Among the Illumina-sequenced Icelanders, 312 were sequenced with ONT R9.4 long reads and 49 were sequenced with PacBio HiFi (Supplementary Methods 5).

All participating subjects signed informed consent. The personal identities of the participants and biological samples were encrypted by a third-party system approved and monitored by the Data Protection Authority. The National Bioethics Committee and the Data Protection Authority in Iceland approved these studies.

### Statistical analyses

We used logistic regression with an additive model to test for the association between sequence variants and binary traits. The reported *P* values are based on two-sided tests with age and sex as covariates. No statistical methods were used to predetermine sample size for association testing. Reported correlations are Pearson’s correlation coefficients (*r*).

In Figs. 2 and 4, the box plots show the distributions of data points with the interquartile range (IQR) from the 25th to the 75th percentiles represented as a box, the median value represented as a line within the box, the whiskers as lines extending the box to the minimum and maximum values within 1.5 times of the IQR and the outlier values as data points extending beyond the whiskers.

### Reporting summary

Further information on research design is available in the Nature Portfolio Reporting Summary linked to this article.

## Online content

Any methods, additional references, Nature Portfolio reporting summaries, source data, extended data, supplementary information, acknowledgements, peer review information; details of author contributions and competing interests; and statements of data and code availability are available at https://doi.org/10.1038/s41586-026-10924-7.

## Supplementary information

Supplementary Information Supplementary Methods 1–15, including Supplementary Figs. 1–18 and Supplementary Tables 1–5. The supplementary sections, figures and tables provide additional methodological details, benchmarking results, validation analyses and supporting data for the findings presented in the main manuscript.

Reporting Summary

Peer Review File

## Figures and Tables

**Extended Data Table 1:**
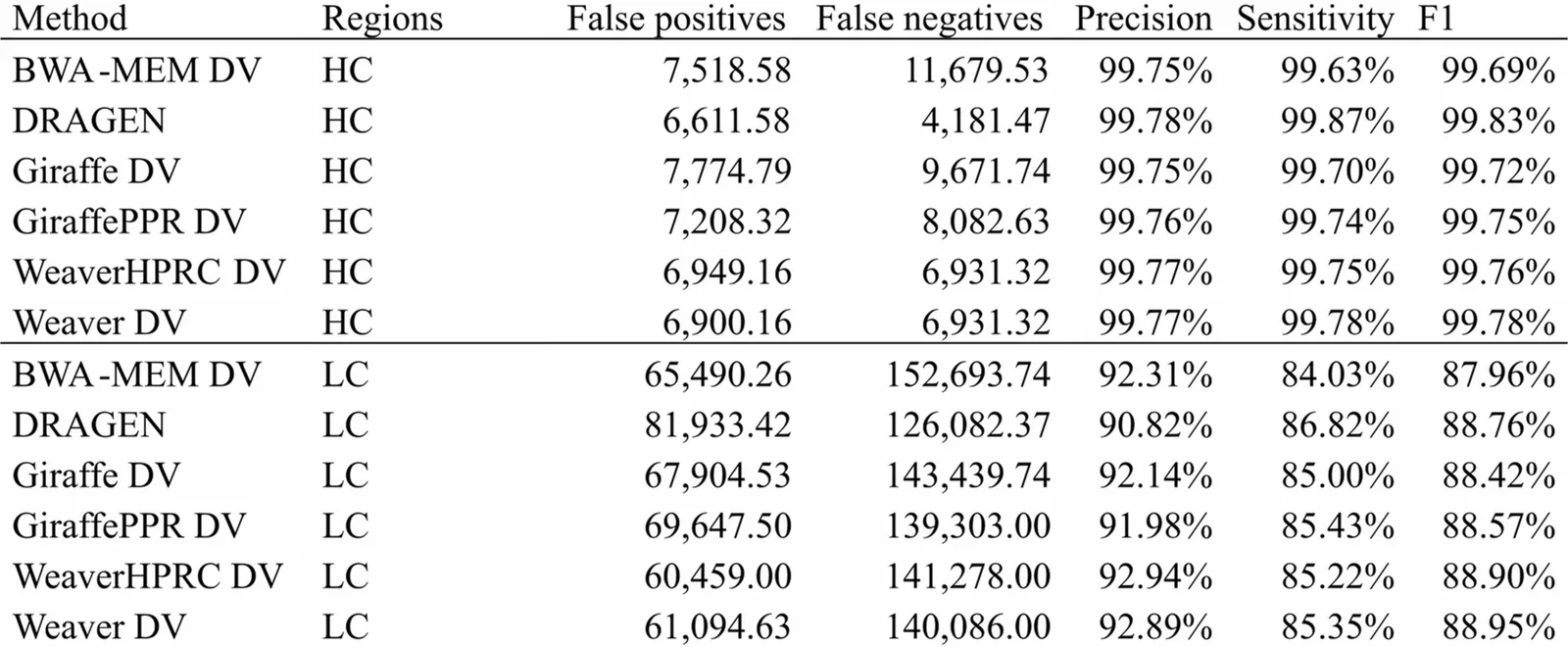
Comparison of different variant calling pipelines for 19 Icelanders

The truth sets used are safe variants called from haplotype-resolved dual assemblies made from PacBio HiFi and parental Illumina reads. High confidence (HC) regions are defined as the intersection of the truth set BED files and all seven GIAB small variants high confidence BED files. Regions outside of High Confidence regions are Low Confidence (LC). Reported values are the mean across the 19 Icelandic samples. PPR: Personalized pangenome reference. DV: DeepVariant.

## Data Availability

HPRC-ICE pangenome reference (GFA, VCF and the Weaver index) is accessible at https://doi.org/10.5281/zenodo.19609698 (ref. ^63^). Singularity containers for Weaver, Emblask and DeepVariant for using the weaver trained model are accessible at https://doi.org/10.5281/zenodo.21240459 (ref. ^64^). Access to the raw Icelandic sequence data cannot be made publicly available because Icelandic law and the regulations of the Icelandic Data Protection Authority prohibit the release of individual-level and personally identifying data. Data access can be granted only at the facilities of deCODE genetics in Iceland, subject to Icelandic law regarding data usage. Anyone wishing to gain access to the data should contact D.F.G. (dgudbjar@amgen.com). Icelandic law allows for unimpeded sharing of summary-level data. Other accessed data: The human reference genome GRCh38/hg38 was obtained from the Genome Reference Consortium/NCBI Assembly database, RefSeq accession GCF_000001405.26 and GenBank accession GCA_000001405.15. HPRC graph: https://github.com/human-pangenomics/hpp_pangenome_resources. GA4GH/T2T/GIAB stratification regions: https://ftp-trace.ncbi.nlm.nih.gov/ReferenceSamples/giab/release/genome-stratifications/v3.3/GRCh38@all/. GIAB T2T-HG002 Q100 small variant benchmark: https://ftp-trace.ncbi.nlm.nih.gov/ReferenceSamples/giab/data/AshkenazimTrio/analysis/NIST_HG002_DraftBenchmark_defrabbV0.018-20240716/. GIAB HG002 short reads: https://ftp-trace.ncbi.nlm.nih.gov/ReferenceSamples/giab/data/AshkenazimTrio/HG002_NA24385_son/NIST_HiSeq_HG002_Homogeneity-10953946/NHGRI_Illumina300X_AJtrio_novoalign_bams/HG002.GRCh38.60x.1.bam. GIAB HG003 short reads: https://ftp-trace.ncbi.nlm.nih.gov/ReferenceSamples/giab/data/AshkenazimTrio/HG003_NA24149_father/NIST_HiSeq_HG003_Homogeneity-12389378/NHGRI_Illumina300X_AJtrio_novoalign_bams//HG003.GRCh38.60x.1.bam. GIAB HG004 short reads: https://ftp-trace.ncbi.nlm.nih.gov/ReferenceSamples/giab/data/AshkenazimTrio/HG004_NA24143_mother/NIST_HiSeq_HG004_Homogeneity-14572558/NHGRI_Illumina300X_AJtrio_novoalign_bams/HG004.GRCh38.60x.1.bam. CARD HG002 ONT R9.4 assembly: https://s3-us-west-2.amazonaws.com/human-pangenomics/index.html?prefix=publications/Napu_paper_ONT_Coriell_SingleFC_2023/HG002_R9/assembly/. HPRC assemblies: https://github.com/human-pangenomics/HPP_Year1_Assemblies. HPRC GRCh38 Minigraph-Cactus: https://s3-us-west-2.amazonaws.com/human-pangenomics/index.html?prefix=pangenomes/freeze/freeze1/minigraph-cactus/. Clinvar variants (VCF): https://ftp.ncbi.nlm.nih.gov/pub/clinvar/vcf_GRCh38/archive_2.0/2025/clinvar_20250209.vcf.gz. Ensembl genes (v112): https://ftp.ensembl.org/pub/release-112/gtf/homo_sapiens/Homo_sapiens.GRCh38.112.gtf.gz.

## Code availability

The Weaver source code is available at github.com/DecodeGenetics/weaver (https://doi.org/10.5281/zenodo.21235339 (ref. ^65^)) and its Nextflow workflow (Supplementary Methods 9) is available at github.com/DecodeGenetics/nf-weaver (https://doi.org/10.5281/zenodo.21223186 (ref. ^66^)). The Emblask workflow is available at github.com/DecodeGenetics/Emblask (https://doi.org/10.5281/zenodo.21223317 (ref. ^67^)).
