# Supplementary material for: An Icelandic pangenome reference
Source: Nature. 2026 Aug 19;658(8135):421–9. doi: 10.1038/s41586-026-10924-7 (PMC13645673; doi:10.1038/s41586-026-10924-7)
Supplement: Supplementary file 1 [file 41586_2026_10924_MOESM1_ESM.pdf]

nature portfolio

https://doi.org/10.1038/s41586-026-10924-7

Supplementary information

An Icelandic pangenome reference

In the format provided by the authors and unedited

An Icelandic pangenome reference
Supplementary materials

Guillaume Holley*, Hannes P Eggertsson*, et al.

July 15, 2026

* These two authors contributed equally to this work.

1 Supplementary Methods 1: Pangenome reference graph model

The pangenome reference graph used in this study is a sequence graph generated from haplotype assemblies. It is stored in two data files: a graph in reference Graphical Fragment Assembly (rGFA) format that contains the SVs indexed with a reference coordinate system and a phased Variant Call Format (VCF) file that contains the small variations found in the assemblies. The rGFA format extends the GFA format with additional mandatory fields that provide a unique and stable coordinate system across the graph.

For coordination and determining from which assembly a sequence originates from, the rGFA format requires segments to be tagged with the original assembly Sequence Name (SN) and Sequence Offset (SO). Additionally, both segments and links contain a tag with the Sequence Rank (SR) which represent the assembly insertion order index of when that sequence was first inserted in the graph with respect to the other assemblies. The reference assembly has rank 0 while the non-reference assemblies have ranks > 0. Therefore, traversing paths with the same rank spells out a stable sequence. These sequences are denoted stable because they can always be spelled out from the graph after new assemblies have been inserted. The reference assembly sequence, which can be reconstructed by traversing all paths of rank 0, is of particular importance as all output alignments are represented with respect to it.

The rGFA-formatted graph contains the large variants of the non-reference haplotypes with respect to the reference assembly. The small variants are not included in the construction of the rGFA because including every variant in the rGFA would greatly increase the structural complexity and size of the graph. Instead, a VCF file is used to complement the rGFA file to define smaller variations such as SNVs and indels. Other methods [14, 45] have used VCFs with reference genomes in FASTA format to represent variations in graphs but, to our knowledge, Weaver is the first method supporting variations defined collectively in both rGFA and VCF files.

Segments are defined by S-lines in the rGFA, while pairs of segments are connected with links defined by L-lines. A link is made of two edges connecting the vertices of the two segments in each direction. Hence, a reverse edge connects a pair of vertices in the opposite direction with respect to the forward edge. A path in the graph is defined as a sequence of segments connected with links. A path in the graph following segments and links of the same rank spells out a stable sequence.

2 Supplementary Methods 2: Weaver

2.1 Weaver indexing details

The Weaver index is comprised of two hash tables, one for the minimizer index and the other for an ICU index. The minimizer index associates minimizers with their locations in the graph. A minimizer is a k-mer with the smallest hash value in a fixed-length window containing w k-mers. By default, Weaver uses $k = 23$ and $w = 11$.

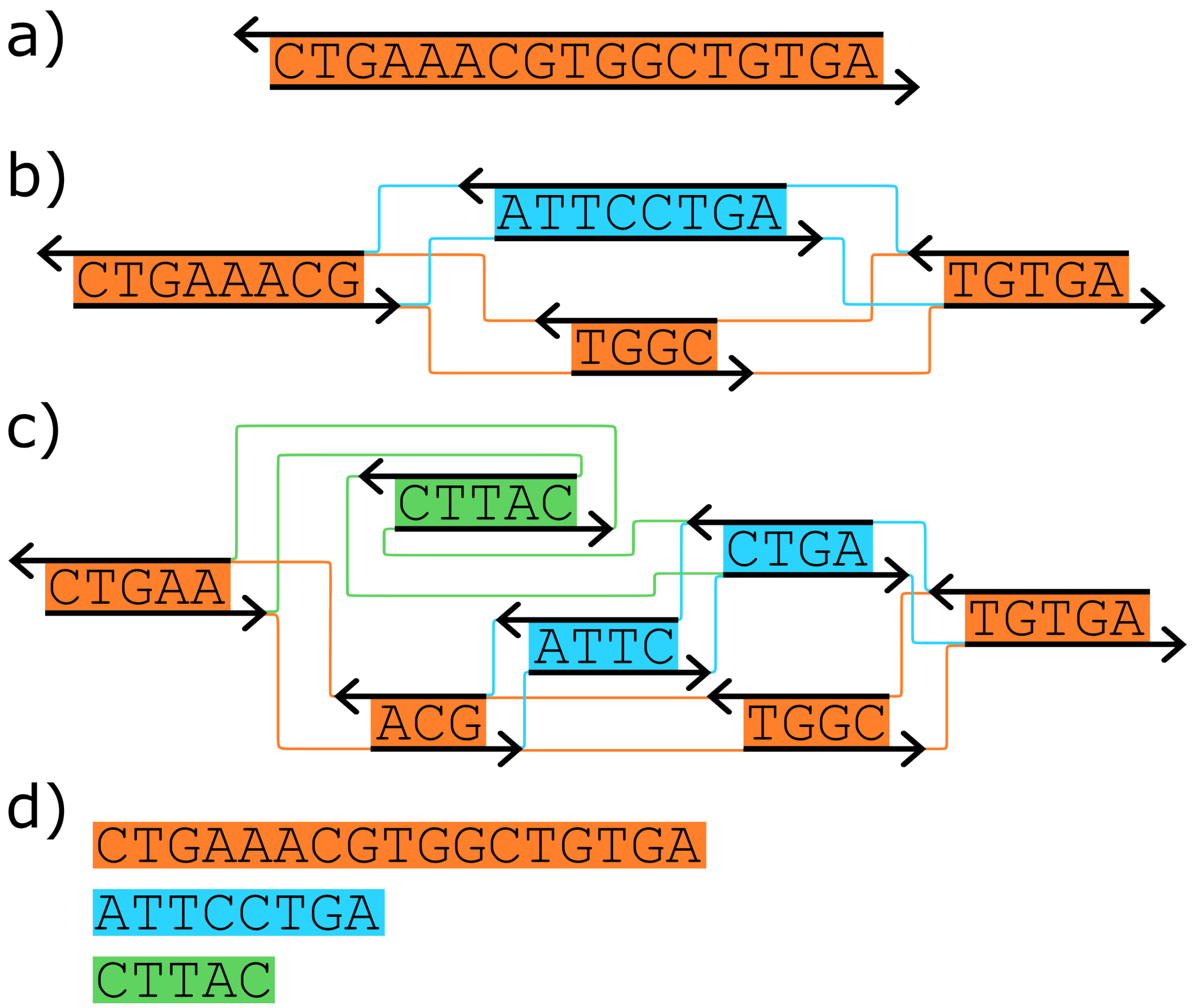

Supplementary Figure S1: Incremental construction of an rGFA graph. a) The graph is composed of a single segment containing the full sequence of the first added haplotype assembly. The segment is made of two vertices with opposite directions. The segment color indicates the sequence origin and rank. b) A second haplotype sequence is added to the graph, resulting in the splitting of the orange segment into three segments and the insertion of a new segment with a different color and rank. Edges connect pairs of vertices along a path. The first added haplotype sequence can be reconstructed by traversing the orange path in the graph. c) Adding a third haplotype assembly requires splitting up two segments of the previous paths without modifying the sequence content. d) Walking the orange, blue and green paths yields the stable sequences.

The minimizers are found by sliding a window of size $w+k-1$ along each haplotype of the graph and storing in a hash table the locations of k-mers that have the smallest hash value among all the k-mers of each window (Figure S2). The hash function we use computes the same hash value for a k-mer and its reverse complement and thus the resulting minimizers are the same regardless of the orientation of the sliding window. As opposed to storing every k-mer of the graph, the motivation of storing minimizers is to systematically subsample k-mers of the graph to reduce the size of the index and lower memory footprint. With the minimizer index, we can query the minimizers of the read sequences to retrieve potential mapping locations in the graph.

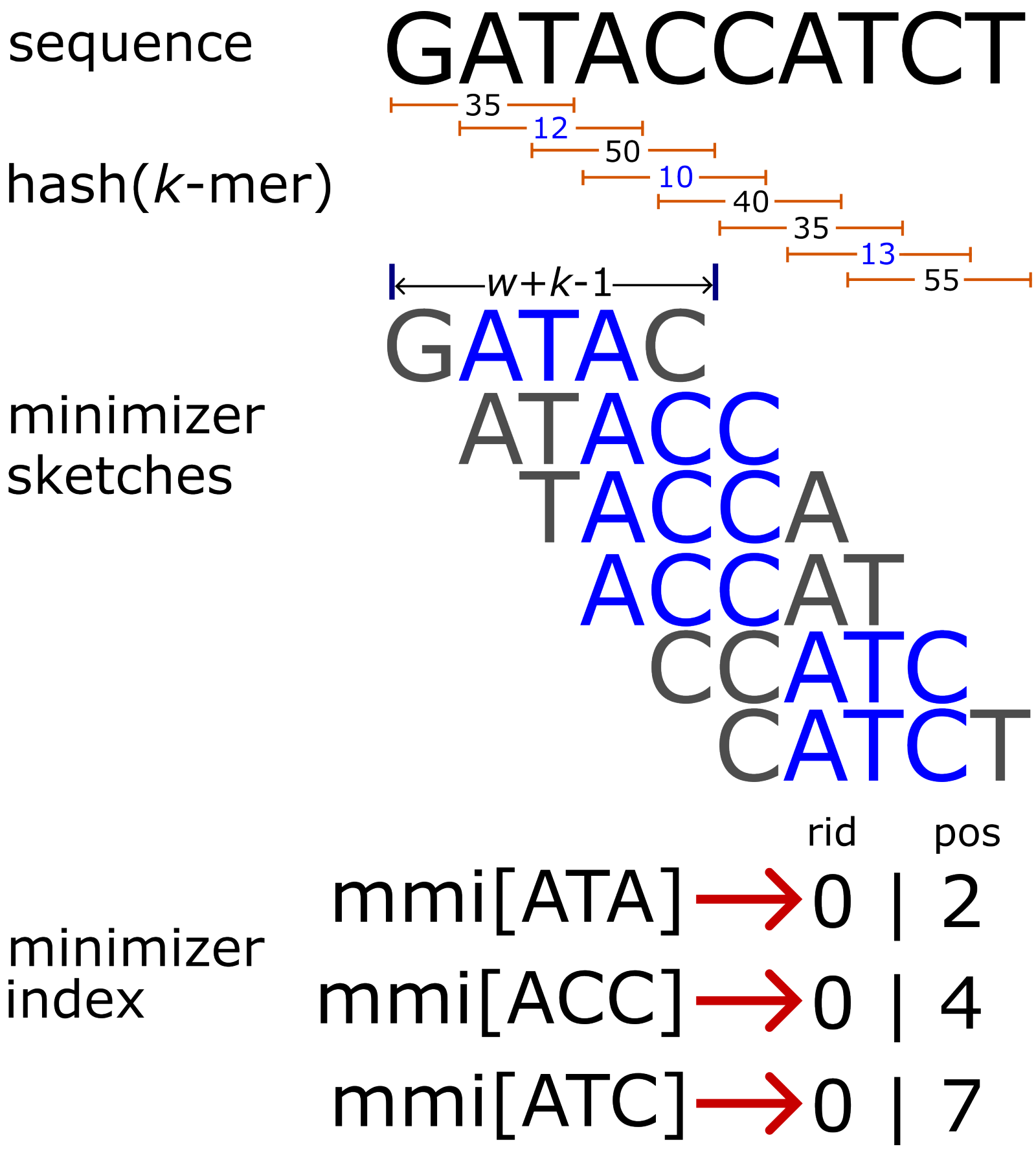

Supplementary Figure S2: Construction of the Weaver minimizer index with three 3-mers in a window. Initially, the window spans the subsequences `GAT`, `ATA` and `TAC` with hash values 34, 12 and 50, respectively. In that window, the 3-mer `ATA` and its position are stored in the hash table because its hash value is the smallest in the window. As we start to slide the window, we find the 3-mer `ACC` with hash value 10, which is smaller than all other hash values of the window so it is also stored. We slide the window further without finding a hash value smaller than 10 but once 10 is no longer in the window, we recheck all hash values for a new minimum which is 13 in this example.

We represent the location of a minimizer with a single 64 bit value. It consists of a segment ID (32 bits), its position within that segment (31 bits) and the orientation compared to the segment sequence (1 bit). Weaver requires k to be an odd number such that a base is at its center position. The indexed minimizer position is that center position such that if the minimizer spans multiple segments, its center determines its segment. The hash function used in Weaver yields the same hash value for a k-mer and its reverse complement. That way, it is enough to index segments of the graph in forward direction only, as the reverse direction would yield the same minimizer hash values in reverse order.

The ICU index keeps track of nearby vertices in the graph. A vertex u "sees" vertex v if there exists a path from u to v in the graph which is no more than d bps long ($d = 1500$ by default). Since all edges of the graph are bidirectional, if u sees v then the reverse vertices of u and v also see each other. Every such vertex pair of the graph is stored in the ICU index as well as the shortest distance between the two vertices of the pair. To create the ICU index, we simply start from each

vertex $u \in G$ a depth-first traversal of all possible paths shorter than d bps.

The motivation of having the ICU index is to quickly query whether two minimizers of a read are near each other in the graph. If both minimizers are located are the same segment in the graph, we trivially know their distance in the graph sequence. However, if they are on different segments, we use the ICU index to check whether the shortest path between them is longer than their distance in the read.

Finally, Weaver also stores all VCF records in the index structure. This includes the chromosomal position, alleles and phased genotypes of each haplotype. These are used at a later step when scoring the read alignments.

2.2 Weaver mapping

Weaver read mapping follows the seed-and-extend paradigm. In summary, the seeds are minimizer locations that are short exact matches found in the minimizer index. They are to be extended to generate a complete read alignment. Two heuristic steps, bucketing and chaining, take place between seeding and extending.

The first step is to find all the minimizers in the read sequence, i.e. $\{m_0, m_1, ...\} \in M$, using the same k and w parameters as used by the minimizer index. Those minimizers are queried in the minimizers index to find all of their exact matches and associated locations in the graph. Any minimizer with no match is immediately discarded and thus, each minimizer $m_i \in M$ is associated with one or more minimizer locations, $l_{i,j} \in L_i$. As described in Section 2.1, a minimizer location is composed of a segment ID, a position and a strand orientation.

All minimizer locations are assigned to buckets by bit-shifting their 64 bits location by 10 bits towards the least significant bit, thus discarding the 10 least significant bits. As a result, buckets do not contain more than 1024 minimizer locations ($2^{10} = 1024$) and each bucket tends to cluster minimizers located on the same segment.

A bucket b has size s_b calculated from the number of bits in the bucket and weighted with the number of minimizer locations, $|L_i|$.

$$s_b = \sum_{m \in M} \sum_{l_{i,j} \in L_i} \max\left(1, \text{clz32uint}\left(|L_i|\right) - 20\right) \text{ if } l_{i,j} \text{ is in b and 0 otherwise} \tag{1}$$

where clz32uint returns the count of leading zeroes in a 32 bits unsigned integer. This way, minimizers that have many locations will each contribute less than those with fewer locations. Once the bucket sizes are computed, we find the largest 24 buckets (by default) for each read mate of a pair and then do the same with bucket sizes with both mates at once. We then discard any minimizer location that is not in those buckets or adjacent to them.

The next step is connecting the found minimizers to form chains. We define a chain as a list of minimizers, $m_1, ..., m_n$ that continuously match both the read sequence and the graph sequence. The i-th minimizer m_i of a read has its center position in the read sequence at position p_i and minimizer locations $l_{i,j}$ in the graph sequence. We process the minimizers in ascending order of their read positions. The locations of the minimizer with the smallest read position L_1 form the initial chains. Then, for each subsequent minimizer locations, L_2, we check whether there is a walk in the graph with a distance between the two locations that is the same as in the read, i.e. $d = p_2 - p_1$. In that case, we extend the chain. Such walks of distance d are fast in practice as $d \leq l - k - w$ where l is the short read length. Furthermore, the walk can be immediately stopped when the segments at the minimizer locations have a distance between them larger than d in the ICU index.

Additionally, if there is a chain for which no location of the next minimizer can be extended, the search is repeated with the exception that the allowed distance in the interval is

$$\left[p_2 - p_1 - \left(1 + \frac{p_2 - p_1}{6}\right), p_2 - p_1 + \left(1 + \frac{p_2 - p_1}{6}\right)\right] \tag{2}$$

If the location can still not extend a previous chain, a new chain is formed containing only that location. This same process continues in a similar manner for the subsequent minimizer

locations and then for the other mate of the paired-end read. Each end of the read outside of the chained parts are next aligned to the graph sequence using a pairwise extension alignment algorithm from the paw.Align library (`https://github.com/hannespetur/paw`). Combining the seed and extensions alignments results in a full alignment between the read sequence and the graph sequence.

Alignments are scored to determine which of them is the most likely to be the correct one. The haplotype score of haplotype h is defined as

$$S_h = A \ \texttt{\#matches} - B \ \texttt{\#mismatches} - C \ \texttt{\#clips} - O \ \texttt{\#gapOpen} - E \ \texttt{\#gapExtend} \quad (3)$$

where $A = 1, B = 4, C = 6, O = 7, E = 1$ by default. In S_h, `#matches` is the number of bases that matched, `#mismatches` is the number of bases that did not match and `#clips` is the number of times the extension alignment did not fully align so it was clipped. Gaps are insertions and deletions in the alignment. They have an affine cost composed of `#gapOpen` which is the number of indels in the alignment and `#gapExtend` which is the number of times a base was inserted or deleted after the first one. The final read alignment is the one maximizing S_h with h as the reference with all gaps as far left as possible.

The variations and genotypes of the haplotypes $h \in H$ are obtained from the VCF associated to the input graph. A linear reference genome mapper would calculate its alignment score based on only one of the haplotypes, the reference, which causes a bias towards that allele. A Weaver alignment score S is calculated as the weighted average haplotype score of all of the haplotypes present in the VCF (Figure S3).

$$S = \sum_{h \in H} P(h|S_h) S_h \quad (4)$$

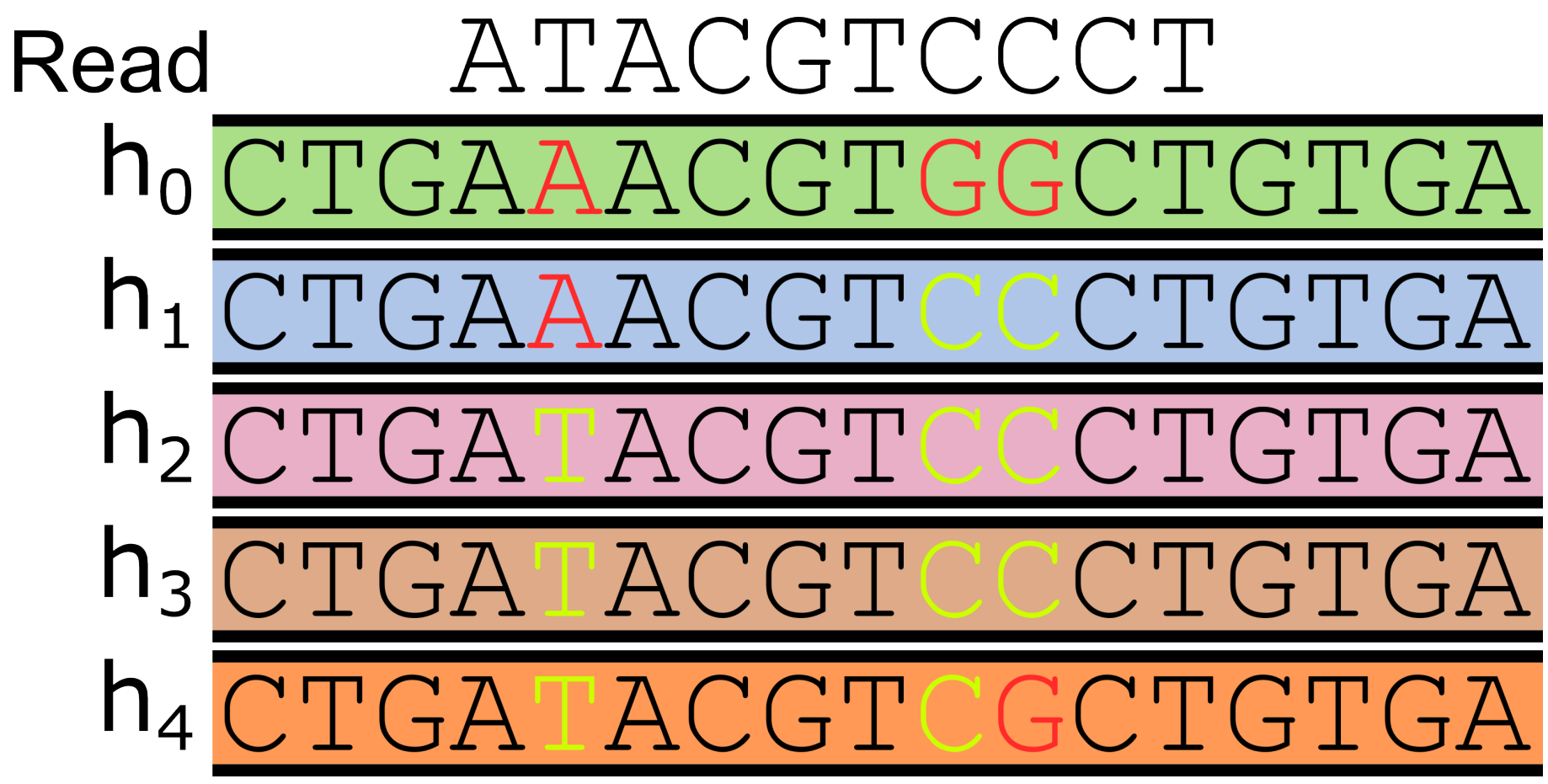

Supplementary Figure S3: Weaver alignment score calculation. Bases at polymorphic sites are colored green if they match the read sequence and red if not. A haplotype alignment score is calculated for each haplotype by pairwise comparing the read sequence and the haplotype sequence. The final alignment score is the weighted average of all haplotype alignment scores, weighted by the probability that the read originated from that haplotype.

where $P(h|S_h)$ is the probability that the read was sequenced from haplotype h. In multiple prior methods [14, 45, 2], the alignment score is simply the single highest scoring haplotype alignment. This is problematic as the presence or absence of a single haplotype can drastically affect the alignment score. In contrast, every haplotype contributes to the alignment score in Weaver.

We estimate the probability that the sequence read was originally sequenced from haplotype h by interpreting the haplotype alignment scores as log-likelihoods with

$$P(h|S_h) = \frac{\exp(\lambda_h S_h)}{\sum_{h \in H} \exp(\lambda_h S_h)} \quad (5)$$

where λ_h is a normalization factor. The Weaver alignment score S is rounded to the nearest integer and reported in the `WS` SAM tag while the alignment score with respect to only the reference

allele is reported in the standard `AS` SAM tag.

Mapping qualities are posterior probabilities of a mapping error and are customarily represented on a PHRED scale. We estimate mapping qualities similarly by interpreting the alignment scores as log-likelihoods of a Viterbi path through a pair of hidden Markov model [45, 44], similar to the way haplotype alignment scores are intepreted above. The probability of an alignment $\hat{a}$ to be correctly mapped between query Q and graph G among all alignments $a \in A$ is formulated as

$$P(\hat{a}|Q, G) = \frac{\exp(\lambda_2 S(\hat{a}))}{\sum_{a \in A} \exp(\lambda_2 S(a))} \tag{6}$$

where λ_2 is a normalization factor. In the set of alignments A, we include alignments that we extended and one alignment with an alignment score of 0 to represent all other possible alignments we did not explore. We give alignments with multiple equal mapping quality of zero. Additionally, we cap mapping qualities to 60 in order to account for the various heuristics we have set in place in Weaver.

2.3 Mapping workflow

A typical Weaver mapping workflow is as follows:

```
weaver index <rGFA> --vcf=<VCF>
weaver map <rGFA> <FASTQ|FASTQ.gz> | samtools markdup - <BAM/CRAM>
```

The default input is an interleaved FASTQ file but one can also map a pair of FASTQ files by specifying a second FASTQ with the `--fq2` option. The pangenome reference graph, composed of a pair of rGFA and VCF files as defined previously, is first indexed with the `weaver index` command. The indexing step only needs to be run once for a graph as the index is stored on disk.

The `weaver map` command then loads the index from disk, maps all input short reads file and writes a sorted SAM to the standard output. The mapped reads can be tagged with the specified read group using the `-R` optional argument, i.e. `-R "@RG\tID:foo\tSM:bar"`. Weaver writes the records in position sorted order and includes the necessary tags to mark duplicates. Thus, the output SAM data can be piped into `samtools markdup` which annotates duplicates and the final output is then written to a BAM/CRAM file. This BAM/CRAM file can then be used for variant calling or other analysis.

We provide a Nextflow workflow for indexing a graph and mapping with Weaver at: `github.com/DecodeGenetics/nf-weaver`. The workflow can also genotype with DeepVariant using a model trained on Weaver aligned data.

3 Supplementary Methods 3: Emblask

Emblask is a diploid genome assembly pipeline that produces a set of two haplotype assemblies, also called dual assembly, from parent-offspring trio data. The method is a hybrid approach using both noisy long reads and accurate short reads, namely ONT R9.4 and paired-end Illumina reads in this study. The pipeline takes advantage of state-of-the-art assembly and error correction tools to substantially decrease the error rate of the input long reads and assign them to their respective haplotypes.

Previous hybrid assembly efforts have mainly focused on microbial genomes such as bacteria and viruses [1, 52] with applications to metagenomics [3]. These methods often rely on the construction of an assembly graph from the short reads followed by a long reads assisted graph traversal to resolve repeats. Yet, the usage of hybrid assemblers for larger diploid and polyploid organisms such as mammals has been limited due to the additional complexity of combining reads from different sequencing technologies. Wengan [10] and MaSuRCA [54] are hybrid assemblers for large genomes using the previously described assembly paradigm but can only produce mixed-haplotype assemblies for non-haploid organisms. The HyPo assembler [9] produces haplotype-resolved dual assemblies by constructing a mixed-haplotype assembly from the long reads only and then polishes the produced contigs into phased diploid contigs with the short reads. Generally, short reads are frequently used for pre-assembly error correction of the long reads [50, 18] or post-assembly contig polishing [49, 48, 51]. One one hand, a recurrent issue with many hybrid methods for long read error correction is that they do not retain the phasing of the reads. On the other hand, while

assembly short read polishing efficiently fixes small errors, it cannot resolve larger structural issues such as duplicated or collapsed haplotype sequences caused by the high error rate of the input long reads. To address these issues, Emblask ties together the assembly, the correction and the polishing steps by:

- progressively correcting the input long reads with the short reads during assembly
- assigning each haplotype-resolved assembled sequence to the paternal or maternal haplotype
- polishing each haplotype assembly with the short and long reads

An overview of the Emblask assembly pipeline is given in Figure S4.

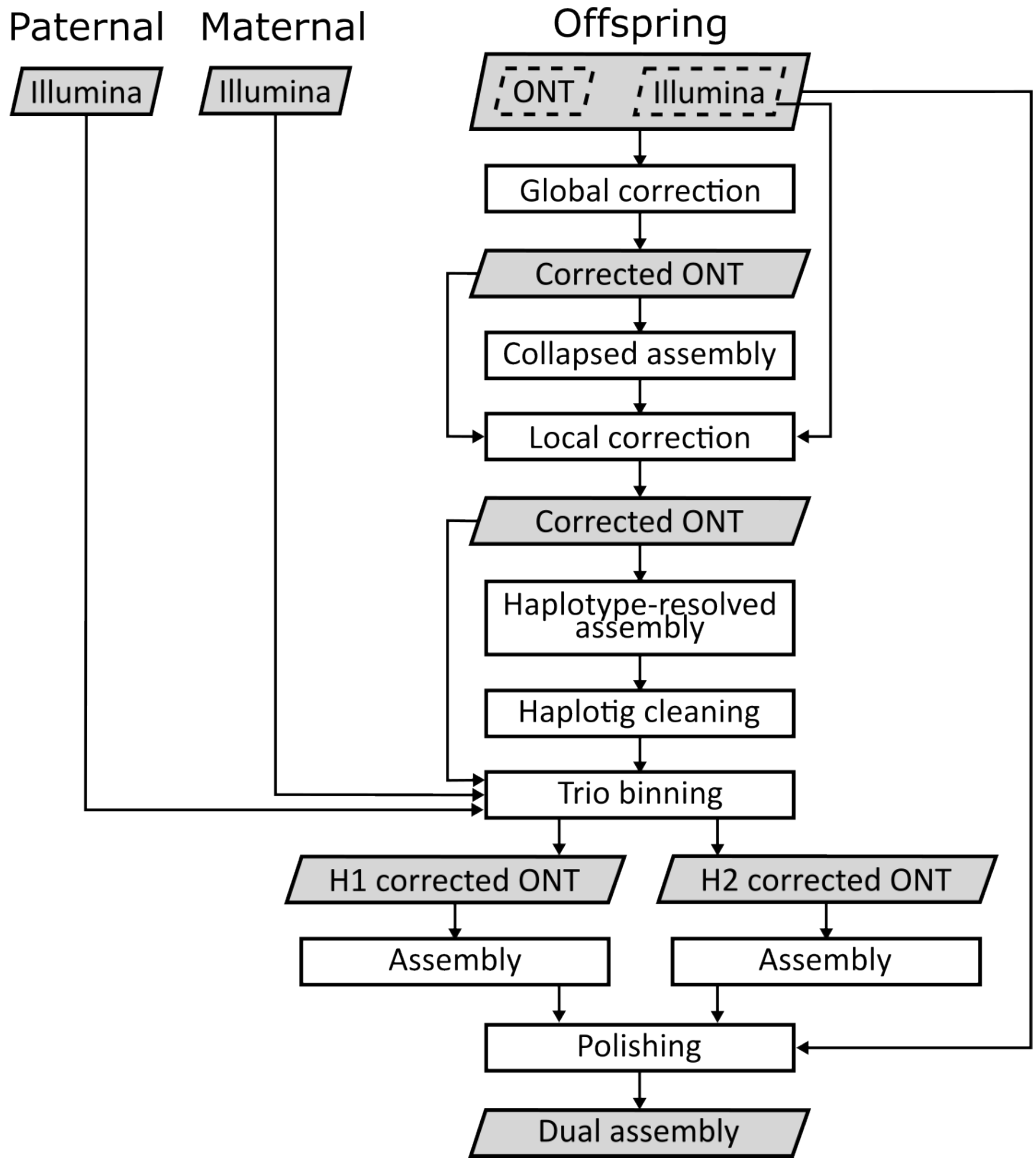

Supplementary Figure S4: Flowchart of the Emblask assembly pipeline. Data are represented in grey background parallelograms while operations are shown in white background rectangles.

In the following, we refer to long reads as LR and short reads as SR. Coverage always refers to the mean LR coverage depth unless specified otherwise. We define $\text{cov}(\mathcal{A})$ and $\text{cov}(\mathcal{A}, s)$ as the coverage of assembly $\mathcal{A}$ and the coverage of sequence $s \in \mathcal{A}$ respectively.

3.1 Global error correction

The first step of the Emblask pipeline is to decrease the LR error rate with Ratatosk [18]. Ratatosk is a hybrid LR error correction tool based on the compacted and colored de Bruijn graph. At first, a compacted de Bruijn graph is built from the SRs and the k-mers of the graph are colored by the identity of the SRs in which they occur. A color highlights a path in the graph that an SR traverses. After anchoring an LR sequence on the vertices of the graph, the LR k-mers which are not present in the graph delineate the erroneous sub-sequences to correct. An erroneous sub-sequence is enclosed with anchoring k-mers and is corrected by traversing the graph from the leftmost to the rightmost

anchoring k-mer. The graph traversal is guided by the sequence similarity of the traversed path to the sub-sequence to correct as well as traversing vertices which share colors with the adjacent anchoring k-mers. Ratatosk corrects the LRs in two stages. In the first stage, the graph is built from and colored by the SRs. This stage resolves most of the errors but erroneous sub-sequences longer than the insert size of paired-end SRs as well as erroneous regions spanning very tangled sub-graphs might be left uncorrected. In a second stage, the graph is rebuilt from the SRs using a longer k-mer length in order to generate a more contiguous graph. Furthermore, the vertices of the graph are colored with the LR sub-sequences corrected during the first stage to highlight paths in the graph which are longer than the SR paths of the first stage. After correction, Emblask removes from the corrected LRs all windows longer than 100 bps for which at least 90% of the bases have a low correction score reflecting an uncorrected or low-quality corrected sub-sequence. Reads are therefore split by removing the low-quality sub-sequences and only split reads longer than 5 kbp are kept for further processing.

3.2 Collapsed assembly

The previously corrected LRs are assembled into a collapsed assembly $\mathcal{A}_c$ with Flye [25] such that the assembled sequences, called *contigs*, contain alleles from the paternal and maternal haplotypes. Occasionally, the assembler produces haplotype-resolved contigs called *haplotigs* (Figure S5). To filter out haplotigs from $\mathcal{A}_c$, the LRs are mapped back to the contigs of $\mathcal{A}_c$ with minimap2 [28] and any contig c with $\text{cov}(\mathcal{A}_c, c) < 0.6 \cdot \text{cov}(\mathcal{A}_c)$ is flagged as a haplotig candidate. Small variants are called and phased on the haplotig candidates with PEPPER-Margin-DeepVariant (PMDV) [40] using a model trained for Ratatosk-corrected ONT R9.4 reads. PMDV splits the phased variants into phase sets, each one having enough LRs spanning all its heterozygous variants to confidently phase these variants. However, the phasing of variants across adjacent phase sets is unknown because phase sets are interspersed by long stretches of homozygosity or insufficient coverage preventing further phasing. LR alignments are then *haplotagged* by assigning them to a phase set and a haplotype H_1 or H_2 within that phase set, depending on their overlap to the alleles of the phased variants. While phasing variants and haplotagging alignments are always preceded by LR mapping and small variant calling, these two initial steps will not be explicitly mentioned in the following for simplicity. Once variants are phased and alignments are haplotagged with respect to $\mathcal{A}_c$, a haplotig candidate is filtered out if it has less than 2 heterozygous variants or if it contains a single phase set with an unbalanced coverage (one haplotype in the phase set represents at least 80% of the phased LR coverage).

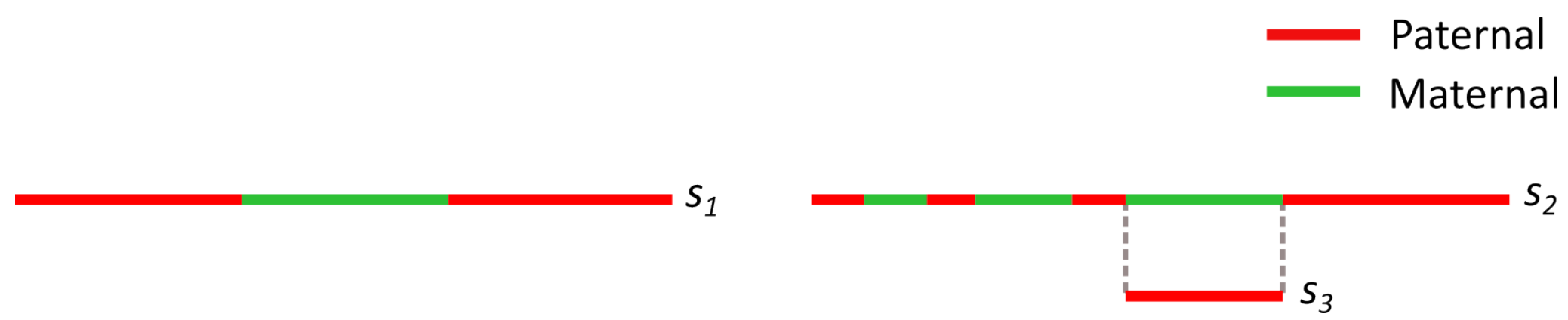

Supplementary Figure S5: Collapsed assembly with 3 assembled sequences. Contigs s_1 and s_2 are composed of alleles from the paternal and maternal haplotypes. Sequence s_3 is fully haplotype-resolved.

3.3 Local error correction

This first correction step in the Emblask pipeline described in Section 3.1 is said to be "global", all the LRs are corrected at once using a single graph built from all the SRs, as opposed to a "local" targeted correction in which some of the input reads aligned to a reference sequence are selected for correction. In order to further decrease the error rate of the globally-corrected LRs, a targeted error correction step is performed by decomposing $\mathcal{A}_c$ into windows and only correcting together the LRs mapping in each window (Figure S6). Iteratively correcting reads within windows enables to generate smaller and more contiguous graphs than for a single global correction which in turn leads to additional error correction. To this end, SRs and corrected LRs are mapped to the contigs of $\mathcal{A}_c$. Non-overlapping segments of 200 kbps are defined on $\mathcal{A}_c$ and used to split the read alignments into different windows that will be processed separately. Note that a paired-end SR can occur in multiple windows if it cannot be mapped accurately to a single position on $\mathcal{A}_c$. The coverage of each window is then computed and a region within a window is defined as high coverage

if its coverage exceeds the window coverage by 40% and its length is at least 3 times the N50 of the LRs in that window. Subsequently, each window is processed independently by extracting all of its reads and correcting separately the LRs for high and non-high coverage regions. A window correction entails two Ratatosk rounds with increasing k-mer sizes followed by one self-correction Ratatosk run that uses exclusively the previously corrected LRs to build and color the graph. In the following, LR refers to the globally and locally corrected LRs.

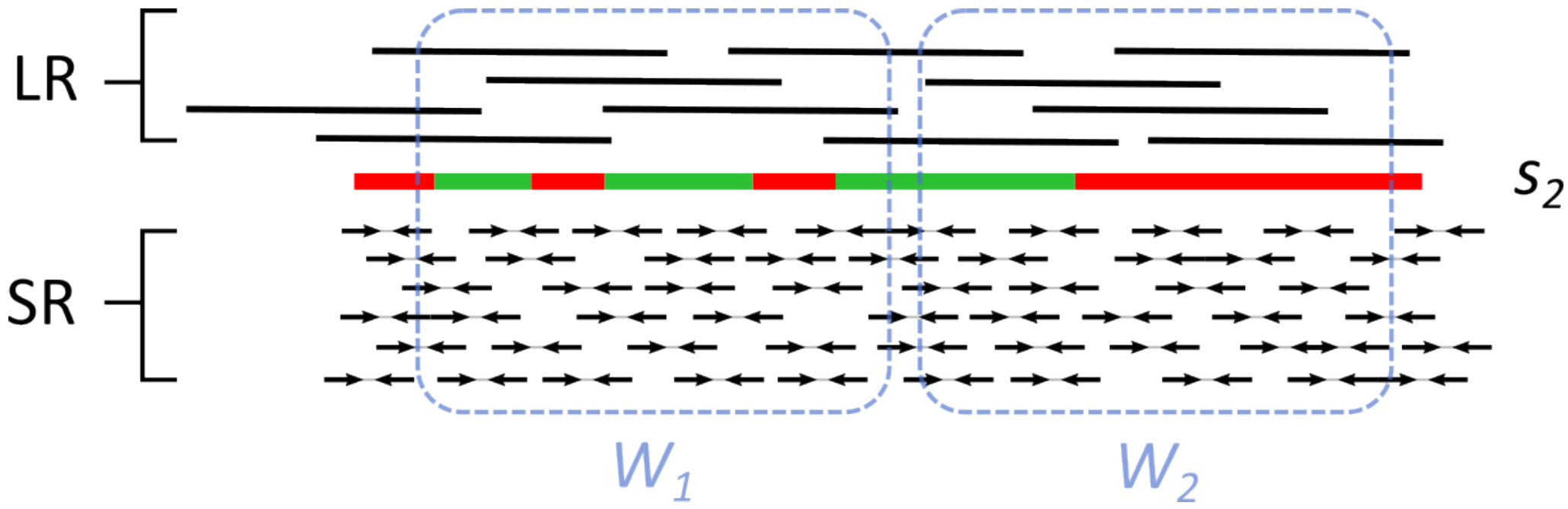

Supplementary Figure S6: LR and SR alignments with respect to a contig of the assembly. The alignments are split in two windows w_1 and w_2. All the LRs in each window are corrected using all the SRs of the same window.

3.4 Haplotype-resolved assembly

The LRs are assembled into a haplotype-resolved assembly $\mathcal{A}_r$ with Flye. The output assembly is composed of haplotigs representing sub-sequences of the paternal or maternal haplotype but without distinction to which of the two haplotypes each sequence is from. However, $\mathcal{A}_r$ is fragmented because it is common occurrence that only one of the two parental haplotypes gets assembled for any given locus. Inversely, the assembler can generate more haplotigs than there are alleles for a locus because there exist enough supporting LRs for which either the error rate remains too high even after correction or the phasing has been disrupted by the correction. Such spurious haplotigs often have a low coverage and can trivially be removed by requiring a minimum coverage, set to 5 by default in Emblask, for each haplotig.

3.5 Coarsed-grained haplotig cleaning

To create the final dual assembly, each haplotig must be assigned to either the paternal or maternal haplotype. Errors in the haplotigs can lead to an incorrect haplotype assignment which would result in fragmentation, gaps and false duplications in the final assembly. Therefore, haplotigs must be filtered, split and polished before performing the haplotype assignment. To this end, LRs are mapped to the haplotigs of $\mathcal{A}_r$ and alignments with low-mapping quality, high error rate or short alignment lengths are filtered out. Using the remaining alignments, haplotigs are then further filtered based on their coverage. The coverage of each haplotype assembly is expected to be half the coverage of the haplotigs in $\mathcal{A}_c$, i.e. $c = \frac{\text{cov}(\mathcal{A}_c)}{2}$. Let h_r and h_a be respectively the reference and alternate haplotigs for a given locus. On one hand, if h_r and h_a have been assembled and one of the two is erroneously duplicated, e.g. there is a haplotig similar or identical to h_r in $\mathcal{A}_r$, the coverage of each duplicated haplotig will be less than half of c. On the other hand, if only one of h_r and h_a has been assembled and that haplotig erroneously collapses variants from another locus, its coverage will be at least $3c$. An example of erroneous haplotig duplication and collapsing is illustrated in Figure S7.

Hence, only haplotigs h' with coverage within the range $\frac{c}{2} < \text{cov}(\mathcal{A}_r, h') < 3c$ are kept. Some of the kept haplotigs have a higher coverage than c due to either missing the corresponding alternate sequences in the assembly or having alleles from different haplotypes collapsed into a single sequence. In the latter case, mixed-haplotype sequences must be split into haplotype-resolved sub-haplotigs as the polishing step requires variants to be properly phased on the haplotigs. For this purpose, variants are called and LR alignments are haplotagged with respect to $\mathcal{A}_r$. Using the alignments, all the kept haplotigs with coverage at least 50% larger than c are annotated as collapsed coverage (CC) candidates. Haplotigs that are CC candidates are suspected of not being haplotype-resolved but could also be valid haplotigs for which the alternate sequence for the same loci is not present in the assembly. First, CC candidates with multiple phase sets are split between

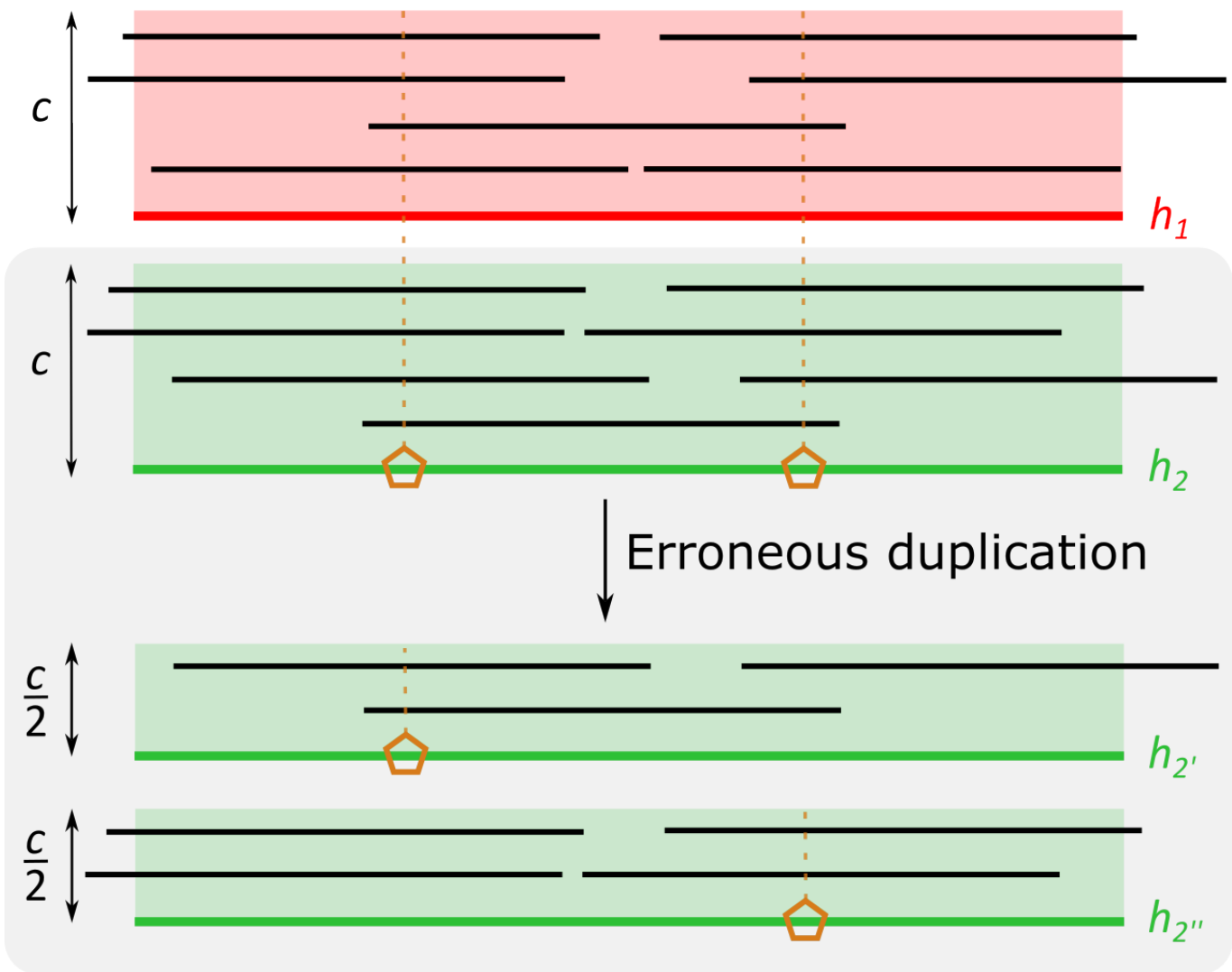

(a) Reference and alternate haplotigs h_1 and h_2. Haplotig h_2 is erroneously duplicated during assembly, resulting in two haplotigs h_2' and h_2'' with coverage $\frac{c}{2}$ each.

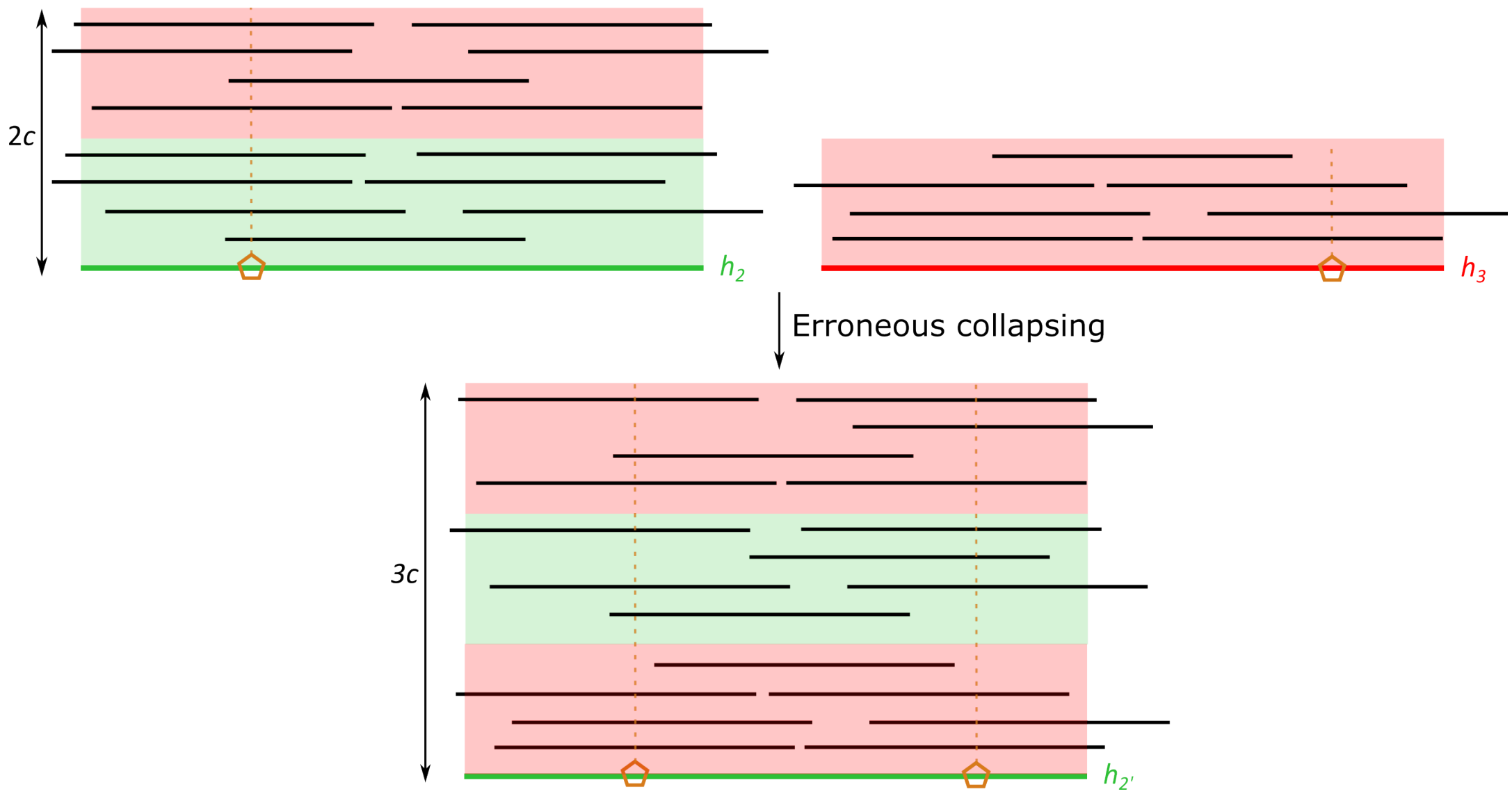

(b) Haplotig h_2 has LRs from both paternal and maternal haplotypes aligning to it because the alternate haplotig for that locus has not been assembled. Haplotig h_3 is similar to h_2. During assembly, both haplotypes are erroneously collapsed into one haplotig h_2' with $3c$ coverage.

Supplementary Figure S7: Erroneous haplotig duplication and collapsing.

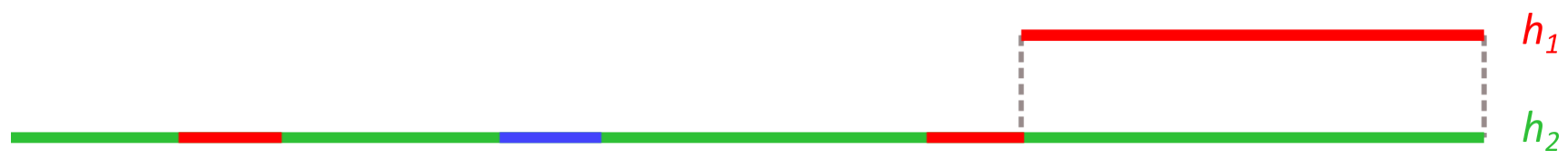

(a) Haplotig h_2 contains a few minor haplotype switches (shown in red) and an assembly artifact (shown in blue). Haplotig h_1 is the alternate haplotig with respect to the end of h_2.

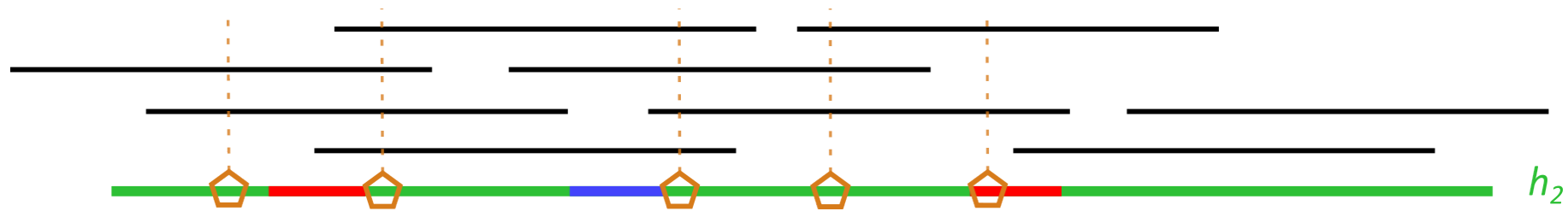

(b) LRs are aligned to haplotigs and variants are called. After quality filtering, 5 variants remain in h_2 (shown in orange).

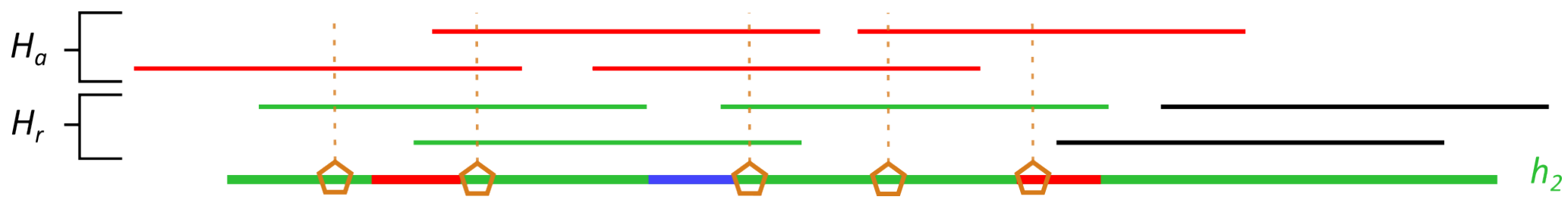

(c) The variants are phased and the LRs are haplotagged. Each LR is assigned to the reference haplotype or the alternate haplotype within the phase set. Two LRs at the end of h_2 cannot be haplotagged because they do not overlap any variant.

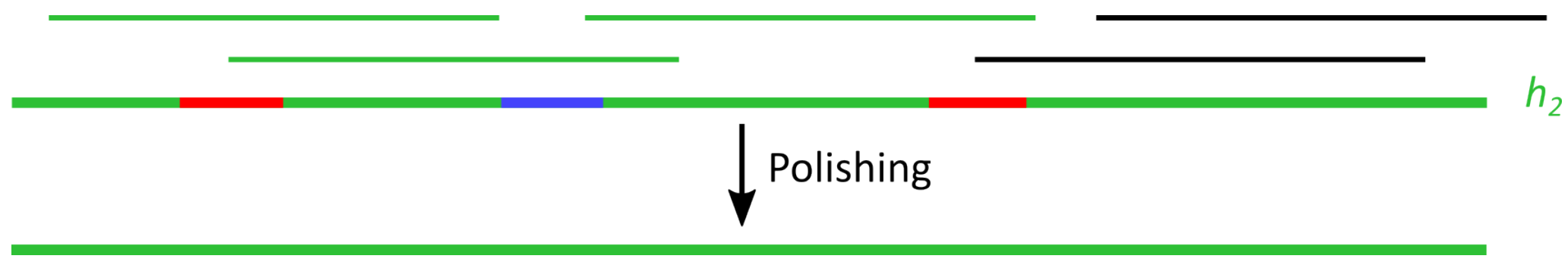

(d) Haplotig h_2 is polished using only the reference-tagged and untagged alignments.

Supplementary Figure S8: Haplotig polishing

adjacent phase sets to ensure that each resulting sub-haplotig does not contain alleles from different haplotypes. Second, the allele switch within each phase set of the CC candidates is investigated such that well-formed haplotype-resolved sequences should have no allele switch. A phase set is considered altered if at least 25% of its heterozygous SNVs incur an allele switch. The sequences of altered phase sets are separated from their haplotigs in order to avoid propagating errors to the clean sub-sequences during polishing.

Finally, the polishing step takes place on all remaining filtered and split haplotigs as shown in Figure S8. LRs are mapped to these haplotigs, then the alignments are filtered and haplotagged as previously described. For each phase set, the haplotype which as the most reference alleles among the phased variants is designated as the *reference haplotype* while the other haplotype is the *alternate haplotype*. Intuitively, the reference haplotype in each phase set has the most sequence similarity to the haplotig the LRs are aligned to. Subsequently, the haplotigs are polished with Flye using exclusively the unassigned and reference-tagged alignments in order to remove all alternate allele bases from the sequences. The polished haplotigs form a new haplotype-resolved assembly $\mathcal{A}_r{}'$.

3.6 Fine-grained haplotig cleaning

In previous steps, the haplotype coverage c was estimated from the coverage of $\mathcal{A}_c$ and used to perform a coarse-grained coverage filtering of the haplotigs in $\mathcal{A}_r$. However, c is inherently biased towards $\mathcal{A}_c$ and does not accurately represent the haplotype coverage. Furthermore, sequencing coverage is not necessarily uniform across the genome and can result in substantial variation of the coverage of individual haplotigs around the assembly coverage. As a result, we first refine c by computing the haplotype coverage c' directly from the coverage of high quality heterozygous SNVs in the CC haplotigs of $\mathcal{A}_r{}'$. From this refined coverage is derived a local haplotype coverage range for each haplotig in order to better phase CC candidates. Using variant coverage rather than read coverage enables a better representation of the haplotype coverage from an incomplete assembly.

Using the same methodology as for $\mathcal{A}_r$, LRs are aligned to $\mathcal{A}_r{}'$ and a list of CC candidates is created by selecting all haplotigs $h \in \mathcal{A}_r{}'$ with $\mathrm{cov}(\mathcal{A}_r{}', h) \geq 1.5 \cdot c$. Variants are called and phased on these candidates and a set $\mathcal{V}$ of phased heterozygous SNVs passing strict quality filters is retained. The global haplotype coverage c' is then defined as the mean coverage of the variants

in $\mathcal{V}$:

$$c' = \frac{1}{|\mathcal{V}|} \sum_{v \in \mathcal{V}} \frac{\text{cov}(\mathcal{A}_r', v)}{2} \quad (7)$$

A refined list of CC candidates is established using c' as the baseline haplotype coverage, i.e. haplotigs $h \in \mathcal{A}_r'$ with $\text{cov}(\mathcal{A}_r', h) \geq 1.5 \cdot c'$, as well as the set $\mathcal{V}'$ of high quality phased heterozygous SNVs they contain. A minority of variants in $\mathcal{V}'$ might be false positives because of the low coverage towards the extremities of haplotigs or the very high coverage in duplicated regions. To filter out these remaining false variants, a local haplotype coverage c'_h is defined as the mean coverage of variants $v \in \mathcal{V}'$ occurring in haplotig h and σ'_h as the average absolute deviation of the LR coverage in h with respect to c'_h. A variant $v \in \mathcal{V}'$ from a CC candidate h is only kept if it has a coverage in the range $c'_h - \sigma'_h \leq cov(\mathcal{A}_r', v) \leq c'_h + \sigma'_h$. The remaining variants of $\mathcal{V}'$ are of main importance to assign each haplotig to the paternal or maternal haplotype as described in the following.

3.7 Haplotig assignment to haplotypes

Haplotigs of $\mathcal{A}_r'$ are assigned to the paternal or maternal haplotype by counting the number of sub-sequences they share uniquely with either parental genome. To assist the assignment process, the set $\mathcal{V}'$ of high quality phased heterozygous SNVs overlapping CC candidates is used to generate the alternate haplotigs missing in $\mathcal{A}_r'$. Hence, LRs are mapped to $\mathcal{A}_r'$ and phased using the variants in $\mathcal{V}'$. Then, CC candidates of $\mathcal{A}_r'$ with at least one phase set are polished with Flye using the alternate-tagged and untagged alignments. The set $\mathcal{B}_r'$ of produced haplotigs approximates closely the missing alternate haplotigs of $\mathcal{A}_r'$.

A compacted de Bruijn graph is built with Bifrost [19] from all the 31-mers occurring in the sequences of $\mathcal{A}_r'$ and $\mathcal{B}'_r$. A k-mer in the graph is then assigned the paternal color, respectively maternal color, if it occurs twice or more in the paternal SRs, respectively maternal SRs. In the following, we use interchangeably color and haplotype to refer to the haplotype identity, paternal or maternal, assigned to k-mers. Subsequently, the graph is queried for all the k-mers composing the haplotigs of $\mathcal{A}_r'$ and $\mathcal{B}_r'$. The set of found mono-colored k-mers for a haplotig h is denoted K^1_h. Each haplotig h is initially assigned to the haplotype for which the color occurred the most in the k-mers of K^1_h.

A pair of assignments for a reference haplotig in $\mathcal{A}_r'$ and its corresponding alternate haplotig in $\mathcal{B}_r'$ is said to be *discordant* if both sequences are assigned to the same haplotype in which case at least one of the assignment is incorrect. Given a pair of assignments $(h \in H_x, h' \in H_y)$, $h \in H_x$ is considered the correct assignment and $h' \in H_y$ is considered invalid if:

1. at least 80% of the k-mers in K^1_h are colored with H_x but less than 80% of the k-mers in $K^1_{h'}$ are colored with H_y, otherwise

2. h' is composed by less than 0.05% of mono-colored k-mers and the number of k-mers colored with H_x in K^1_h is at least 20% more than the number of k-mers colored with H_y in $K^1_{h'}$, otherwise

3. the number of k-mers colored with H_x in K^1_h is at least twice more than the number of k-mers colored with H_y in $K^1_{h'}$ but neither K^1_h nor $K^1_{h'}$ have 80% of their k-mers colored with the same haplotype.

The first condition examines whether one of the assignment is based on a strong color consistency among the mono-colored k-mers while the other assignment is not. If none or both assignments show a strong color consistency, the second condition looks at whether one of the assignment is unreliable because based on a low number of mono-colored k-mers. Finally, if the second condition is not met for only one of the assignments, the last condition selects the assignment showing twice more confidence in its color consistency than the other. Once an assignment is selected as correct, the other assignment of the pair is "flipped" to the other haplotype it was initially assigned to. Finally, in the simple case where only one of the haplotigs in the pair is initially unassigned, its assignment is also changed to the haplotype which is not assigned to the other haplotig in the pair, e.g. $(h \in H_x, h' \in \varnothing)$ is changed to $(h \in H_x, h' \in H_y)$ with $x \neq y$.

Some haplotigs remain unassigned after flipping discordant assignments because they contain exclusively dual-color k-mers that are not unique to either parental genomes. Using a subset of assignments that are considered *safe* and the overlap of LRs between safe and unassigned haplotigs, the latter can be assigned to a haplotype. A haplotype assignment $h \in H_x$ is considered safe when at least 60% of the k-mers in K^1_h are colored with H_x and at least 0.025% of the k-mers in h

are mono-colored. All LRs are mapped to the haplotigs of $\mathcal{A}_r{}'$ and $\mathcal{B}_r'$, variants are called and phased with PMDV then the alignments are haplotagged using the previously phased heteroyzous SNVs. The alignments are transformed into a directed graph where the haplotigs are the vertices. An edge in the graph represents a non-ambiguous alignment spanning the start or the end of a safe haplotig and an unassigned haplotig. If at least 80% of the edges starting from, respectively ending on, an unassigned haplotig vertex connect to a single safe haplotig vertex, the unassigned haplotig is reassigned to the same haplotype as the safe haplotig is assigned to. However, if two reassignments are possible but conflicting because of incoming and outgoing edges connecting to two different safe haplotigs, the unassigned haplotig is left as is.

Finally, all haplotigs of $\mathcal{A}_r{}'$ and $\mathcal{B}_r{}'$ are aligned to the collapsed assembly $\mathcal{A}_c$. To ensure that no haplotig overlaps multiple contigs or the same contig multiple times, alignments are filtered to keep only primary alignments for which at least 99% of the haplotig length aligns to a contig. Subsequently, alignments are analyzed: if there exist a haplotig from $\mathcal{A}_r{}'$ and another haplotig from $\mathcal{B}_r{}'$ mapping to nearly the same positions on a contig of $\mathcal{A}_c$ and sharing the same haplotype assignment, these assignments collide: the assignment with the most k-mers colored with its assigned haplotype is kept while the other assignment is flipped.

3.8 Long read assignment to haplotypes

While the haplotigs of $\mathcal{A}_r{}'$ are assigned to the paternal or maternal haplotype, the assembly is still incomplete and fragmented. To resolve both issues, LRs must be assigned to haplotypes using their overlap to the haplotigs in order to later assemble each haplotype separately.

For this purpose, variants are phased and LR alignments are haplotagged with respect to $\mathcal{A}_r{}'$. A haplotig is said to be phased if it contains at least one phase set. For each phase set in a phased haplotig $h \in H_x$, all the LRs from reference-tagged primary alignments, respectively alternate-tagged primary alignments, are assigned to H_x, respectively H_y. However, LRs with untagged alignments are more challenging to assign. An alignment overlapping a phase set can be left untagged if it fails to meet the minimum requirements for haplotagging, e.g. overlapping a minimum number of heterozygous SNVs within the phase set. Alignments can also be untagged because they overlap unphased regions which are either homozygous stretches or multiple copies of a duplication collapsed into one.

Given a phased haplotig h with a set $\mathcal{S}_h$ of phased SNVs passing strict quality filters, we define respectively the reference coverage c_h^r, the alternate coverage c_h^a and the unphased coverage c_h^u as the coverage of variants in $\mathcal{S}_h$ using exclusively the reference-tagged, alternate-tagged and untagged alignments they overlap in their respective phase sets. Finally, we compute the coverage c_h of the phased variants in $\mathcal{S}_h$ using all the alignments they overlap. Intuitively, $c_h \approx c_h^r + c_h^a + c_h^u$. In previous sections, the haplotype coverage was first approximated from CC candidates to half their LR coverage for c and then half their phased variants coverage for c'. The assumption was that the coverage is always evenly distributed between the paternal and maternal haplotypes. However, coverage variation across the genome is not necessarily uniform and can result in unbalanced haplotype coverage distributions, e.g. for a phased haplotig h with no untagged alignments, the reference coverage c_h^r represents 60% of c_h. Hence, before assigning LRs from untagged alignments, it is necessary to determine whether they overlap heterozygous loci, homozygous loci or collapsed regions.

First, regions with an abnormally high coverage are thought to be copy-collapsed. Thus, all untagged alignments in phased haplotig h overlapping regions with coverage larger than $c_h + \frac{1}{2}\max(c_h^r, c_h^a) + \frac{1}{4}c_h^u$ are flagged as copy-collapsed. This coverage threshold is essentially the total coverage c_h plus half the coverage of one haplotype, i.e. $1.25 \cdot c_h$, but refined to account for the largest phased coverage in h and the unphased coverage. Hence, LRs from untagged primary alignments in these phased regions are simply assigned to the haplotype to which h is assigned to. Among the remaining regions are homozygous loci that could not be phased. These regions are assumed to have a coverage which is at least $c_h - \frac{1}{2}\max(c_h^r, c_h^a) - \frac{1}{4}c_h^u$. LRs from remaining primary untagged alignments in these regions are split as evenly as possible between paternal and maternal haplotypes. Finally, the remaining LRs from untagged alignments are in regions for which the coverage indicates only one haplotype. They are therefore attributed to the haplotype to which h is assigned to.

3.9 Dual assembly

Each group of LRs assigned to a parental haplotype is assembled independently with Flye, resulting in two preliminary haplotype assemblies $\mathcal{A}_d = (\mathcal{A}_1, \mathcal{A}_2)$ which undergo further filtering. First, the

two assemblies are inspected for erroneous duplications. In particular, two haplotigs ($h_1 \in \mathcal{A}_1, h_2 \in \mathcal{A}_2$) are filtered out if their respective coverage is less than 60% of c' and if they have at least 99.95% sequence similarity with each other. Second, the globally-corrected LRs are mapped to $\mathcal{A}_d$ and variants are called. The dual assembly is thereafter polished using all the variants passing the following quality filters:

- Genotype Quality (GQ) ≥ 20
- Variant Allele Frequency (VAF) ≥ 0.75
- Depth (DP) $\geq 0.75 \cdot c'$
- Depth (DP) $\leq 1.25 \cdot c$

Finally, the SRs are aligned to the polished haplotigs of $\mathcal{A}_d$, variants are called with bcftools and all haplotigs are polished using exclusively the homozygous calls.

4 Supplementary Methods 4: GraphTyper variant filtering

We recommend using the following GraphTyper variant filters when genotyping multiple samples (≥ 100):

- AAScore ≥ 0.5
- SeqYield $> 90\% \cdot |$ samples $|$
- Excess heterozygous Hardy-Weinberg equilibrium p-value $< 1^{-20}$

A variant must pass all three criteria to be included in the filtered set. The AAScore is computed by GraphTyper for each alternative allele. This score ranges from 0 to 1 and is calculated using a logistic regression model based on several quality metrics. SeqYield is the sequencing yield, i.e. how many sequenced samples got a genotype call at that site. The excess heterozygous Hardy-Weinberg equilibrium p-value is calculated using a Fisher's exact test. For multiallelic variant records, all the filters were calculated and applied separately for each alternative allele.

The only filter applied to the previous Icelandic callsets mapped with BWA-MEM is AAScore ≥ 0.5. To maintain consistency and fairness when comparing callsets, only that filter was applied to the Weaver callset.

5 Supplementary Methods 5: ICE sample collection

The HPRC-ICE pangenome reference was generated from 361 Icelandic individuals, 161 males and 192 females, whose genomes were sequenced with long read technologies: 32 individuals with PacBio HiFi, 312 individuals with ONT R9.4 and 17 individuals with both platforms. All individuals except one of the PacBio-sequenced individual were also sequenced with 151 bps paired-end Illumina reads at a mean depth of 41.37x (std: 11.15). Furthermore, the paternal and maternal genomes of all individuals sequenced with ONT and 22 of the individuals sequenced with PacBio HiFi were sequenced with 151bp paired-end Illumina reads at a minimum of 25x coverage. The 49 PacBio HiFi samples were sequenced on the PacBio Sequel IIe sequencing platform at a mean depth of 28.09x (std: 5.63). PacBio HiFi reads have a mean N50 of 17.40 kbps (std: 1.48 kbps) and a mean error rate of 1.12% (std: 0.07%). The 329 ONT samples were sequenced using R9.4 flowcells on the Promethion sequencing platform at a mean depth of 32.09x (std: 15.57). The ONT reads were basecalled with Guppy 5.0.11 using the SUP model with a mean read N50 of 21.39 kbps (std: 3.7 kbps) and a mean error rate of 6.98% (std: 0.71%). Long read error rate and coverage depth per sample are shown in Figure S9.

6 Supplementary Methods 6: ICE assemblies quality control

The ICE assemblies were evaluated for contiguity, completeness, quality, false duplications and phase switch. In particular, the k-mer completeness, sequence quality and false duplication rate

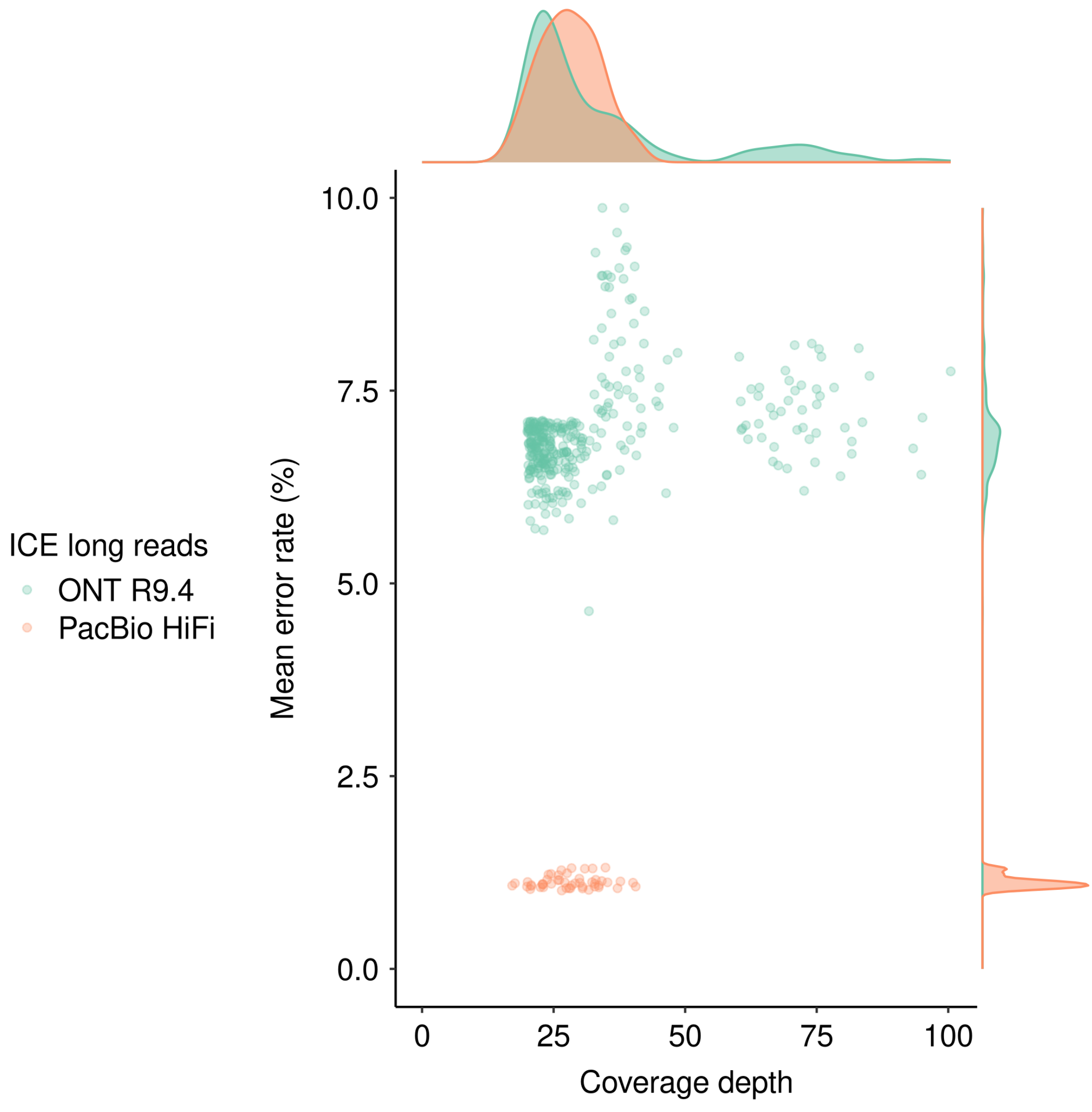

Supplementary Figure S9: Long read mean error rate and coverage depth of all samples ($n = 378$, including duplicated samples) used to assemble ICE haplotype assemblies, prior to any filtering performed in Section 8.1.

were quantified with respect to the Illumina short reads available for 48 of the ICE-PB assemblies and all of the ICE-ONT/ILMN assemblies. The phase switch error rate was evaluated for 22 of the ICE-PB assemblies and all of the ICE-ONT/ILMN assemblies as parental short reads are required for this evaluation.

6.1 Contiguity

Contiguity was assessed by measuring the N50 of each haplotype assembly using SeqKit [42]. The N50 of an assembly is the length of the shortest contig such that contigs longer than the N50 account for 50% of the assembly length. The mean haplotype N50 is 39.99 Mbps (std: 22.98 Mbps) for the ICE-PB assemblies and 4.47 Mbps (std: 3.66 Mbps) for the ICE-ONT/ILMN assemblies. Figure S10 shows that for about the same coverage depth, ICE-PB assemblies are about an order of magnitude more contiguous than the ICE-ONT/ILMN assemblies. Overall, increasing the input long read coverage depth also increases the output assembly contiguity for both the ICE-PB and the ICE-ONT/ILMN assemblies. Yet, the read downsampling performed by Emblask for samples with more than 50x coverage prevents the ICE-ONT/ILMN assemblies to reach more than 20 Mbps N50.

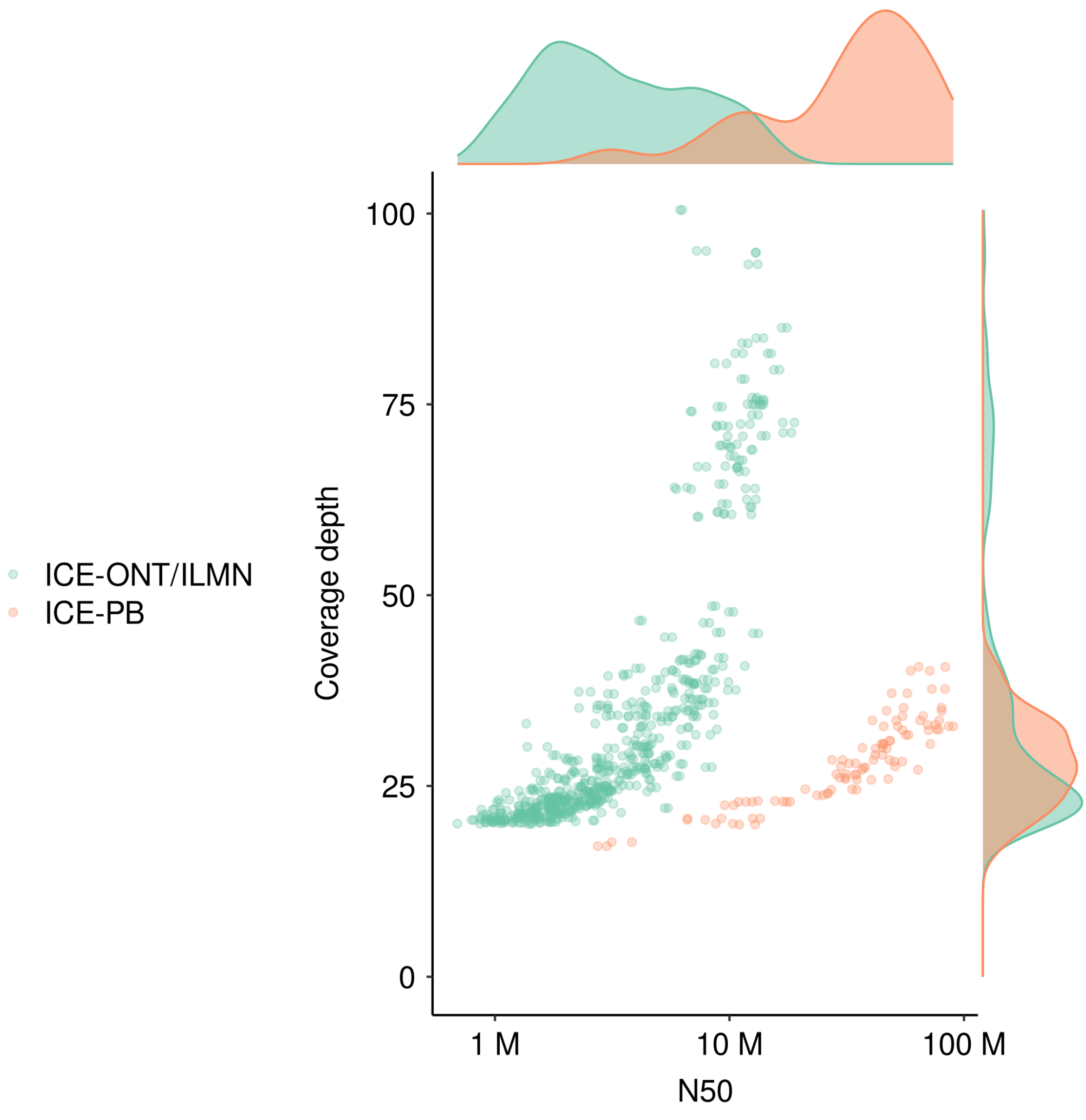

Supplementary Figure S10: N50 of the ICE-ONT/ILMN ($n = 658$) and ICE-PB ($n = 98$) haplotype assemblies, prior to any filtering performed in Section 8.1, with respect to the long read coverage depth.

6.2 Completeness and Quality

Completeness and quality were evaluated by Merqury [39] using the Illumina short reads available for the ICE samples. To compute the completeness of a dual assembly, k-mers were first extracted from the Illumina short reads, then counted and low-frequency k-mers were filtered out. The remaining k-mers, called *solid* k-mers, are deemed free of sequencing errors. The completeness is subsequently calculated as the proportion of all the solid k-mers which also occur in the assembly. The mean k-mer completeness is 99.17% (std: 0.33%) for the ICE-PB assemblies and 98.91% (std: 0.45%) for the ICE-ONT/ILMN assemblies. For comparison, the HPRC assemblies [33] were assembled using a similar methodology as the ICE-PB assemblies but with about 40% more PacBio HiFi coverage, resulting in a mean k-mer completeness of 99.57% (std: 0.10%).

The Quality Value (QV) is a log-scale metric which quantifies the proportion of k-mers composing an assembly which also are solid k-mers. A high QV indicates a high consensus accuracy, e.g. Q30 indicates a 99.9% sequence accuracy while Q50 indicates a 99.999% sequence accuracy. The mean QV is 54.96 (std: 3.16) for the ICE-PB assemblies and 50.31 (std: 2.01) for the ICE-ONT/ILMN assemblies. The HPRC assemblies have a mean QV of 55.36 (std: 1.30).

6.3 BUSCO

BUSCO [43] measures the completeness of an assembly from a gene content perspective. It reports on the presence, copy number and completeness status of universal single-copy orthologs in an assembly for a given lineage. The evolutionary expectation is that universal single-copy orthologs should be retained as a single copy in the assembly. Duplications and loss of these genes are possible but expected to be rare events. To compute BUSCO scores, compleasm [21] was used with the *primate* odb database containing 13,780 genes. As shown in Figure S11, the ICE-PB haplotype assemblies are very complete with a mean 97.59% (std: 1.13%) of the database found as complete and single-copy, similar to the mean 98.05% (std: 1.27%) of the single-copy completeness for the HPRC haplotype assemblies. The ICE-ONT/ILMN haplotype assemblies also showed a high level of completeness with a mean 96.07% (std: 1.79%) of the database found as complete and single-copy. While the proportion of complete but duplicated genes is similar between ICE and HPRC assemblies, the ICE-ONT/ILMN haplotype assemblies show a more elevated level of missing and fragmented genes compared to the ICE-PB haplotype assemblies. These results are in line with the contiguity and k-mer completeness results detailed in Sections 6.1 and 6.2.

6.4 False duplication

False duplications in assemblies are an important source of genome annotation errors and can lead to incorrect conclusions such as the functionalization of erroneously duplicated genes [23, 37]. Merqury detects erroneous homotype duplications by finding k-mers with abnormal copy numbers in a haplotype assembly with respect to the coverage of k-mers in the short reads of the same genome. To this end, solid k-mers are extracted from the short reads and the distribution of their multiplicities is computed. The two peaks in the resulting k-mer spectra points out to the k-mer coverage of heterozygous and homozygous loci. From the homozygous coverage peak is derived the set of k-mers that should only have two copies in the dual assembly, one per haplotype assembly. The false duplication rate is then calculated as the proportion of the two copies k-mers that occur more than once in a haplotype assembly. The mean false duplication rate is 0.38% (std: 0.15%) in the ICE-PB haplotype assemblies and 0.74% (std: 0.24%) in the ICE-ONT/ILMN haplotype assemblies. These results support that the ICE-ONT/ILMN assemblies are more prone to homotype duplications [38] for which the allele of a single-copy locus is assembled into multiple erroneous copies because of the residual noise of the overlapping ONT reads.

6.5 Phase switch

A phase switch is the change of allele from one haplotype to another within an assembled sequence. For a haplotype-resolved assembly, it can be caused by the ambiguous or erroneous overlap of long noisy reads, particularly in repeated and duplicated regions which are difficult to assemble, as well as incorrect haplotype assignments for trio-binned assemblies. Merqury estimates the phase switch errors from phased regions of the assembly containing k-mers that are unique to either the paternal or the maternal short reads. Up to 100 haplotype-specific k-mers switches are allowed by Merqury in any 20 kbps phase block stretch to account for local phase flips caused by consensus errors. The ICE-PB assemblies have a very low phase switch error rate, mean 0.07% (std: 0.03%) while the

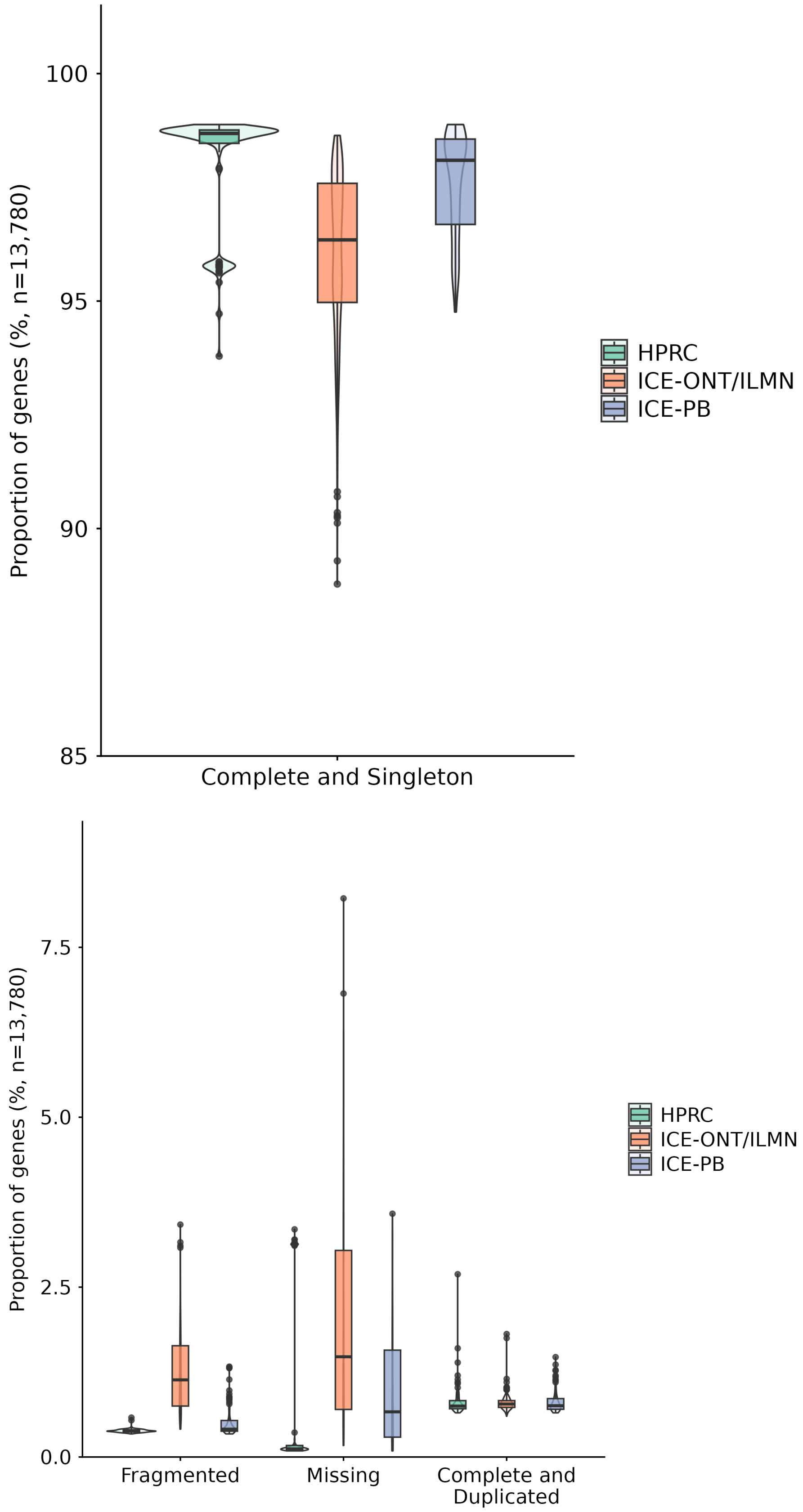

Supplementary Figure S11: BUSCO scores as the proportion of genes from the *primate* odb database which are either complete and single-copy, complete and duplicated, fragmented or missing in the HPRC ($n = 94$), ICE-PB ($n = 98$) and ICE-ONT/ILMN haplotype assemblies ($n = 658$), prior to any filtering performed in Section 8.1. The boxes represent the 25th-75th interquartile range (IQR) percentiles with the median shown as a line, whiskers extending to values within 1.5×IQR and outliers shown beyond the whiskers.

ICE-ONT/ILMN assemblies have an order of magnitude more phase switches, mean 0.64% (std: 0.5%).

7 Supplementary Methods 7: HG002 assembly

Assemblies of the HG002 sample were generated using long and short reads with similar characteristics as the reads used for the ICE-ONT/ILMN assemblies. The long reads dataset is composed of ONT R9.4 reads with an N50 of 33.12 kbps and an error rate of 6.77% from three flowcells basecalled with Guppy 5.0.6 using the SUP model. These reads were randomly subsampled to a 40x depth and the subsampled long reads were used in input of the assemblers. The short reads of the three trio members are 151 bps paired-end Illumina reads subsampled to a 30x depth. The generated haplotype assemblies of each assembler were then evaluated for the same QC metrics and the same tools as in Section 6, except for the BUSCO scores for which a more recent version of compleasm using the *primate* odb12 database was used. In addition, yak [5] was run on each dual assembly to evaluate their phasing with respect to k-mer databases computed from the parental short reads. For this purpose, the two haplotype assemblies produced by each pipeline were combined into one assembly, ensuring that each haplotig name was unique in the combined assembly. To establish a baseline, the T2T-Q100 haplotype-resolved dual assembly of HG002 was also evaluated for the sequence-based metrics while the GIAB small variant benchmark based on the same assembly was used to evaluate small variants in the benchmark regions.

Emblask was evaluated alongside the Shasta/HapDup pipeline [41, 24] which only uses LRs as input, and the HERRO/hifiasm-trio pipeline [46, 5] which also uses parental short reads for trio-binning in addition to the input LRs. Shasta was first run using the "Nanopore-Phased-May2022" configuration which is the recommended configuration for the ONT chemistry, read length and coverage of the input LRs. As a result, a haploid and a phased assembly were produced. The input LRs were then realigned to the haploid assembly using minimap2 with the parameters recommended by the HapDup pipeline. Using the haploid assembly and the realigned LRs, HapDup then produced a haplotype-resolved dual assembly. The HERRO correction was run through the Dorado tool [47] by swapping the default R10 model with the experimental R9.4 model. The correction was performed in two steps. The first step required a compute node with 1TB of memory to perform an all-vs-all alignment of the input LRs. The second step required a compute node with a GPU, an Nvidia L40 with 48GB of memory, to perform the inference from the alignment and output the corrected long reads. As recommended by the HERRO pipeline, the corrected reads were chopped to 30 kbps and filtered to retain only those longer than 10 kbps for the hifiasm assembly. The resulting corrected reads were then assembled with hifiasm-trio using the paternal and maternal yak databases computed from the same parental short reads as used in input of Emblask. Results are shown in Table S1.

	T2T-Q100	Emblask	Shasta HapDup	HERRO hifiasm-trio
Input reads		LR<br>Trio SR	LR	LR<br>Parental SR
QV - Merqury	64.613 [a]	**49.354**	31.345	38.908
Completeness (%) - Merqury	99.402 [a]	**99.013**	98.310	98.948
N50 (Mbps) - SeqKit	150.564	7.511	**22.850**	20.569
False duplication (%) - Merqury	0.386 [a]	1.042	**0.504**	1.160
Switch error (%) - Merqury [b]	0.016 [a]	0.759	**0.494**	0.713
Switch error (%) - Yak [b]	0.876 [a]	**5.080**	5.258	5.746
Hamming error (%) - Yak	0.692 [a]	**4.553**	19.173	4.647
SNPs F1 (%) - vcfdist		**96.741**	90.391	96.732
Indels F1 (%) - vcfdist		**79.107**	17.295	36.020
Switch errors - vcfdist		1,080	2,429	**530**
Flip errors - vcfdist		**11,428**	24,227	13,476
Switch NGC50 (Mbps) - vcfdist		8.384	4.812	**18.639**
Switch-flip NGC50 (Mbps) - vcfdist		**0.426**	0.212	0.415
BUSCO-S (%) - compleasm	98.025	97.145	99.080	96.610
BUSCO-D (%) - compleasm	0.645	0.940	0.560	1.645
BUSCO-F (%) - compleasm	0.005	0.310	0.125	0.070
BUSCO-M (%) - compleasm	1.325	1.6	0.235	1.675

[a] The QV of the T2T-Q100 HG002 assembly v1.1 is 77.8 as computed per Merqury using a hybrid database of PacBio HiFi long reads and high coverage Illumina short reads, as described in [34]. The Merqury database used in this experiment was built from 30x depth Illumina reads, resulting in a lower QV. Other sequence-based metrics comparing to k-mer databases computed from short reads may differ.
[b] The differences between the switch errors computed by the two tools is due to different employed methodologies. Merqury reports large-scale switches that tolerate a limited number of smaller local errors, as described in Section 6.5, while yak has a more fine-grained approach that is more sensitive to local switches.

Supplementary Table S1: Comparison of different sequence-based and variant calling metrics for the HG002 haplotype-resolved dual assemblies from three different assembly pipelines. All three pipelines use the same ONT R9 dataset in input. Additionally, Emblask uses trio Illumina data in input while HERRO/hifiasm-trio uses parental Illumina data. Best results are highlighted.

Tables S2 and S3 compare the small variant called from the Emblask assemblies with dipcall to the calls from the input long reads aligned to GRCh38 and called with PMDV. In the latter callset, variants were subsequently phased with Margin using the appropriate ONT R9 model and only PASS variants were analyzed. In each callset, all structural variants were removed, multi-allelic variants were split into bi-allelic representations followed by a left-alignment and normalization of all variants with respect to the reference genome. The two callsets were compared with vcfdist to the GIAB phased small variant benchmark truthset based on the T2T-Q100 haplotype-resolved dual assembly 1.1 in the benchmark regions and all regions. As the truthset variants do not contain missing genotypes in the benchmark regions, all variants with a fully missing genotype (./. or .|.) or half-missing genotype were removed from each callset for that comparison. For the all regions comparison, all half-missing genotype variants were retained. To ensure an unbiased comparison, all variants called from the Emblask assemblies that are in non-confident regions, as established by dipcall, were retained.

The Emblask-dipcall variant callset contains 1.34% to 9.76% more variants than the LR-PMDV-Margin callset. While the SNP calls from the LR-PMDV-Margin set were 0.72% more accurate

	Emblask assemblies Dipcall	LRs PMDV-Margin
Variants	4,404,944	4,346,376
SNPs F1 (%) - vcfdist	96.741	**97.463**
Indels F1 (%) - vcfdist	**79.107**	48.956
Phase blocks - vcfdist	**24**	2,375
Switch errors - vcfdist	1,080	**203**
Flip errors - vcfdist	11,428	**4,267**
Switch NGC50 (Mbps) - vcfdist	**8.384**	3.273
Switch-flip NGC50 (Mbps) - vcfdist	0.426	**0.990**

Supplementary Table S2: Comparison of the phased small variants from the Emblask assemblies and from the aligned ONT R9 long reads in the confident regions of the HG002 small variant truthset. Best results are highlighted.

	Emblask assemblies Dipcall	LRs PMDV-Margin
All variants	5,425,202	4,942,404
SNPs F1 (%) - vcfdist	**91.905**	89.908
Indels F1 (%) - vcfdist	**78.255**	48.166
Phase blocks - vcfdist	**24**	4,149
Switch errors - vcfdist	2,193	**718**
Flip errors - vcfdist	12,924	**4,904**
Switch NGC50 (Mbps) - vcfdist	**6.157**	3.198
Switch-flip NGC50 (Mbps) - vcfdist	0.410	**0.905**

Supplementary Table S3: Comparison of the phased small variants from the Emblask assemblies and from the aligned ONT R9 long reads in all the regions of the HG002 small variant truthset. Best results are highlighted.

in the confident regions benchmark, they were 1.99% less accurate in the all region benchmark compared to the Emblask-dipcall variants. Indel calls from the Emblask-dipcall set were consistently more accurate than the LR-PMDV-Margin calls in both benchmarks by more than 30%. The Emblask-dipcall set has one phase set per chromosome, for a total of 24 phase sets in these benchmarks, while the LR-PMDV-Margin callset has 2,375 to 4,149 phase sets, mainly due to phase sets being interrupted across the genome by long regions of homozygosity or inadequate coverage. As a result, the LR-PMDV-Margin callset had less switch errors and flip errors than the Emblask-dipcall set. Yet, in both comparisons, the Emblask-dipcall set had a larger NGC50 than the LR-PMDV-Margin set when interrupting phased blocks by switch errors but a lower NGC50 when interrupting phased blocks by switch and flip errors.

8 Supplementary Methods 8: HPRC-ICE

As described in Section 1, the pangenome graph model used in this study relies on a graph incrementally built from a reference sequence with minigraph [31]. Non-reference assemblies are progressively merged into the graph by splitting existing vertices and adding new vertices representing long haplotype subsequences which diverge from the assemblies already present in the graph. As a result, the output rGFA graph does not contain small variants from the input assemblies. The small variants are overlaid from a phased VCF file to the vertices of the pangenome graph. For this purpose, we used the HPRC Minigraph-Cactus v1.1 VCF file and merged it with the small variants found in our Icelandic assemblies.

8.1 Icelandic assemblies selection

Based on the Icelandic assembly evaluations, a set of strict criteria was defined to only select the best assemblies for the pangenome graph:

- 95% k-mer completeness or more
- 90% BUSCO single-copy completeness or more
- Q45 or higher
- 0.8 Mbps haplotig N50 or higher
- 2% phase switch error rate or less
- 2.5% false duplications rate or less

Some of the ICE-PB assemblies could not be evaluated for some of these criteria because of the absence of offspring or parental Illumina short reads. From the 658 haplotype assemblies in the ICE-ONT/ILMN set, 636 haplotype assemblies (96.65%) passed all the QC criteria and 22 assemblies (3.35%) failed at least one of the QC requirements. All the 98 haplotype assemblies in the ICE-PB set passed all the QC requirements that could be computed. For the 17 genomes with ICE-PB and ICE-ONT/ILMN assemblies available, 16 of the ICE-PB assemblies were used and one of the ICE-PB assemblies was set aside for validation.

8.2 Genome Assembly

All PacBio HiFi samples were assembled with hifiasm or hifiasm-trio [5] if the parental short reads were available. All ONT R9.4 samples were assembled alongside the trio short reads with the Emblask pipeline described in Section 3. ONT datasets with more than 50x coverage depth were automatically downsampled to 50x coverage by Emblask prior to each assembly step of the pipeline in order to keep running time and memory usage tractable. In the following, we refer to the dual assemblies of Icelandic genomes as ICE-PB if assembled from PacBio HiFi reads and as ICE-ONT/ILMN if assembled from ONT-Illumina reads. The Merqury files for the quality and completeness of the HPRC assemblies were only available for 42 of the assemblies in the HPRC S3 repository.

8.3 Joint genotyping

The VCF file for the HPRC samples (Section 15) contains variants converted to VCF format from the sequence graph produced by the Minigraph-Cactus pipeline [17] for the haploid T2T-CHM13 assembly and 44 HPRC dual assemblies with many variants nested in a snarl [35]. To obtain non-overlapping sites, bubbles were popped with vcfbub [15] and only sites with alleles shorter than 100 kbps were initially kept. Then, similarly to the preprocessing of the ICE variants, all structural variants and variants with a star allele were removed, followed by a left-alignment and normalization of all remaining variants with respect to the base assembly. Finally, each diploid genotype was split into two haploid genotypes.

The HPRC haploid variant calls were merged with their ICE haploid counterparts using bcftools. The resulting multi-sample VCF contains 787 haplotype samples: 602 samples from the ICE-ONT/ILMN haplotype assemblies, 96 samples from the ICE-PB haplotype assemblies, 88 samples from the HPRC haplotype assemblies (HG002, HG005 and NA19240 were set aside for internal validation) and the haploid T2T-CHM13 assembly. Complex variant decomposition and merging took place thereafter. This step is necessary as it frequently occurs in multi-sample VCFs that a variant shared by multiple samples is called with different representations among the assemblies of the carriers, especially for complex variants, because multiple alignments with the optimal scoring are possible for an haplotig [26]. Consequently, the merged HPRC-ICE variant set contains an inflated number of variants with inaccurate frequencies. To solve this issue, vcfwave [16] was used to realign the variant alleles and split complex alleles into simpler representations which can be merged. A side effect of the vcfwave simplification is that duplicated variants with same alleles and same positions but different genotypes are sporadically created. This is the result of breaking down adjacent complex variants into simpler primitives with at least one of these primitives shared by two or more adjacent complex variants. For a set of duplicated variants, one of the record is kept but the genotypes of all the duplicates are merged in it to maximize the number of alternate allele carriers.

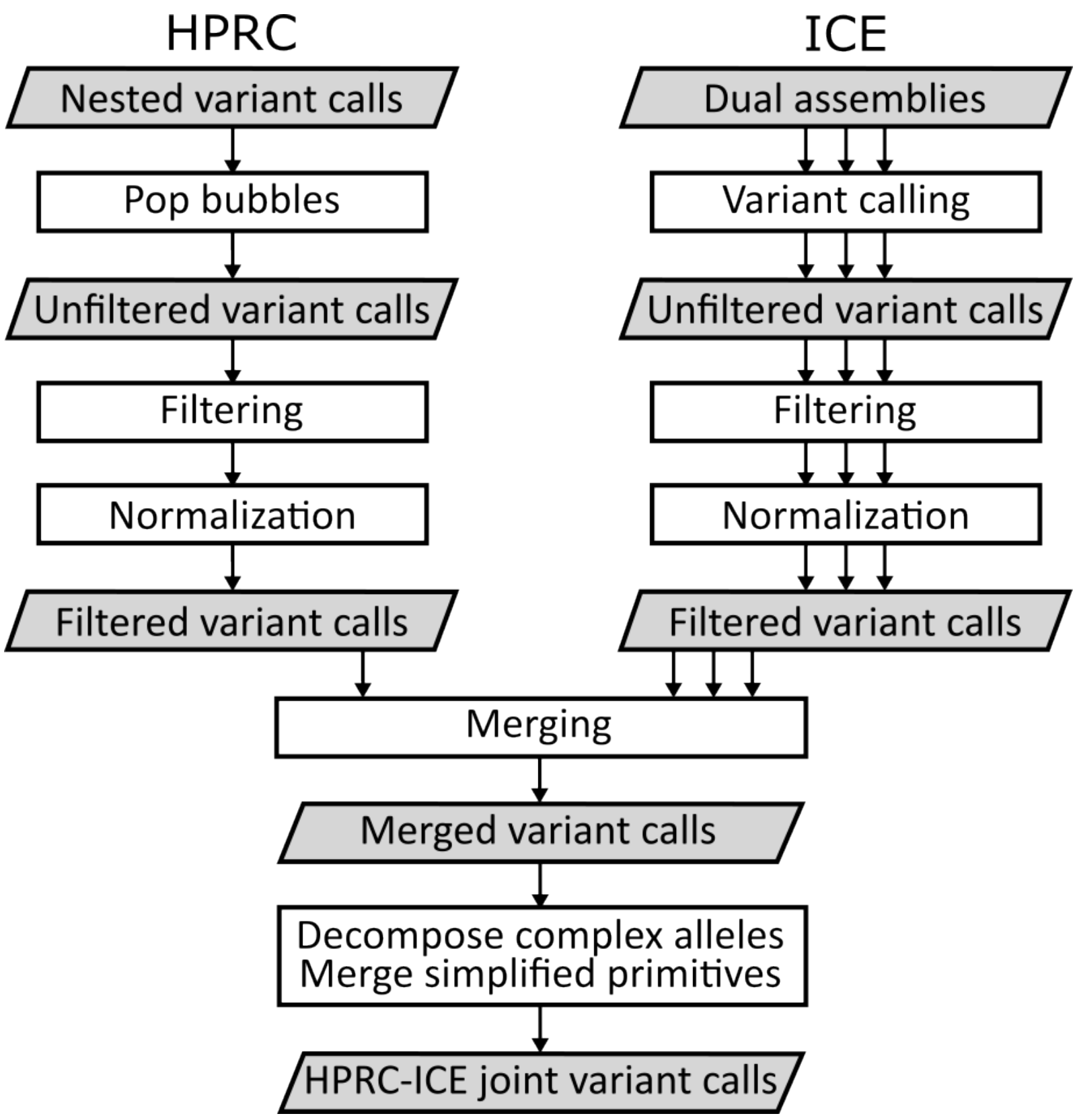

Supplementary Figure S12: Flowchart of the joint variant calling of the ICE assemblies and merging with the HPRC variants. Data are represented in grey background parallelograms while operations are shown in white background rectangles.

After simplification, the HPRC-ICE variant set totals 51,418,313 bi-allelic variants of which

75.87% (39,011,184) are SNVs, 22.82% (11,735,493) are indels, 0.75% (388,035) are MNPs and 0.55% (283,601) are some other types of variants such as complex substitutions. Among these variants, 36.88% (14,388,640) of the SNVs and 38.87% (4,562,237) of the indels are singletons (allele count of 1). This is a slight increase compared to the normalized HPRC variant set which is composed of 33.94% (7,014,770) singleton SNVs and 30.80% (1,999,783) singleton indels. The transitions to transversions ratio (Ti/Tv) is 1.80 compared to 2.03 for the HPRC set.

8.4 Variant calling

Small variants (substitutions and indels shorter than 50 bps) were called from all the ICE dual assemblies using a modified version of dipcall [29]. The first dipcall modification is an upgrade of the default minimap2 version from 2.18 to 2.24 which substantially improves improves long reads and assembly alignments over previous versions. The second modification is the use of wider Z-drop score parameters in minimap2 to improve the contiguity of the assembly alignments [22]. The output phased variant calls were left-aligned, normalized and multiallelic variants were split into bi-allelic variants with bcftools [8]. Furthermore, variant calls from the ICE-ONT/ILMN assemblies were filtered and polished using the trio short reads as detailed in Sections 8.4.1 and 8.4.2. Finally, all structural variants, variants with a star allele and variants with a missing genotype or a half-missing half-reference genotype (".|., ".|0" or "0|.") were filtered out. Besides the variants, dipcall also outputs the variant call confident regions where at least one haplotig longer than 50 kbps from each parent aligns with a minimum mapping quality of 5 but conditioned to no other haplotig at least 10 kbps long aligning to the same region.

The quality of the variant calls from the ICE-ONT/ILMN assemblies was assessed using a selection of individuals for whom Illumina, ONT R9.4 and PacBio HiFi reads were available. Specifically, 15 individuals with ICE-PB and ICE-ONT/ILMN dual assemblies were selected because ICE-PB assemblies were also computed for their parents. A set of safe variant calls was created from the selected 15 ICE-PB assemblies using the ICE-PB assemblies of their parents for mendelian consistency filtering. Then, we evaluated the quality of the variant calls from the ICE-ONT/ILMN assemblies using the safe variants and all the variants from these ICE-PB assemblies as ground truth.

8.4.1 False positives filtering

False positives variant calls were filtered out by eliminating variants for which the sequences do not occur in the Illumina short reads of the same individual. First, a compacted de Bruijn graph is built from the Illumina short reads with Bifrost. All 63-mers occurring only once in the short reads are deemed to contain a sequencing error and are not used for the construction of the graph. Subsequently, for each variant is extracted the sequence containing all 63-mers overlapping its alternate allele which will be used to query the de Bruijn graph. However, such a sequence can overlap the positions of nearby false positive variants which would invalidate the query. Hence, sequences for all possible combinations of alternate and reference alleles for these adjacent variants are generated as queries. As the number of possible sequences grows quadratically with the number of neighboring variants, a variant is only considered for false positive filtering if it has less than 12 neighboring variants in a 62 bps window around the variant, otherwise it is automatically retained. Finally, the previously built de Bruijn graph is queried for all the generated variant sequences. If no sequence associated to a variant has at least 70% of its k-mers found in the graph, the variant is considered to be a false positive and is filtered out as illustrated in Figure S13. Once all detected false positive calls for the alternate alleles have been filtered out, the filtering process is applied to the reference alleles.

8.4.2 False homozygous calls detection

Incorrect homozygous calls can also be detected and changed to heterozygous using the Illumina short reads. As previously described in Section 8.4.1, the sequences overlapping a homozygous variant and its neighbouring counterparts are extracted for the two haplotypes with the exception that the alternate allele of the variant of interest if replaced with the reference allele. If at least one of the generated sequences is found in the de Bruijn graph computed from the Illumina short reads but is not found in the assembly, the variant is annotated as a heterozygous call. One must now determine whether the reference allele of this heterozygous calls is from the paternal or the maternal haplotype. For this purpose, a de Bruijn graph is built for each parental Illumina short reads set. These graphs are queried for the sequences which were a positive hit in the offspring

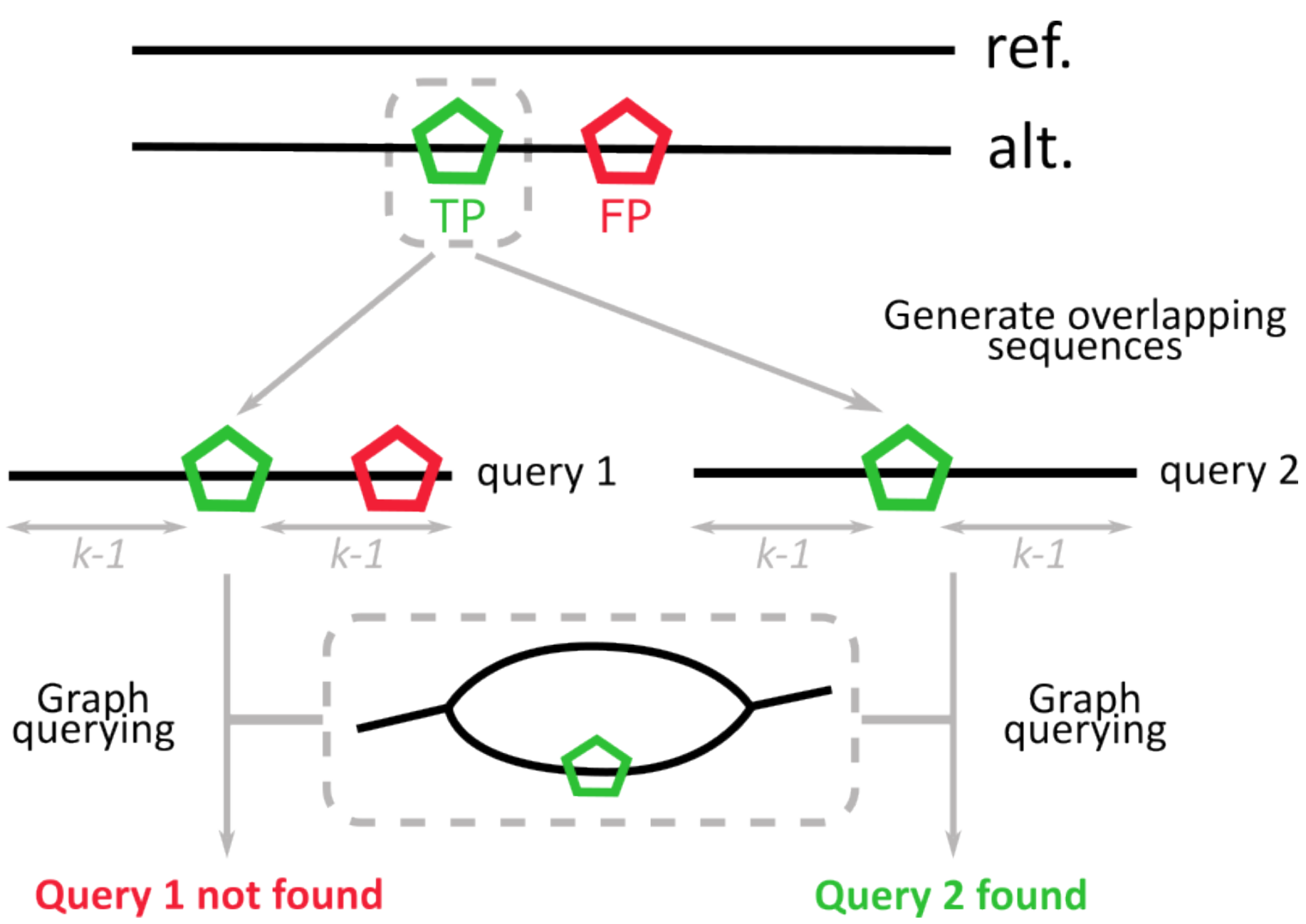

Supplementary Figure S13: Graph querying to detect whether a variant is a false positive. An existing heterozygous variant (in green) is tested for false positive removal. The sequence containing all its overlapping k-mers is extracted (query 1). As this sequence overlaps a nearby variant (in red) which could be a false positive, another sequence with the reference allele of the neighboring variant is also generated (query 2). The de Bruijn graph is queried for both sequences and all the k-mers composing the second sequence are found, indicating that the analyzed variant is a true positive and the adjacent variant is a false positive.

de Bruijn graph but not in the assembly. If such a sequence has at least 60% of its k-mers found in the paternal graph, respectively maternal graph and the number of found k-mers is 30% more than for the other parental graph, then the reference allele is attributed to the paternal haplotype, respectively maternal haplotype. If the reference allele cannot be attributed to either haplotype, the homozygous genotype remains for that variant call.

8.4.3 Safe variants

As the ICE-PB assemblies outperformed the ICE-ONT/ILMN assemblies on most QC metrics, we created a set of safe variants passing strict quality filters for 15 of the ICE-PB assemblies for which parent-offspring trio Illumina and PacBio HiFi reads were available. At first, all small variants from these assemblies were considered as safe candidates. Then, potential mis-assemblies were detected in each of the 15 ICE-PB assemblies with the Flagger pipeline [33] which identifies anomalies in the coverage distribution of long reads aligned to their assemblies. All variants overlapping assembly regions marked by Flagger as erroneous, collapsed or falsely duplicated were removed from the set of safe candidates. Finally, a mendelian consistency check was performed with rtg tools [7] on the remaining variants: variants with inconsistent genotypes with respect to the parental calls were also removed from the safe variants set. The positions overlapping the filtered out variants were removed from the confident regions. After filtering, each sample had an average 3,448,587 safe SNV calls and 614,762 safe indel calls.

8.4.4 ICE-ONT/ILMN evaluation

Variant calls from the confident regions of the ICE-ONT/ILMN assemblies were first evaluated with respect to the safe variants from the 15 ICE-PB assemblies. For this purpose, vcfdist [11] was employed as it takes into account the phasing of the variants in its assessment and allows partial credit to almost correct variant calls. Compared to the safe variants, the ICE-ONT/ILMN variants have a mean F1 score of 98.91% (std: 0.32%) for the SNVs and 83.98% for the indels (std: 3.66%). While the SNV calls accuracy is consistent with what has been reported in the literature for variants called from aligned ONT R9.4 reads [40, 24], the indels are notably more accurate than when called from the reads themselves. The ICE-ONT/ILMN variants were also evaluated with respect to all the ICE-PB variants with a mean 97.98% of the ICE-ONT/ILMN confident regions

overlapping the ICE-PB confident regions. As a result, the ICE-ONT/ILMN variant calls had a similar accuracy as with the safe variants set: mean F1 score of 98.70% for the SNVs and 87.59% for the indels. For comparison, we also evaluated an HG002 assembly made by the Shasta-HapDup pipeline from ONT R9.4 long reads only. Similarly to the methodology previously described, small variants were called from the dual assembly with dipcall, normalized with bcftools and compared with vcfdist to the GIAB benchmark variants v1.0 from the HG002 T2T-Q100 assembly. The evaluation was limited to the intersection of the HG002 T2T-Q100 benchmark regions and the dipcall confident regions for the Shasta-HapDup assembly. The resulting F1 scores are 96.99% for the SNVs and 35.14% for the indels, a more than two-fold reduction in accuracy compared to the indels from the ICE-ONT/ILMN assemblies.

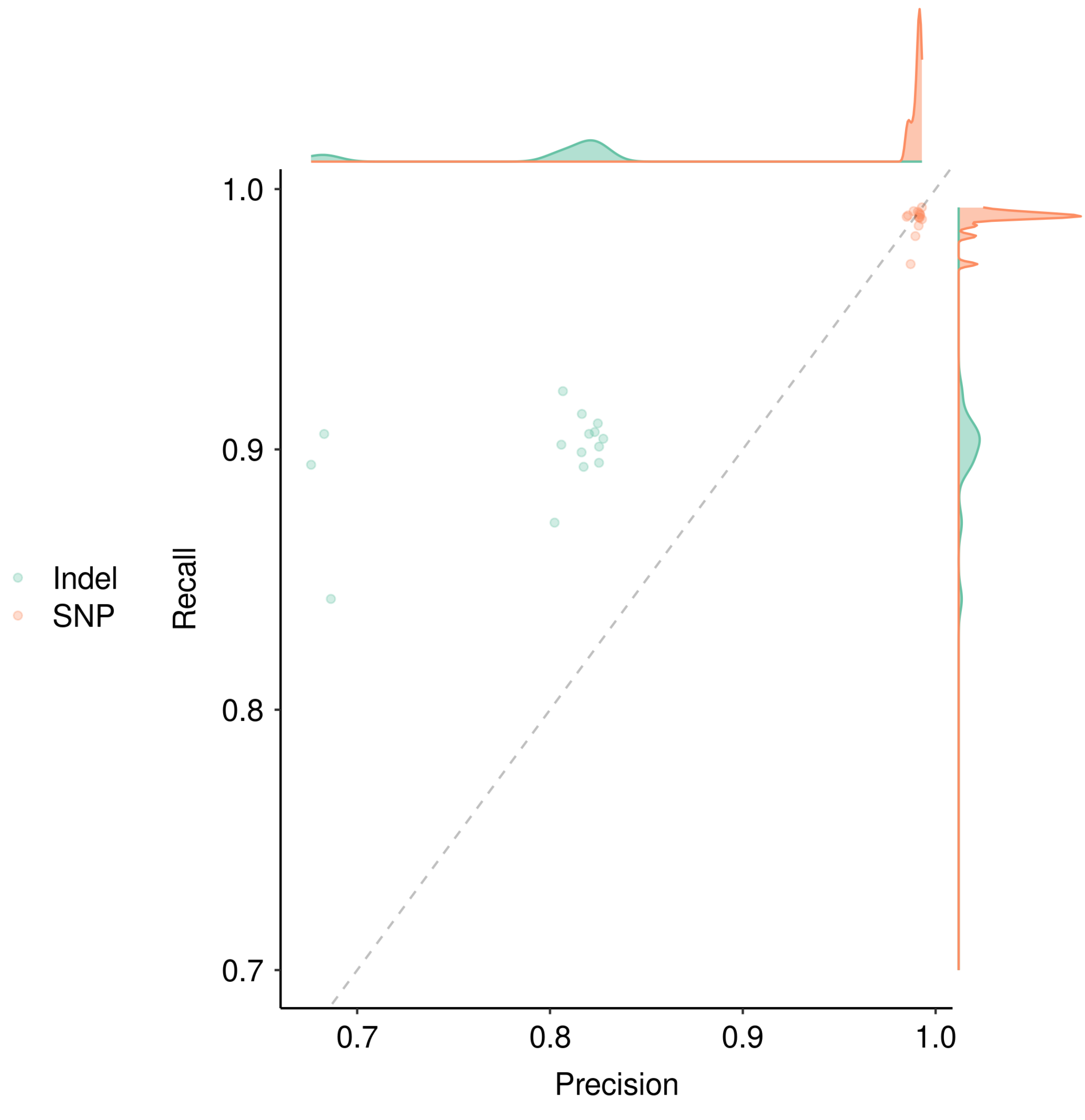

Supplementary Figure S14: Precision and recall of the phased ICE-ONT/ILMN variant calls with respect to the safe variants from the ICE-PB assemblies ($n = 15$).

From the comparison of ICE-ONT/ILMN variants to all the ICE-PB variants, false positives and false negatives were stratified by type of regions using the GA4GH/T2T/GIAB stratification regions [27] for GRCh38 as shown in Figure S15. As expected, the majority of indel errors are located within homopolymers and tandem repeats given that homopolymers are the primary source of errors for ONT R9.4 reads [6]. Additionally, all ICE-ONT/ILMN variant calls were evaluated within the regions of GRCh38 which are dark to Illumina short reads [13]. A region is said to be *dark* to a type of read if the mapping quality is poor or the coverage depth is low in that region. Indeed, the GC sequence composition is a known bias factor for the coverage of Illumina reads [4] while low-mapping quality is inherently caused by long duplicated regions resulting in ambiguous mapping locations. Additionally, regions with low base qualities in the overlapping reads were also annotated as dark. Formally, the position of a base in a reference primary scaffold is annotated as

dark if any of the following conditions applies:

- at least 90% of the aligning reads have a mapping quality below 10,
- it has less than 5x coverage,
- the mean quality of the aligning read bases is less than 20

The dark regions were computed from the Illumina short reads of 123 Icelanders aligned to GRCh38 and regions shorter than 30 bps were filtered out. Excluding gaps in the reference sequence, 73.3 Mbps of regions were annotated as dark, 58.09 Mbps of which are also annotated as low-mappability regions in the GA4GH/T2T/GIAB stratification regions. As the safe variants set contains only a few variants overlapping the dark regions, the evaluation was done with all the variants from the ICE-PB assemblies as ground truth. The resulting mean F1 score of the ICE-ONT/ILMN variants in the dark regions is 87.31% for SNVs and 76.88% for the indels. This decrease of accuracy with respect to non-dark regions is due to the difficulty to Illumina-correct ONT reads in complex regions of the genomes involving repeats and duplications. The variant calls from the Shasta-HapDup HG002 dual assembly were also evaluated in the dark regions, resulting in a mean F1 score of 89.24% for SNVs and 41.37% for the indels.

Finally, the phasing of variants was assessed with vcfdist by calculating the supercluster switch error rate. Similarly to the phase switch error from Merqury in Section 6.5, a supercluster switch error occurs when the phasing of two adjacent superclusters of variants is inverted. The mean supercluster switch error rate of the ICE-ONT/ILMN variant calls is 0.03% with respect to the safe variants and 0.032% with respect to all the variants from the ICE-PB assemblies. Variants from the Shasta-HapDup HG002 dual assembly have a supercluster switch error rate of 0.057%.

9 Supplementary Methods 9: Read simulation experiment

We evaluated mapping performance by simulating 10 million paired-end short reads from the T2T-Q100 HG002 assembly using Mason [20]. The length of the reads was 151 bps, resulting in approximately 1x genome-wide sequence coverage. The reads were subsequently mapped to the HPRC-ICE pangenome reference with Weaver, to the HPRC Minigraph-Cactus AF-filtered (d9) graph with Giraffe [45], to the DRAGEN [2] multi-genome graph reference with DRAGEN and to GRCh38 with BWA-MEM [30]. We additionally extracted the subset of reads that were simulated fully within GRCh38 low-mappable regions according to the GIAB stratifications [12]. These regions span 248.9 Mbps (8%) of the reference genome and are challenging for short read mapping.

10 Supplementary Methods 10: Comparison of running times

We compared the running time of mapping the short reads of 19 Icelandic samples that had been sequenced with various sequence coverage. Weaver was run with the HPRC-ICE graph, Giraffe with the allele frequency filtered d9 graph and BWA-MEM with GRCh38. The input read data were in gzip compressed FASTQ files and all mappers were given 12 CPU threads on an Intel Xeon Platinum 8358 CPU. We could not include a comparison to DRAGEN as it requires specialized FPGA-accelerated hardware to run. The reported running times refer to the CPU time reported by slurm for the entire process generating an analysis ready BAM/CRAM file. Besides mapping, this includes sorting records by position, duplicates marking, BAM/CRAM compression and BAM/CRAM indexing performed by samtools [32].

11 Supplementary Methods 11: Commands and scripts

11.1 rGFA graph construction

At the time of the HPRC-ICE pangenome reference construction, the only available minigraph rGFA file was based on the HPRC assemblies v1.0. In order to use the latest assemblies available, we ran minigraph using the available HPRC assemblies v1.1 with GRCh38 set as the reference assembly.

The command used for construction was

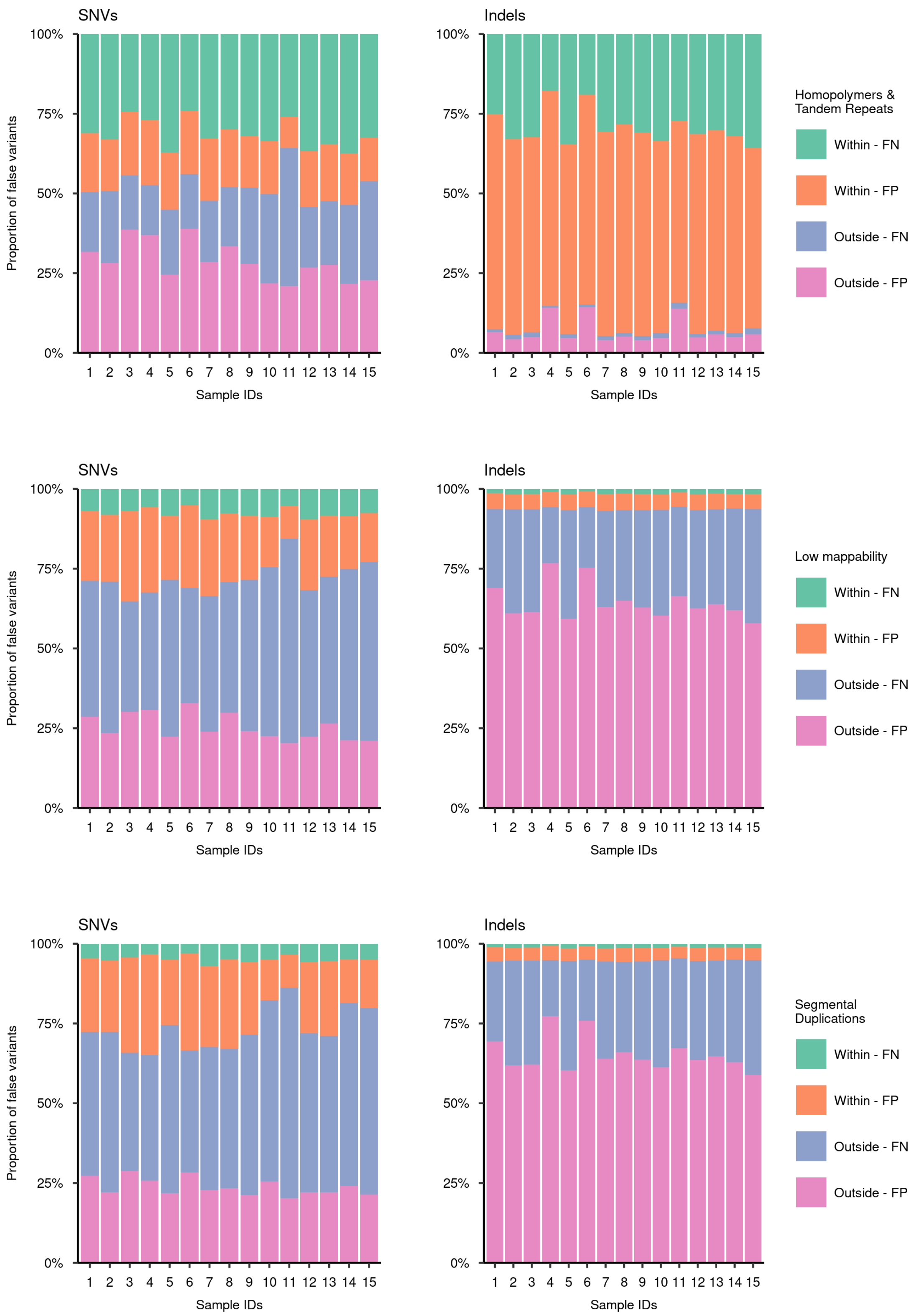

Supplementary Figure S15: Proportion of false variants in the ICE-ONT/ILMN assemblies stratified by type of regions: homopolymers and tandem repeats extended of 5 bps, low-mappability regions and segmental duplications. Blue segments, respectively green segments, are the false variants occurring within, respectively outside, the specified regions.

```
minigraph -cxggs -q 10 -L 1000 -l 200000 ${assemblies} \
> minigraph.grch38.hprc-v1.1.gfa
```

Variants with low allele frequencies introduce additional alternative paths in the graph which could result in ambiguous short read mapping as well as increased mapping time. Consequently, only variants with common paths (AF$>$ 10%) were included, as is used in AF-filtered Giraffe graphs [17]. To achieve this, we mapped each haplotype assembly sequence back to the graph and called its path with the following command:

```
minigraph -xasm -t24 --call minigraph.grch38.hprc-v1.1.gfa ${assembly}
```

We then found the non-reference nodes which are included in the path fewer than 9 assemblies. We excluded those nodes from all Weaver mapping experiments by skipping those nodes when finding the minimizers of the graph.

11.2 HG002 T2T Q100 simulation study

We extracted small variants of each HG002 haplotype into separate VCF files. The haplotypes include the autosomes and chromosome X. We used the following script to generate the VCFs:

```
#!/bin/bash
for h in 1 2; do
  bcftools view --no-version GRCh38_HG002-T2TQ100-V1.0_smvar.vcf.gz |
    awk -v h="${h}" -v OFS=$’\t’ ’$1 ~ /^#/{print; next}
  $7=="."{
  n = split($10, a, ":")
  if (n != 2) { next } # Expect two fields (GT and AD). Only GT will be used

  m = split(a[1], b, "|")
  if (m != 2) { next } # Not diploid call, skip

  split($5, alt, ",")
  if (alt[b[h]] != "*") { # Ignore calls of the deleted allele
    printf $1"\t"$2"\t"$3"\t"$4"\t"$5"\t"$6"\t"$7"\t"$8"\tGT\t"b[h]"\n"
  }
}’ |
    bcftools norm -m- - | # Split into biallelics
    awk ’$1 ~ /^#/ || $10 == 1’ |
    awk ’$1 != "chrY"’ |
    grep -v "^##contig=<ID=chrY," |
    bgzip -@12 -c > "hg002.hap${h}.vcf.gz"

  tabix -p vcf "hg002.hap${h}.vcf.gz"
done
```

We then prepared a FASTA file containing only those chromosomes (`ref.fa`) and simulated 10 million reads on each haplotype VCF using

```
#!/bin/bash
seed=${1:-1}$
for h in 1 2; do
  mason_simulator --version-check 0 \
    --seed $((seed + (h - 1) * 1000)) \
    --num-threads 6 \
    --input-reference ref.fa \
    --read-name-prefix "s." \
    --illumina-read-length 151 \
    --num-fragments 5000000 \
    --input-vcf "hg002.hap${h}.vcf.gz" \
    -o  "hap${h}.${seed}.r1.fq.gz" \
    -or "hap${h}.${seed}.r2.fq.gz" \
    -oa "hap${h}.${seed}.bam"
done
```

11.3 Running DRAGEN

Running Illumina DRAGEN requires specialized hardware with an FPGA. We installed DRAGEN 4.2.4 and ran the following command to map and genotype:

```
dragen \
  -r dragen_4.2_altmasked \
  -1 <fq1> \
  -2 <fq2> \
  --output-file-prefix <sampleID> \
  --output-directory <sampleID> \
  --intermediate-results-dir /staging/tmp \
  --enable-map-align true \
  --enable-map-align-output true \
  --enable-sort true \
  --output-format CRAM \
  --enable-duplicate-marking true \
  --enable-variant-caller true \
  --vc-emit-ref-confidence GVCF \
  --vc-enable-vcf-output true
```

12 Supplementary Methods 12: Map data

Birthplaces for UK Biobank participants to plot the frequency map were obtained from Data-Fields 129 and 130. Birthplaces were then assigned to the following administrative divisions: the ceremonial counties of England, the historic counties of Wales and the 1975 local government areas of Scotland. Adjacent divisions were then grouped such that each group was represented by at least 2,400 participants. Geospatial analyses and plotting were performed in R using the packages sf [36] and ggplot2 [53].

1. England ceremonial counties, Wales historic counties: Ordnance Survey OpenData Boundary-Line downloaded from: `https://ukdataservice.ac.uk/help/data-types/census-data/`. Licensed under the Open Government License v3, `https://www.nationalarchives.gov.uk/doc/open-government-licence/version/3/`

2. Scotland 1975 local government areas: UK Data Service `http://discover.ukdataservice.ac.uk/catalogue/?sn=5819&tyep=Data%20catalogue`, retrieved from `http://census.ukdataservice.ac.uk/get-data/boundary-data.aspx`. Contains NRS data © Crown copyright and database right 2021m contains OS data © Crown copyright and database right 2021. Licensed under the Open Government License v3.

3. Northern Ireland, Republic of Ireland, Isle of Man, Channel Islands: GADM v36, accessed at `https://gadm.org/`. Licensed under the GADM license.

The map of Iceland was obtained from `https://mapsvg.com/maps/iceland` and the map of the world was obtained from `https://mapsvg.com/maps/world`. Licensed under CC0 1.0 Universal.

13 Supplementary Methods 13: Weaver-HPRC

We highlight in the main manuscript that Weaver improves variant calling in low confidence regions of the HG002 truth set. Those comparisons are based on Weaver and the HPRC-ICE pangenome reference. One tool included in the comparison is Giraffe with the HPRC pangenome reference, either by using the pangenome reference as is or subsetting it into a personalized pangenome reference (PPR). Using Weaver mapping to the HPRC pangenome instead of the HPRC-ICE instead mapped to the HPRC pangenome resulted in a marginally lower precision and sensitivity in low-confidence regions (Figure S16) and genome-wide (Figure S17). These results suggest the ICE portion of HPRC-ICE has a minor contribution to the accuracy of Weaver.

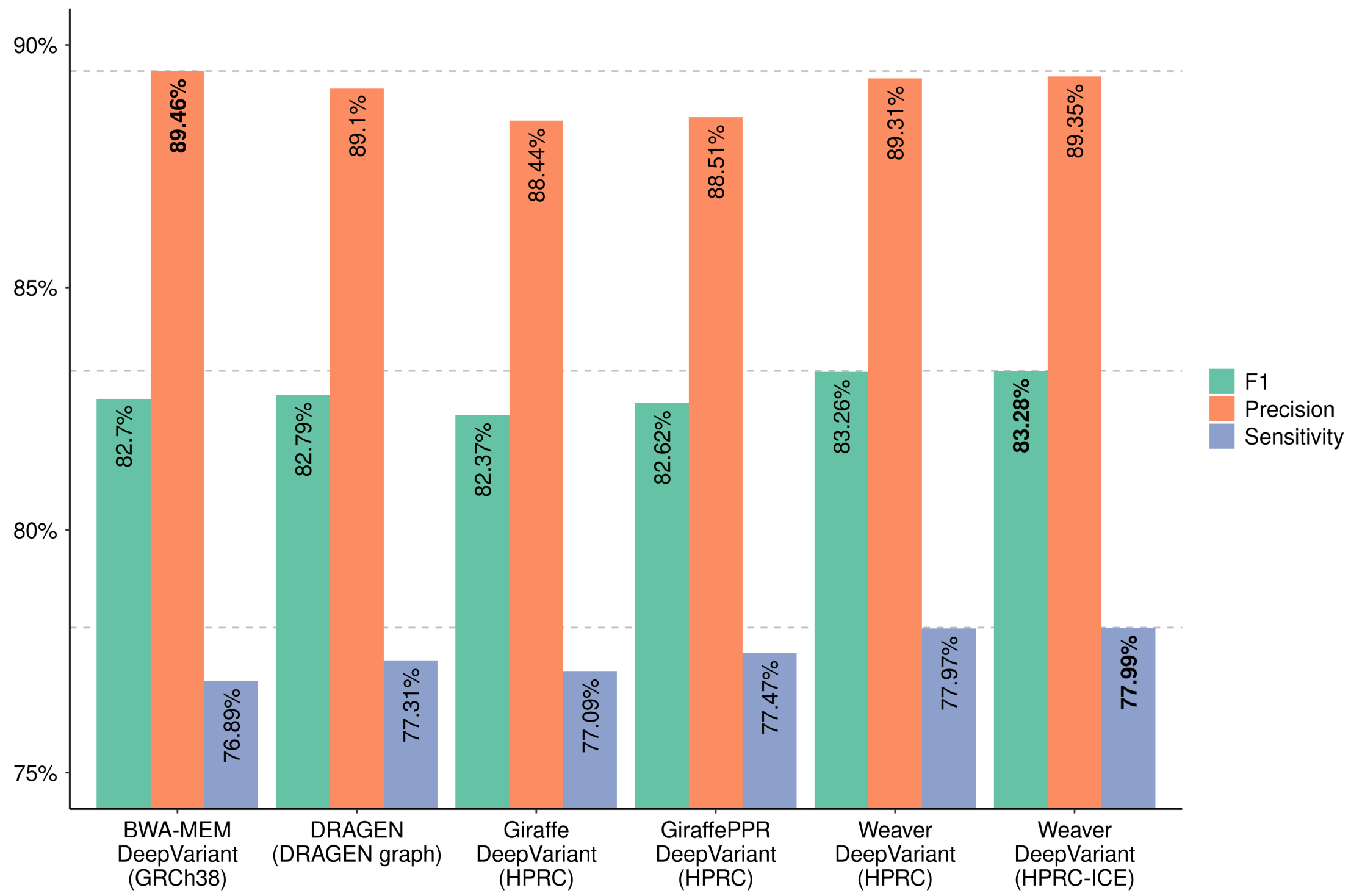

Supplementary Figure S16: F1 metric, precision and sensitivity of benchmarked methods compared to the HG002 T2T-Q100 v1.1 low confidence regions.

The same observation is made when comparing to the safe variants of the assembled Icelanders (Figure S18).

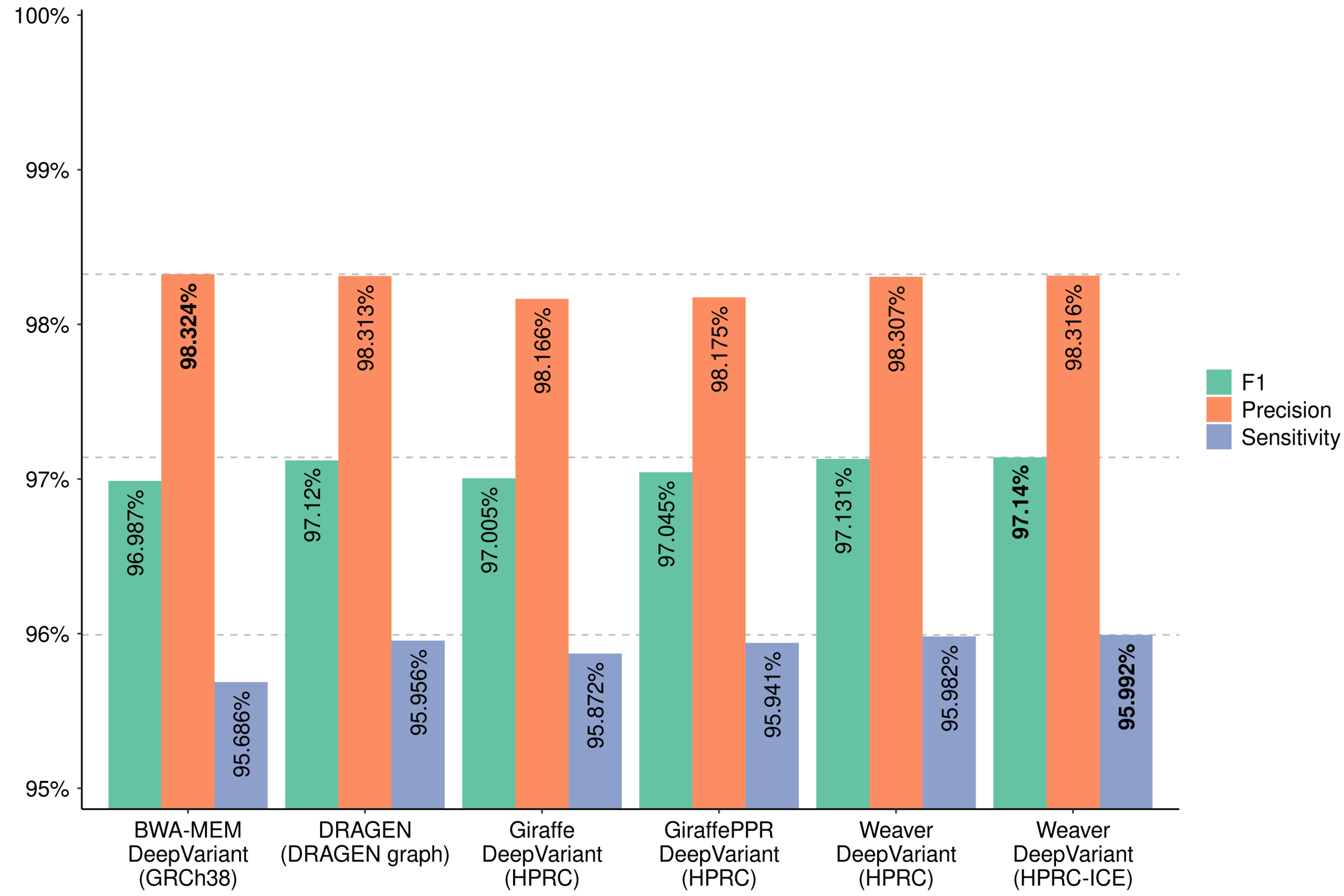

Supplementary Figure S17: F1 metric, precision and sensitivity of benchmarked methods compared to the HG002 T2T-Q100 v1.1 genome-wide. The figure extends Figure 4f in the main manuscript by also showing results for Weaver-DeepVariant (HPRC) and Giraffe-DeepVariant without the personalized pangenome reference (PPR).

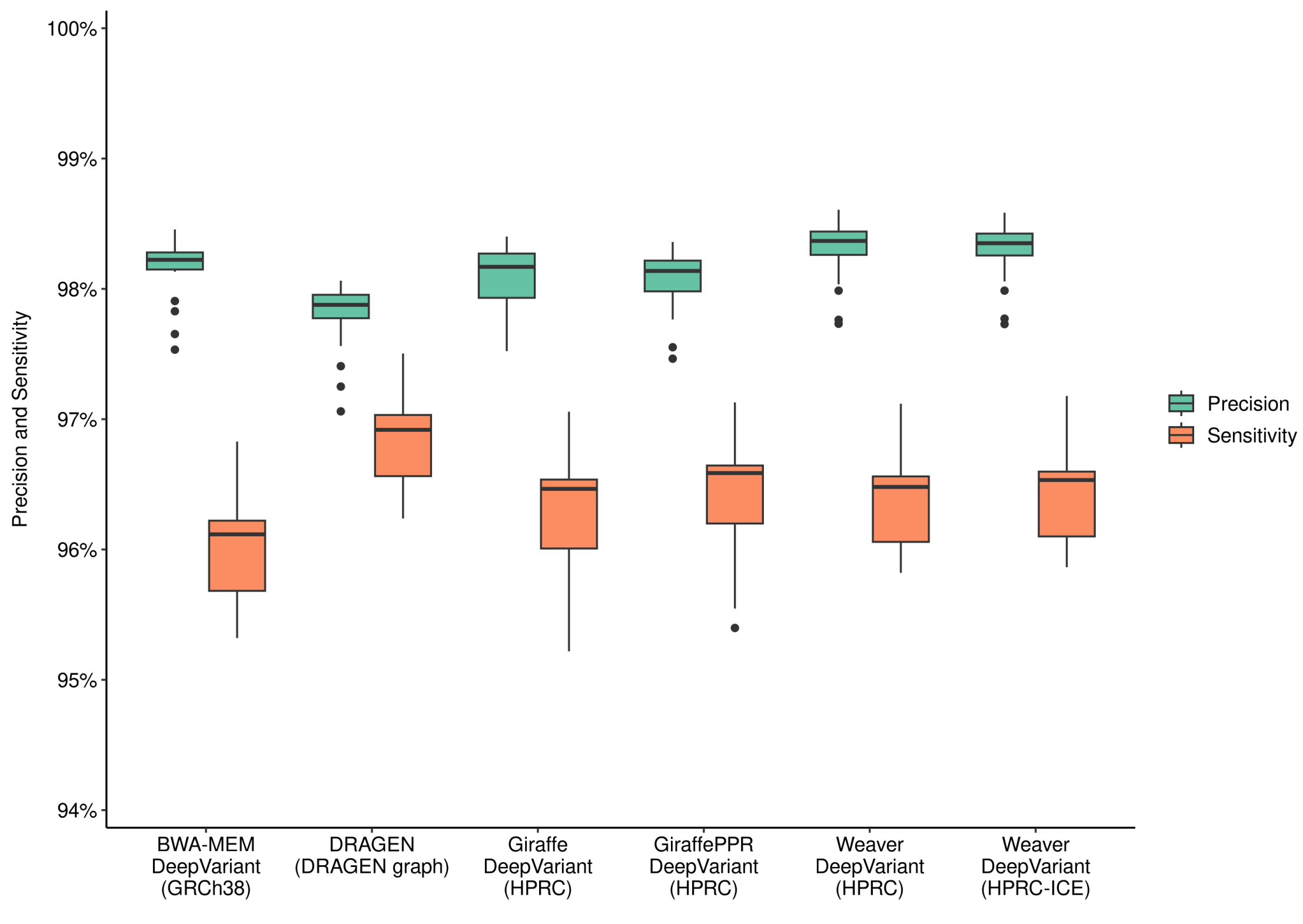

Supplementary Figure S18: Precision and sensitivity across the full genome of the benchmarked methods compared to the safe variants of the Icelandic assemblies ($n = 19$). The boxes represent the 25th-75th interquartile range (IQR) percentiles with the median shown as a line, whiskers extending to values within 1.5×IQR and outliers shown beyond the whiskers.

14 Supplementary Methods 14: Tools

Tool	Version	Link
BWA	0.7.16a	`https://github.com/lh3/bwa`
hifiasm	0.16.0	`https://github.com/chhylp123/hifiasm`
yak	0.1-r69	`https://github.com/lh3/yak`
compleasm	0.2.1	`https://github.com/huangnengCSU/compleasm`
minimap2	2.24	`https://github.com/lh3/minimap2`
dipcall	0.3	`https://github.com/lh3/dipcall`
bcftools	1.18	`https://www.htslib.org/`
samtools	1.18	`https://www.htslib.org/`
Bifrost	1.2.1	`https://github.com/pmelsted/bifrost`
Flagger	0.1	`https://github.com/mobinasri/flagger`
rtg tools	3.10.1	`https://github.com/RealTimeGenomics/rtg-tools`
vcfdist	2.3.2	`https://github.com/TimD1/vcfdist`
Mason2	2.0.9	`https://github.com/seqan/seqan/blob/main/apps/mason2`
minigraph	0.20-r559	`https://github.com/lh3/minigraph`
Emblask	0.1.0	`https://github.com/DecodeGenetics/emblask`
Weaver	0.2.0	`https://github.com/DecodeGenetics/weaver`
vcfwave	1.0.9	`https://github.com/vcflib/vcflib`
vcfbub	0.1.0	`https://github.com/pangenome/vcfbub`
SeqKit	2.0	`https://bioinf.shenwei.me/seqkit/`
Merqury	1.3	`https://github.com/marbl/merqury`
GraphTyper	2.7.7	`https://github.com/DecodeGenetics/graphtyper`
DeepVariant	1.6.0 (pre-release)	`https://github.com/google/deepvariant`
Crumble	0.9.1	`https://github.com/jkbonfield/crumble`
vg	1.51.0	`https://github.com/vgteam/vg`
Shasta	0.14.0	`https://paoloshasta.github.io/shasta/`
HapDup	0.12.0	`https://github.com/KolmogorovLab/hapdup`
Dorado	1.1.1	`https://github.com/nanoporetech/dorado`

Supplementary Table S4: Tools used in this study with version numbers and accession links.

15 Supplementary Methods 15: Data

Resource	Version	Link
GA4GH/T2T/GIAB stratification regions	3.3	https://ftp-trace.ncbi.nlm.nih.gov/ReferenceSamples/giab/release/genome-stratifications/v3.3/GRCh38@all/
GIAB HG002 T2T-Q100 small variant benchmark	V0.018 20240716	https://ftp-trace.ncbi.nlm.nih.gov/ReferenceSamples/giab/data/AshkenazimTrio/analysis/NIST_HG002_DraftBenchmark_defrabbV0.018-20240716/
	V0.019 20241113	https://ftp-trace.ncbi.nlm.nih.gov/ReferenceSamples/giab/data/AshkenazimTrio/analysis/NIST_HG002_DraftBenchmark_defrabbV0.019-20241113/
GIAB HG002 T2T-Q100 assembly	1.1	https://s3-us-west-2.amazonaws.com/human-pangenomics/T2T/HG002/assemblies/hg002v1.1.fasta.gz
GIAB HG002 short reads	-	https://ftp-trace.ncbi.nlm.nih.gov/ReferenceSamples/giab/data/AshkenazimTrio/HG002_NA24385_son/NIST_HiSeq_HG002_Homogeneity-10953946/NHGRI_Illumina300X_AJtrio_novoalign_bams/HG002.GRCh38.60x.1.bam
GIAB HG003 short reads	-	https://ftp-trace.ncbi.nlm.nih.gov/ReferenceSamples/giab/data/AshkenazimTrio/HG003_NA24149_father/NIST_HiSeq_HG003_Homogeneity-12389378/NHGRI_Illumina300X_AJtrio_novoalign_bams//HG003.GRCh38.60x.1.bam
GIAB HG004 short reads	-	https://ftp-trace.ncbi.nlm.nih.gov/ReferenceSamples/giab/data/AshkenazimTrio/HG004_NA24143_mother/NIST_HiSeq_HG004_Homogeneity-14572558/NHGRI_Illumina300X_AJtrio_novoalign_bams/HG004.GRCh38.60x.1.bam
HG002 ONT R9.4	2020.11	s3://ont-open-data/gm24385_2020.11/
CARD HG002 ONT R9.4 assembly	-	https://s3-us-west-2.amazonaws.com/human-pangenomics/index.html?prefix=publications/Napu_paper_ONT_Coriell_SingleFC_2023/HG002_R9/assembly/
HPRC assemblies	Y1	https://github.com/human-pangenomics/HPP_Year1_Assemblies
HPRC GRCh38 Minigraph-Cactus small variants	Y1	https://s3-us-west-2.amazonaws.com/human-pangenomics/index.html?prefix=pangenomes/freeze/freeze1/minigraph-cactus/
HERRO R9.4.1 correction model	0.1	https://zenodo.org/records/12683277/files/model_R9_v0.1.pt?download=1
Ensembl genes	v112	https://www.ensembl.org/info/data/ftp/index.html

Supplementary Table S5: Data used in this study with version numbers and accession links.

References

[1] Dmitry Antipov, Anton Korobeynikov, Jeffrey S McLean, and Pavel A Pevzner. hybridSPAdes: an algorithm for hybrid assembly of short and long reads. *Bioinformatics*, 32(7):1009–1015, 2016.

[2] Sairam Behera, Severine Catreux, Massimiliano Rossi, Sean Truong, Zhuoyi Huang, Michael Ruehle, Arun Visvanath, Gavin Parnaby, Cooper Roddey, Vitor Onuchic, Daniel L Cameron, Adam English, Shyamal Mehtalia, James Han, Rami Mehio, and Fritz J Sedlazeck. Comprehensive and accurate genome analysis at scale using DRAGEN accelerated algorithms. *bioRxiv*, 2024.

[3] Denis Bertrand, Jim Shaw, Manesh Kalathiyappan, Amanda Hui Qi Ng, M Senthil Kumar, Chenhao Li, Mirta Dvornicic, Janja Paliska Soldo, Jia Yu Koh, Chengxuan Tong, et al. Hybrid metagenomic assembly enables high-resolution analysis of resistance determinants and mobile elements in human microbiomes. *Nat. Biotechnol.*, 37:937–944, 2019.

[4] Yen-Chun Chen, Tsunglin Liu, Chun-Hui Yu, Tzen-Yuh Chiang, and Chi-Chuan Hwang. Effects of GC bias in next-generation-sequencing data on de novo genome assembly. *PLoS ONE*, 8(4):e62856, 2013.

[5] Haoyu Cheng, Gregory T Concepcion, Xiaowen Feng, Haowen Zhang, and Heng Li. Haplotype-resolved de novo assembly using phased assembly graphs with hifiasm. *Nat. Methods*, 18:170–175, 2021.

[6] Chien-Shun Chiou, Bo-Han Chen, You-Wun Wang, Nang-Ting Kuo, Chih-Hsiang Chang, and Yao-Ting Huang. Correcting modification-mediated errors in nanopore sequencing by nucleotide demodification and reference-based correction. *Commun. Biol.*, 6(1215), 2023.

[7] John G Cleary, Ross Braithwaite, Kurt Gaastra, Brian S Hilbush, Stuart Inglis, Sean A Irvine, Alan Jackson, Richard Littin, Mehul Rathod, David Ware, Justin M Zook, Len Trigg, and Francisco M De La Vega. Comparing Variant Call Files for Performance Benchmarking of Next-Generation Sequencing Variant Calling Pipelines. *bioRxiv*, 2015.

[8] Petr Danecek, James K Bonfield, Jennifer Liddle, John Marshall, Valeriu Ohan, Martin O Pollard, Andrew Whitwham, Thomas Keane, Shane A McCarthy, Robert M Davies, et al. Twelve years of SAMtools and BCFtools. *Gigascience*, 10(2):giab008, 2021.

[9] Joshua Casey Darian, Ritu Kundu, Ramesh Rajaby, and Wing-Kin Sung. Constructing telomere-to-telomere diploid genome by polishing haploid nanopore-based assembly. *Nat. Methods*, 21:574–583, 2024.

[10] Alex Di Genova, Elena Buena-Atienza, Stephan Ossowski, and Marie-France Sagot. Efficient hybrid de novo assembly of human genomes with WENGAN. *Nat. Biotechnol.*, 39:422–430, 2021.

[11] Tim Dunn and Satish Narayanasamy. vcfdist: accurately benchmarking phased small variant calls in human genomes. *Nat. Commun.*, 14(8149), 2023.

[12] Nathan Dwarshuis, Divya Kalra, Jennifer McDaniel, Philippe Sanio, Pilar Alvarez Jerez, Bharati Jadhav, Wenyu Huang, Rajarshi Mondal, Ben Busby, Nathan D Olson, et al. The GIAB genomic stratifications resource for human reference genomes. *Nat. Commun.*, 15(9029), 2024.

[13] Mark TW Ebbert, Tanner D Jensen, Karen Jansen-West, Jonathon P Sens, Joseph S Reddy, Perry G Ridge, John SK Kauwe, Veronique Belzil, Luc Pregent, Minerva M Carrasquillo, et al. Systematic analysis of dark and camouflaged genes reveals disease-relevant genes hiding in plain sight. *Genome Biol.*, 20(97), 2019.

[14] Hannes P Eggertsson, Hakon Jonsson, Snaedis Kristmundsdottir, Eirikur Hjartarson, Birte Kehr, Gisli Masson, Florian Zink, Kristjan E Hjorleifsson, Aslaug Jonasdottir, Adalbjorg Jonasdottir, et al. Graphtyper enables population-scale genotyping using pangenome graphs. *Nat. Genet.*, 49:1654–1660, 2017.

[15] Erik Garisson. vcfbub: popping bubbles in vg deconstruct VCFs. *Zenodo*, 2022.

[16] Erik Garrison, Zev N Kronenberg, Eric T Dawson, Brent S Pedersen, and Pjotr Prins. A spectrum of free software tools for processing the vcf variant call format: vcflib, bio-vcf, cyvcf2, hts-nim and slivar. *PLoS Comput. Biol.*, 18(5):e1009123, 2022.

[17] Glenn Hickey, Jean Monlong, Jana Ebler, Adam M Novak, Jordan M Eizenga, Yan Gao, Tobias Marschall, Heng Li, and Benedict Paten. Pangenome graph construction from genome alignments with Minigraph-Cactus. *Nat. Biotechnol.*, 42:663—673, 2024.

[18] Guillaume Holley, Doruk Beyter, Helga Ingimundardottir, Peter L. Møller, Snædis Kristmundsdottirand, Hannes P. Eggertsson, and Bjarni V. Halldorsson. Ratatosk: hybrid error correction of long reads enables accurate variant calling and assembly. *Genome Biol.*, 22(28), 2020.

[19] Guillaume Holley and Páll Melsted. Bifrost - Highly parallel construction and indexing of colored and compacted de Bruijn graphs. *Genome Biol.*, 21(249), 2020.

[20] Manuel Holtgrewe. Mason–a read simulator for second generation sequencing data. *Technical Report FU Berlin*, 2010.

[21] Neng Huang and Heng Li. compleasm: a faster and more accurate reimplementation of busco. *Bioinformatics*, 39(10):btad595, 2023.

[22] Erich D Jarvis, Giulio Formenti, Arang Rhie, Andrea Guarracino, Chentao Yang, Jonathan Wood, Alan Tracey, Francoise Thibaud-Nissen, Mitchell R Vollger, David Porubsky, et al. Semi-automated assembly of high-quality diploid human reference genomes. *Nature*, 611:519–531, 2022.

[23] Byung June Ko, Chul Lee, Juwan Kim, Arang Rhie, Dong Ahn Yoo, Kerstin Howe, Jonathan Wood, Seoae Cho, Samara Brown, Giulio Formenti, et al. Widespread false gene gains caused by duplication errors in genome assemblies. *Genome Biol.*, 23(205), 2022.

[24] Mikhail Kolmogorov, Kimberley J Billingsley, Mira Mastoras, Melissa Meredith, Jean Monlong, Ryan Lorig-Roach, Mobin Asri, Pilar Alvarez Jerez, Laksh Malik, Ramita Dewan, Xylena Reed, Rylee M Genner, Kensuke Daida, Sairam Behera, Kishwar Shafin, Trevor Pesout, Jeshuwin Prabakaran, Paolo Carnevali, Jianzhi Yang, Arang Rhie, Sonja W Scholz, Bryan J Traynor, Karen H Miga, Miten Jain, Winston Timp, Adam M Phillippy, Mark Chaisson, Fritz J Sedlazeck, Cornelis Blauwendraat, and Benedict Paten. Scalable Nanopore sequencing of human genomes provides a comprehensive view of haplotype-resolved variation and methylation. *Nat. Method*, 20:1483–1492, 2023.

[25] Mikhail Kolmogorov, Jeffrey Yuan, Yu Lin, and Pavel A. Pevzner. Assembly of long, error-prone reads using repeat graphs. *Nat. Biotechnol.*, 37:540–546, 2019.

[26] Peter Krusche, Len Trigg, Paul C Boutros, Christopher E Mason, Francisco M De La Vega, Benjamin L Moore, Mar Gonzalez-Porta, Michael A Eberle, Zivana Tezak, Samir Lababidi, et al. Best practices for benchmarking germline small-variant calls in human genomes. *Nat. Biotechnol.*, 37:555–560, 2019.

[27] Peter Krusche, Len Trigg, Paul C Boutros, Christopher E Mason, Francisco M De La Vega, Benjamin L Moore, Mar Gonzalez-Porta, Michael A Eberle, Zivana Tezak, Samir Lababidi, et al. Best practices for benchmarking germline small-variant calls in human genomes. *Nat. Biotechnol.*, 37:555–560, 2019.

[28] Heng Li. Minimap2: pairwise alignment for nucleotide sequences. *Bioinformatics*, 34(18):3094–3100, 2018.

[29] Heng Li, Jonathan M Bloom, Yossi Farjoun, Mark Fleharty, Laura Gauthier, Benjamin Neale, and Daniel MacArthur. A synthetic-diploid benchmark for accurate variant-calling evaluation. *Nat. Methods*, 15:595–597, 2018.

[30] Heng Li and Richard Durbin. Fast and accurate short read alignment with Burrows–Wheeler transform. *Bioinformatics*, 25(14):1754–1760, 2009.

[31] Heng Li, Xiaowen Feng, and Chong Chu. The design and construction of reference pangenome graphs with minigraph. *Genome Biol.*, 21(265), 2020.

[32] Heng Li, Bob Handsaker, Alec Wysoker, Tim Fennell, Jue Ruan, Nils Homer, Gabor Marth, Goncalo Abecasis, Richard Durbin, and 1000 Genome Project Data Processing Subgroup. The Sequence Alignment/Map format and SAMtools. *Bioinformatics*, 25(16):2078–2079, 2009.

[33] Wen-Wei Liao, Mobin Asri, Jana Ebler, Daniel Doerr, Marina Haukness, Glenn Hickey, Shuangjia Lu, Julian K Lucas, Jean Monlong, Haley J Abel, et al. A draft human pangenome reference. *Nature*, 617:312–324, 2023.

[34] Ann M Mc Cartney, Kishwar Shafin, Michael Alonge, Andrey V Bzikadze, Giulio Formenti, Arkarachai Fungtammasan, Kerstin Howe, Chirag Jain, Sergey Koren, Glennis A Logsdon, Karen H Miga, Alla Mikheenko, Benedict Paten, Alaina Shumate, Daniela C Soto, Ivan Sović, Jonathan M D Wood, Justin M Zook, Adam M Phillippy, and Rhie Arang. Chasing perfection: validation and polishing strategies for telomere-to-telomere genome assemblies. *Nat. Methods*, 19:687–695, 2022.

[35] Benedict Paten, Jordan M Eizenga, Yohei M Rosen, Adam M Novak, Erik Garrison, and Glenn Hickey. Superbubbles, ultrabubbles, and cacti. *J. Comput. Biol.*, 25(7):649–663, 2018.

[36] Edzer Pebesma. Simple Features for R: Standardized Support for Spatial Vector Data. *The R Journal*, 10(1):439–446, 2018.

[37] Victoria E Prince and F Bryan Pickett. Splitting pairs: the diverging fates of duplicated genes. *Nat. Rev. Genet.*, 3:827–837, 2002.

[38] Arang Rhie, Shane A McCarthy, Olivier Fedrigo, Joana Damas, Giulio Formenti, Sergey Koren, Marcela Uliano-Silva, William Chow, Arkarachai Fungtammasan, Juwan Kim, et al. Towards complete and error-free genome assemblies of all vertebrate species. *Nature*, 592:737–746, 2021.

[39] Arang Rhie, Brian P Walenz, Sergey Koren, and Adam M Phillippy. Merqury: reference-free quality, completeness, and phasing assessment for genome assemblies. *Genome Biol.*, 21(245), 2020.

[40] Kishwar Shafin, Trevor Pesout, Pi-Chuan Chang, Maria Nattestad, Alexey Kolesnikov, Sidharth Goel, Gunjan Baid, Mikhail Kolmogorov, Jordan M. Eizenga, Karen H. Miga, Paolo Carnevali, Miten Jain, Andrew Carroll, and Benedict Paten. Haplotype-aware variant calling with PEPPER-Margin-DeepVariant enables high accuracy in nanopore long-reads. *Nat. Methods*, 18:1322–1332, 2021.

[41] Kishwar Shafin, Trevor Pesout, Ryan Lorig-Roach, Marina Haukness, Hugh E Olsen, Colleen Bosworth, Joel Armstrong, Kristof Tigyi, Nicholas Maurer, Sergey Koren, Fritz J Sedlazeck, Tobias Marschall, Simon Mayes, Vania Costa, Justin M Zook, Kelvin J Liu, Duncan Kilburn, Melanie Sorensen, Katy M Munson, Mitchell R Vollger, Jean Monlong, Erik Garrison, Evan E Eichler, Sofie Salama, David Haussler, Richard E Green, Mark Akeson, Adam Phillippy, Karen H Miga, Paolo Carnevali, Miten Jain, and Benedict Paten. Nanopore sequencing and the Shasta toolkit enable efficient de novo assembly of eleven human genomes. *Nat. Biotechnol.*, 38:1044–1053, 2020.

[42] Wei Shen, Shuai Le, Yan Li, and Fuquan Hu. SeqKit: A Cross-Platform and Ultrafast Toolkit for FASTA/Q File Manipulation. *PLoS ONE*, 11(10), 2016.

[43] Felipe A Simão, Robert M Waterhouse, Panagiotis Ioannidis, Evgenia V Kriventseva, and Evgeny M Zdobnov. Busco: assessing genome assembly and annotation completeness with single-copy orthologs. *Bioinformatics*, 31(19):3210–3212, 2015.

[44] Manfred J Sippl. Biological Sequence Analysis: Probabilistic models of proteins and nucleic acids. *Protein Sci.*, 8(3):695–695, 1999.

[45] Jouni Sirén, Jean Monlong, Xian Chang, Adam M Novak, Jordan M Eizenga, Charles Markello, Jonas A Sibbesen, Glenn Hickey, Pi-Chuan Chang, Andrew Carroll, et al. Pangenomics enables genotyping of known structural variants in 5202 diverse genomes. *Science*, 374(6574):abg8871, 2021.

[46] Dominik Stanojević, Dehui Lin, Sergey Nurk, Paola Florez de Sessions, and Mile Šikić. Telomere-to-telomere phased genome assembly using HERRO-corrected simplex Nanopore reads. *bioRxiv*, 2024.

[47] Oxford Nanopore Technologies. Dorado. `https://github.com/nanoporetech/dorado`, 2025. Version 1.1.

[48] Robert Vaser, Ivan Sović, Niranjan Nagarajan, and Mile Šikić. Fast and accurate de novo genome assembly from long uncorrected reads. *Genome Res.*, 27:737–746, 2017.

[49] Bruce J Walker, Thomas Abeel, Terrance Shea, Margaret Priest, Amr Abouelliel, Sharadha Sakthikumar, Christina A Cuomo, Qiandong Zeng, Jennifer Wortman, Sarah K Young, et al. Pilon: an integrated tool for comprehensive microbial variant detection and genome assembly improvement. *PLoS ONE*, 9(11):e112963, 2014.

[50] Jeremy R Wang, James Holt, Leonard McMillan, and Corbin D Jones. FMLRC: Hybrid long read error correction using an FM-index. *BMC Bioinformatics*, 19(50), 2018.

[51] Ryan R Wick and Kathryn E Holt. Polypolish: short-read polishing of long-read bacterial genome assemblies. *PLoS Comput. Biol.*, 18(1):e1009802, 2022.

[52] Ryan R Wick, Louise M Judd, Claire L Gorrie, and Kathryn E Holt. Unicycler: resolving bacterial genome assemblies from short and long sequencing reads. *PLoS Comput. Biol.*, 13(6):e1005595, 2017.

[53] Hadley Wickham. ggplot2. *Wiley interdisciplinary reviews: computational statistics*, 3(2):180–185, 2011.

[54] Zimin, Aleksey V and Marçais, Guillaume and Puiu, Daniela and Roberts, Michael and Salzberg, Steven L and Yorke, James A. The masurca genome assembler. *Bioinformatics*, 29(21):2669–2677, 2013.
