# Supplementary material for: An Icelandic pangenome reference
Source: Nature. 2026 Aug 19;658(8135):421–9. doi: 10.1038/s41586-026-10924-7 (PMC13645673; doi:10.1038/s41586-026-10924-7)
Supplement: Supplementary file 2 [file 41586_2026_10924_MOESM2_ESM.pdf]

nature portfolio

Corresponding author(s): Guillaume Holley
Hannes Eggertsson

Last updated by author(s): Jul 7, 2026

Reporting Summary

Nature Portfolio wishes to improve the reproducibility of the work that we publish. This form provides structure for consistency and transparency in reporting. For further information on Nature Portfolio policies, see our Editorial Policies and the Editorial Policy Checklist.

Statistics

For all statistical analyses, confirm that the following items are present in the figure legend, table legend, main text, or Methods section.

n/a	Confirmed	
☐	☒	The exact sample size (*n*) for each experimental group/condition, given as a discrete number and unit of measurement
☒	☐	A statement on whether measurements were taken from distinct samples or whether the same sample was measured repeatedly
☐	☒	The statistical test(s) used AND whether they are one- or two-sided<br>*Only common tests should be described solely by name; describe more complex techniques in the Methods section.*
☐	☒	A description of all covariates tested
☒	☐	A description of any assumptions or corrections, such as tests of normality and adjustment for multiple comparisons
☒	☐	A full description of the statistical parameters including central tendency (e.g. means) or other basic estimates (e.g. regression coefficient) AND variation (e.g. standard deviation) or associated estimates of uncertainty (e.g. confidence intervals)
☐	☒	For null hypothesis testing, the test statistic (e.g. *F*, *t*, *r*) with confidence intervals, effect sizes, degrees of freedom and *P* value noted<br>*Give P values as exact values whenever suitable.*
☒	☐	For Bayesian analysis, information on the choice of priors and Markov chain Monte Carlo settings
☒	☐	For hierarchical and complex designs, identification of the appropriate level for tests and full reporting of outcomes
☐	☒	Estimates of effect sizes (e.g. Cohen's *d*, Pearson's *r*), indicating how they were calculated

Our web collection on statistics for biologists contains articles on many of the points above.

Software and code

Policy information about availability of computer code

Data collection	No software was used for data collection.
Data analysis	The newly developed software for the study are Emblask v0.1 (https://github.com/DecodeGenetics/emblask), Weaver v0.2.0 (https://github.com/DecodeGenetics/weaver), and Weaver's Nextflow pipeline v1.0 (https://github.com/DecodeGenetics/nf-weaver). All public software used are listed in Supplementary Methods 14. They are:<br>BWA v0.7.16a, hifiasm v0.16.0, yak v0.1-r69, compleasm v0.2.1, minimap2 v2.24, dipcall v0.3, bcftools v1.18, samtools v1.18, Bifrost v1.2.1, Flagger v0.1, rtg tools v3.10.1, vcfdist v2.3.2, Mason2 v2.0.9, minigraph v0.20-r559, vcfwave v1.0.9, vcfbub v0.1.0, SeqKit v2.0, Merqury v1.3, GraphTyper v2.7.7, DeepVariant v1.6.0 (prerelease), Crumble v0.9.1, vg v1.51.0, Shasta v0.14.0, HapDup v0.12.0, Dorado v1.1.1

For manuscripts utilizing custom algorithms or software that are central to the research but not yet described in published literature, software must be made available to editors and reviewers. We strongly encourage code deposition in a community repository (e.g. GitHub). See the Nature Portfolio guidelines for submitting code & software for further information.

Data

Policy information about availability of data

All manuscripts must include a data availability statement. This statement should provide the following information, where applicable:

- Accession codes, unique identifiers, or web links for publicly available datasets
- A description of any restrictions on data availability
- For clinical datasets or third party data, please ensure that the statement adheres to our policy

HPRC-ICE pangenome reference (GFA, VCF and the Weaver index) is accessible at DOI:10.5281/zenodo.19609698
Singularity containers for Weaver, Emblask and DeepVariant for using the weaver trained model are accessible at DOI:10.5281/zenodo.21240459

Access to the raw Icelandic sequence data cannot be made publicly available because Icelandic law and the regulations of the Icelandic Data Protection Authority prohibit the release of individual-level and personally identifying data. Data access can be granted only at the facilities of deCODE genetics in Iceland, subject to Icelandic law regarding data usage. Anyone wishing to gain access to the data should contact D.F.G. (dgudbjar@amgen.com). Icelandic law allows for unimpeded sharing of summary-level data.

Other accessed data:
The human reference genome GRCh38/hg38 was obtained from the Genome Reference Consortium/NCBI Assembly database, RefSeq accession GCF_000001405.26 and GenBank accession GCA_000001405.15.
HPRC graph: https://github.com/human-pangenomics/hpp_pangenome_resources
GA4GH/T2T/GIAB stratification regions: https://ftp-trace.ncbi.nlm.nih.gov/ReferenceSamples/giab/release/genome-stratifications/v3.3/GRCh38@all/
GIAB T2T-HG002 Q100 small variant benchmark: https://ftp-trace.ncbi.nlm.nih.gov/ReferenceSamples/giab/data/AshkenazimTrio/analysis/NIST_HG002_DraftBenchmark_defrabbV0.018-20240716/
GIAB HG002 short reads: https://ftp-trace.ncbi.nlm.nih.gov/ReferenceSamples/giab/data/AshkenazimTrio/HG002_NA24385_son/NIST_HiSeq_HG002_Homogeneity-10953946/NHGRI_Illumina300X_AJtrio_novoalign_bams/HG002.GRCh38.60x.1.bam
GIAB HG003 short reads https://ftp-trace.ncbi.nlm.nih.gov/ReferenceSamples/giab/data/AshkenazimTrio/HG003_NA24149_father/NIST_HiSeq_HG003_Homogeneity-12389378/NHGRI_Illumina300X_AJtrio_novoalign_bams//HG003.GRCh38.60x.1.bam}
GIAB HG004 short reads https://ftp-trace.ncbi.nlm.nih.gov/ReferenceSamples/giab/data/AshkenazimTrio/HG004_NA24143_mother/NIST_HiSeq_HG004_Homogeneity-14572558/NHGRI_Illumina300X_AJtrio_novoalign_bams/HG004.GRCh38.60x.1.bam
CARD HG002 ONT R9.4 assembly: https://s3-us-west-2.amazonaws.com/human-pangenomics/index.html?prefix=publications/Napu_paper_ONT_Coriell_SingleFC_2023/HG002_R9/assembly/
HPRC assemblies: https://github.com/human-pangenomics/HPP_Year1_Assemblies
HPRC GRCh38 Minigraph-Cactus https://s3-us-west-2.amazonaws.com/human-pangenomics/index.html?prefix=pangenomes/freeze/freeze1/minigraph-cactus/
Clinvar variants (VCF): https://ftp.ncbi.nlm.nih.gov/pub/clinvar/vcf_GRCh38/archive_2.0/2025/clinvar_20250209.vcf.gz
Ensembl genes (v112) https://ftp.ensembl.org/pub/release-112/gtf/homo_sapiens/Homo_sapiens.GRCh38.112.gtf.gz

Research involving human participants, their data, or biological material

Policy information about studies with human participants or human data. See also policy information about sex, gender (identity/presentation), and sexual orientation and race, ethnicity and racism.

Reporting on sex and gender	Neither sex nor gender were considered in the study design.
Reporting on race, ethnicity, or other socially relevant groupings	No reporting on any of these social groupings is performed.
Population characteristics	The human study participants are Icelandic individuals, largely healthy individuals recruited through various research projects. Data acquisition and processing have been described previously in detail Jonsson, H. et al. "Whole genome characterization of sequence diversity of 15,220 Icelanders", Scientific Data, (4) 170115, 2017. Age and sex of the individuals were used as covariates in all genotype imputations and phenotype associations. No diagnosis status or treatment information were used as covariate.
Recruitment	Individuals were collected as part of various disease assocation studies at deCODE genetics.
Ethics oversight	All participants were Icelanders who signed an informed consent form and donated biological samples for genotyping as part of various research projects at deCODE genetics approved by the National Bioethics Committee (NBC) in Iceland. Processing of data was performed in agreement with the approvals issued by the National Bioethics Committee (NBC) and conditions set by the Data Protection Authority (DPA) (ref. PV_2017060950ÞS) on procedures to ensure security in the processing of personal data for the scientific research within the health sector conducted by deCODE genetics and the Act on Scientific Research in the Health Sector No. 44/2014.

Note that full information on the approval of the study protocol must also be provided in the manuscript.

Field-specific reporting

Please select the one below that is the best fit for your research. If you are not sure, read the appropriate sections before making your selection.

☒ Life sciences ☐ Behavioural & social sciences ☐ Ecological, evolutionary & environmental sciences

Life sciences study design

All studies must disclose on these points even when the disclosure is negative.

Sample size	No sample-size calculation was performed. All eligible samples (n = 57,630) passing described quality filters were included to maximize statistical power and avoid selection bias. Sample size was decided based on available sequencing budget.
Data exclusions	No data were excluded from the analysis among samples passing the quality filters.
Replication	The main experimental findings were performed using Nextflow pipelines in Singularity containers (DOI:10.5281/zenodo.21240459), ensuring reproducibility of those experiments. We replicated our variant association to Parkinson's disease in one other external cohort, the UK BioBank. There were no unsuccessful attempts at replication.
Randomization	No group allocation was performed.
Blinding	No group allocation was performed so blinding was not relevant for this study. Investigators were not involved with data collection of raw data.

Reporting for specific materials, systems and methods

We require information from authors about some types of materials, experimental systems and methods used in many studies. Here, indicate whether each material, system or method listed is relevant to your study. If you are not sure if a list item applies to your research, read the appropriate section before selecting a response.

Materials & experimental systems

n/a	Involved in the study	
☒	☐	Antibodies
☒	☐	Eukaryotic cell lines
☒	☐	Palaeontology and archaeology
☒	☐	Animals and other organisms
☒	☐	Clinical data
☒	☐	Dual use research of concern
☒	☐	Plants

Methods

n/a	Involved in the study	
☒	☐	ChIP-seq
☒	☐	Flow cytometry
☒	☐	MRI-based neuroimaging

Plants

Seed stocks	*Report on the source of all seed stocks or other plant material used. If applicable, state the seed stock centre and catalogue number. If plant specimens were collected from the field, describe the collection location, date and sampling procedures.*
Novel plant genotypes	*Describe the methods by which all novel plant genotypes were produced. This includes those generated by transgenic approaches, gene editing, chemical/radiation-based mutagenesis and hybridization. For transgenic lines, describe the transformation method, the number of independent lines analyzed and the generation upon which experiments were performed. For gene-edited lines, describe the editor used, the endogenous sequence targeted for editing, the targeting guide RNA sequence (if applicable) and how the editor was applied.*
Authentication	*Describe any authentication procedures for each seed stock used or novel genotype generated. Describe any experiments used to assess the effect of a mutation and, where applicable, how potential secondary effects (e.g. second site T-DNA insertions, mosiacism, off-target gene editing) were examined.*
