# Supplementary material for: An Icelandic pangenome reference
Source: Nature. 2026 Aug 19;658(8135):421–9. doi: 10.1038/s41586-026-10924-7 (PMC13645673; doi:10.1038/s41586-026-10924-7)
Supplement: Supplementary file 3 [file 41586_2026_10924_MOESM3_ESM.pdf]

nature portfolio

Peer Review File

An Icelandic pangenome reference

Corresponding Author: Dr Hannes Eggertsson

Any redactions in this file are there to maintain patient confidentiality, the confidentiality of unpublished data, or to remove third-party material.

This file contains all reviewer reports in order by version, followed by all author rebuttals in order by version.

Version 0:

Reviewer comments:

Referee #1

(Remarks to the Author)
Holly and colleagues have developed new genome assembly, pan-genome graph construction, read mapping and variant detection methods and applied these to both existing human resequencing data (biobank) and a new set of resequencing and ONT data from Icelandic-origin samples. The novel methods appear to offer some improvements over existing software (but see below), the new reference genomes are likely of reasonably high quality (but see below), and the discovery of previously undetected variants with potential clinical applications is interesting from a biological and potentially computational perspective.

Overall, I very much enjoyed the biological aspects of this paper, but I have one general gripe: throughout I had trouble detangling the lines of logic supporting method benchmarking and novel biological discovery. I think simply splitting out the analyses so that method validation (benchmarking) is always done for all new methods and existing methods with *exactly* the same input data, then biological analysis and interpretation is done using only the optimal method.

Below, I outline a number of comments that I hope will help improve future drafts.

- John T. Lovell (11-Apr 2025)

Major comments

#####################
Genome assembly quality:
The choice of low accuracy ONT reads appears to be the major motivation for developing a novel assembly tool (see below). I was surprised to see that a genome wide base quality of Q60 in these assemblies. I would like to see more evidence that this per base quality is actually achieved (before not just being surprised, but also impressed), especially highlighting both regions that can be polished with short reads and those that are too repetitive for accurate illumina reads to uniquely map. This analysis should be relatively straightforward if there is ONT data sitting around for one of the HPRC genotypes, or just simulating 7% error rate ONT reads from the outbred HG002, re-assembling with Emblask, and counting the number of mismatches (see comment above about benchmarking). I ask for this because I can envision a situation where the reported higher numbers of variants detected in the pangenome (especially short INDELs) is due to unresolved ONT sequencing errors, and better unique mapping of short reads could be due to random variance in repetitive regions across the pangenome that reduces the number of exactly duplicated mappings.

#####################
Weaver and read mapping and variant detection improvements:
Throughout, the authors claim that the new Weaver software better identifies variants than existing software. I am very amenable to this idea, but I am having trouble finding compelling evidence of this reported result. For example:
(1) lines 63-65 highlights improvements from linear to reference detection (and not comparison to variant detection from existing graph-based software);
(2) other analyses show comparisons where methods and datasets are not reciprocally investigated so it is impossible to tell

whether the improvements are due to using new resequencing data (lines 115-117; 207-219), different pangenome graphs (lines 144-151; and see next section of comments), different sets of individuals (220-229: even though there are fewer individuals in the Weaver set, n samples need not have bearing on the number of variants expected), genotyping error rate via comparison of monozygotic twins, which would be a great benchmark, is only shown for one method (195-197);
(3) lines 142-143 and figure 4a-b are interpreted as showing significant improvement of Weaver relative to comparable software; however, variance associated with the very similar profiles does not indicate to me that there is much of a difference and the sample size n = 1 needs to be increased to at least 2;
(4) lines 160-174 purportedly report improved mapping with Weaver-DeepVariant; however, this approach only is the most accurate in low-quality regions which may or may not represent an overall improvement in variant detection.
(5) some comparisons (e.g. 243-245) test Weaver against linear single-reference mapping. In these cases, it's impossible to tell whether the authors are providing evidence of the value of a graph per se or the graph construction/mapping method.

#####################
Comparisons to existing graph-based methods:
The new approaches described in this manuscript may offer significant gains over existing graph-based methods, especially with respect to identifying rare variants in genomic regions that are difficult to characterize. However, the way existing graph methods were applied does not necessarily reflect current best-practices for those workflows (i.e., lines 110-112). Specifically, when comparing Weaver against Giraffe and DRAGEN, an allele frequency-filtered version of the HPRC graph was used. A more appropriate comparison might be to use a personalized pangenome approach to subsample graph haplotypes, construct a sample-specific subgraph, and then align reads to haplotypes that could already contain rare variants (Sirén et al. 2024, doi:10.1038/s41592-024-02407-2).

#####################
Comparison to "personalized pan-genomes"
Ideally, Weaver would have been compared against an application of this personalized approach to a graph comprising the HPRC genomes plus the ICE haplotypes. Attempting to construct a minigraph/Minigraph-Cactus graph for even a single chromosome with all haplotypes would be a heavy lift, computationally, and potentially an additional clear and convincing argument for Weaver over existing methods that require full graph construction prior to subsampling. If such a test build is computationally prohibitive, the rationale for not applying existing personalized pangenome methods could at least be clearly addressed in the manuscript, especially given the emphasis on detection of rare variants that would be specifically screened by the existing graph allele frequency-filtering approaches referenced in the manuscript.

I do think the idea of partitioning SVs in an rGFA and small variants in a VCF is clever and potentially a great solution to the problem of haplotype collection outpacing the computational capability to compile all available haplotypes into a single data structure prior to relevant subsampling.

#####################
Minor comments

It is not clear how small variants nested within structural variants are handled in the Weaver workflow. It seems as though these variants are not considered, given lines 128-129 ("...pangenome construction can scale to hundreds of human haplotypes if restricted to non-nested small variants."), but this should be reiterated somewhere in the Methods.

I had trouble understanding why graphTyper and not the weaver-DeepVariant (which was the benchmarked method) was used to call variants in the biobank data (line 181).

There is not much reference to how the Y chromosome was handled in the assemblies or variant detection. Assuming it was fully assembled and included, but this needs to be more precisely described since it represents a challenge to all bioinformatic aspects of this project.

How were the small variant calls for the ICE haplotypes integrated into the Weaver reference set? Were the coordinates for those calls retained in the specific coordinate systems of their respective dual assemblies and annotated with respect to those rGFA nodes in a VCF or were they somehow converted to the GRCh38 reference coordinate system prior to indexing?

I think it could be made more clear throughout that all analyses in this paper are based on short variants. I think when folks read "pangenome," they think "all variants." Which are definitely present in the haplotypes they contribute, but that's not what this paper is about.

(Remarks on code availability)

Referee #2

(Remarks to the Author)
I co-reviewed this manuscript with one of the reviewers who provided the listed reports.

(Remarks on code availability)

Referee #3

(Remarks to the Author)
Review: Icelandic Pangenome

This paper presents the "Icelandic Pangenome", an extension of the HPRC pangenome to include several hundred haplotypes from the Icelandic population ascertained from long-read data. The paper uses this pangenome to ascertain small variants using short reads from tens of thousands of Icelanders. The authors demonstrate an increase in the yield of variants relative to using a standard linear reference and investigate two medically relevant variants that were only ascertained using the pangenome approach. The paper contains a very extensive amount of new methods work, principally in developing the Emblask de novo assembly pipeline for ONT sequencing as well as (and IMO more significantly), in developing a new short-read to pangenome mapping algorithm, Weaver. The paper clearly represents a large body of work by a team of field leaders and is impressive in its scope, it is also exciting that they demonstrate some concrete improvements in variant ascertainment leading to biomedically interesting results.

My major concerns/criticisms are as follows:

Emblask pipeline

(1) The Emblask pipeline/assembler is well described and contains some novel innovations. However, for a journal like Nature I question the value of spending a substantial amount of time describing this, given that:

(1A) It is really aimed at a previous generation of Oxford Nanopore Sequencing (R9.4), which is largely historical at this point because it exhibits a much higher error rate than contemporary ONT R10 which supersedes it in all ways and which has been widely available for more than a year. It is not as if the cohort of samples sequenced with R9 is that large - there are other studies already available that show larger such cohorts.

(1B) While the Emblask method is interesting and well done, there has been a lot of innovation in assembly algorithms and the only real comparison is to a Shasta/HapDup assembly, a comparison which is buried in the supplementary methods and shows a good improvement in the indel accuracy but not much more. If this is to be published in a high-profile journal I would suggest shrinking the text dedicated to it and definitely including a comparison to the latest ONT error correction methods - I have personally seen quite good assemblies for R9 from Hifiasm with its latest built-in error correction, as well when using Herro plus either Hifiasm, Verkko or the latest "Mode 3" from Shasta.

(1C) Finally, overall, the Emblask pipeline has a distinctly "not invented here" feel. The authors, for example, invent their own error correction methods and wholly omit discussion of the latest assembly innovations (which are pertinent even for these R9.4 reads) from other groups.

Major Technical Nits

(2) (This also relates to the value of the Emblask pipeline here - so also consider in the point above) Despite putting a huge amount of effort into assembling the genomes, the authors don't actually appear to use the structural variations that are inferred (Supplementary Figure 12). Rather they go through a process of mapping the assemblies back to a reference and then inferring small variants from them. I think it is natural to ask if it would have been more straightforward to simply use the ONT data with PMDV/Clair to directly call small variants for the ONT data. I presume the authors did intend to use the SVs from the assemblies but at some point gave up. Regardless, this seems like a major missed opportunity that leaves you scratching your head and needs some admission/discussion. I would minimally like to see evidence that the small variants discovered using Emblask are better than using the more direct approach of reference-based variant calling (PMDV will do phasing). If there are benefits to the Emblask pipeline for this use case over that approach I think the authors should avoid obfuscation and make it easier to understand (I read the supplementary methods in full and am still not sure!).

(3) The text describes using rGFA for the pangenome, and the supplementary methods give an overview of the model. rGFA is exciting in that it provides a way to define non-reference coordinates. However, as far as I can tell, the additional variation added to the graph from the ICE samples does not encompass these non-reference sequences (variants are only added on the reference). Further, from what I can tell, there is no attempt to use Graphtyper to call variants against these additional non-reference sequences. This feels like a major missed opportunity to demonstrate calling variants within non-reference, commonly polymorphic sequence. There is a discussion about non-primary GRCh38 contigs, but I think this is talking about contigs in GRCh38 that are not on the primary reference. I think the authors should either remove the rGFA discussion and make it clearer about the limitations of the current approach (being limited to small variants on the primary reference - as is true of other pipelines too (to be clear)), or attempt to call and quantify small variants in this expanded coordinate system (which would make things a lot more significant in my view).

(4) The paper discusses how other pangenome pipelines filter variants and that Weaver does not. The paper says: "Because rare variants may have a large impact on the regulation of gene expression with significant consequences in clinical outcomes and disease studies, we show that including them in our mapping model enabled the discovery of pathogenic variants associating with disease in Iceland and in the UK." However, it is not clear to me that the results shown are actually due to this inclusion. Further, it is surprising that mapping is not adversely affected by rare variants - as the

authors no doubt know, mostly rare variants have been excluded precisely because they do impact mapping accuracy. Some analysis and demonstration of these claims would really help.

Concerns around the increased yield of "confident" variants

(5) The authors do a pretty reasonable job of benchmarking their Weaver/Graphtyper pipeline against single samples (It is interesting that in the only benchmark that is widely compared against (the HG002 T2T one) the Dragen pipeline appears better and the vg/DeepVariant pipeline is about the same.) However, what the authors do not really convincingly do is show that the increase in "reliable variants" ascertained by calling against the Icelandic populations is not a statistical artifact of differences between the Weaver/Graphtyper pipeline and the BWA-MEM/Graphtype based pipeline. The 6% of additional "reliable" variants might be due to differences in parameterization, and indeed might be an overestimate of the true difference because these additional variants are not validated in a reasonable way. The authors could show the calling of these additional variants using their held-out pacbio data, but do not. So, while I think the reported result is promising, it really needs more careful validation before I'm ready to accept it - and certainly more controls are needed for publication here.

(6) The authors compare Weaver-Graphtyper to BWA-MEM/Graphtyper, but it would also be useful to know if other, existing pipelines would call the GBA1 variants. It says that Dragen does not. Does vg/DV? Does BWA-MEM/DV? Again, this is an important claim and it should be carefully interrogated.

Minor points:

There are some missing (??) figure references in the supplementary material. Looks like a latex issue.
The authors discuss pangenome personalization in their discussion as a future direction. However, they don't attempt to use it when comparing to vg - it would be good to update this. That pipeline has been available and published for around a year.

I struggled with this description: "As a minimizer might span multiple segments, Weaver requires k to be an odd number in order to index each minimizer using the position of the base at its center. This guarantees that minimizer occurrences are always indexed with respect to a single segment of the graph. It also prevents to query the graph for minimizers in both directions as the orientation of the read sequence compared to the sequences in the graph is unknown." Are you just saying you avoid palindromes by using an odd-length minimizer?

The version of some tools used is pretty old and some results might be a little out of date as a result.

(Remarks on code availability)

Referee #4

(Remarks to the Author)
Holley, Eggertsson et al present a manuscript that describes two software tools and methods to enable pangenome graphs construction and mapping at biobank scale: Emblask and Weaver. They apply the methods to data from several datasets from Iceland and the UKBiobank. As a proof of concept, they find SNPs in genes known to cause severe disorders.

The advances are mainly technical. I don't think the results are of particularly broad interest. I found the manuscript to be descriptive, but missing a discussion of many key questions, which I ask about below. I am concerned about the apparent bias in genotyping visible in the allele frequency distribution in Figure 5. The results about discovering SNPs in known pathogenic genes are a good proof of concept of the approach, but I wonder how much more discovery there is to be had besides them.

I think that these methods will be useful for people who are building pangenome graphs and mapping to them, including those working in species besides human. I think that allowing VCF files to be used will enhance usability of pangenome graphs. The software appears to run, although I did not find a detailed walk-through/tutorial.

As far as I can tell the manuscript appropriately references prior work. The methods section was appropriately detailed. Some of the treatment of uncertainty was inadequate (see specific comment below).

Major concerns
- Figure 5: allele frequency spectrum for SNPs has a massive uptick at 50% -- why? I believe this is an artifact of something in the author's pipeline.
- Figure 5: could the authors explain the periodic behavior of the indels?
- Line 216: why is this comparison across a different set of samples? I am interested in comparing the number of variants called across the same set of samples. If the samples differ, it's harder to interpret the difference.
- Line 231: here again, the sample sizes differ, making it hard to compare the number of variants called.
- Line 219: what is the comparison at dark regions, as previously mentioned?
- Line 168: Some of these differences are quite small. Are the differences in F1 score significant across the methods?

Minor concerns
- Line 79: the text would benefit from clarifying what polishing is.
- Line 107: Does the rate of increases differ among variants?
- Line 109: Are singleton variants in SNPs and indels found in different parts of the genome?
- Line 119-120: Do these results refer to a specific display item?
- Line 147: how is mappability measured here?
- Line 162: what is the allele frequency distribution of the safe variants? are common alleles more likely to be safe?
- Figure 2 legend: A description of how completeness and quality are measured in the legend would be helpful.
- Figure 2d: Can you use this curve to estimate when the number of small variants will saturate?
- Figure 4d: is it correct to say that the runtime complexity (i.e. O(n)) with respect to depth of all three methods is the same, but Weaver has a smaller constant?

(Remarks on code availability)
Code provided shows a sample build and run of Weaver. There is no README, but there is an bash script that builds and runs Weaver. I think having a detailed tutorial would be helpful for users.

Version 1:

Reviewer comments:

Referee #1

(Remarks to the Author)
The authors have provided some updates to their manuscript and detailed explanations in the response to reviewers. Overall, I think the manuscript is somewhat improved; however, all of the general concerns I raised in the previous review (and many of the concerns raised by other reviewers) remain.

In many places, the authors provide sufficient responses to the issues raised by reviewers in the rebuttal document, but they do not address these issues in the text (not just my comments but this is true of the other reviewers' as well). This is not sufficient - we are asking about these things because they need to be clarified in the manuscript. If you believe that it is not important enough to change in the main text, then that choice needs to be defended in the rebuttal.

Throughout, I still had trouble distinguishing between whether the results are about performance of the methods or new biological discoveries. As before, the ground truth methods are confusing as well. For example, Figure4 provides both methodological comparisons between equivalent methods (graph alignment method comparisons 4a-b) and non-apples-to-apples comparisons related to the approach in general (e.g. single reference issues 4c).

Also, I don't find the reasoning that it is computationally challenging to to be a persuasive argument to not provide a comparison to a baseline within identical input data for each method (this is provided as a reason to not do several comparisons). New software requires such comparisons, and the improvements need to be stated clearly. This is especially important here, as the lead contribution of this work is a new assembler. Perhaps just use smaller datasets and compare those directly?

Other less critical stuff that was not addressed sufficiently in the revised manuscript:

The improvements of the new methods are not obvious when compared to GiraffePPR (outside of computational time, e.g. 4d); the specific places where improvements are made should be clear. In particular, GiraffePPR could be expected to offer significant improvements in calling rare variants over the frequency-filtered approach.

At several points you state that a big improvement of this method is the handling of complex nested variants; but you also state in the response to reviews that "we think including small variants within SVs would likely offer little value to this work"; this is an important sentiment that was not conveyed in the manuscript. In the presented workflow, such variants are situated on non-reference contigs and functionally invisible to downstream analytical steps. Please add this text into the manuscript with some descriptions of why it is so; that way, the reader can contextualize your decisions.

(Remarks on code availability)

Referee #2

(Remarks to the Author)
I co-reviewed this manuscript with one of the reviewers who provided the listed reports.

(Remarks on code availability)

Referee #3

(Remarks to the Author)
Thanks - the authors have provided reasonable responses to all my queries and quibbles, although much of it has not changed the overall shape of the manuscript. However, I still have concerns about the significance of the developed methods:

(a) the rather out of date nature of the Icelandic long-read sequencing data and knock-on value of the Emblask pipeline,
(b) the relatively marginal improvements in variant calling results achieved as viewed through the lens of GRCh38 projection (it feels like diminishing returns because everything is ultimately forced into GRCh38 space),
(c) the lack of value achieved by ignoring the SVs in the Icelandic assemblies (which again makes me really question the value of the Emblask pipeline as presented - I stand by my comment that they could have just used this data to do variant calling against 38 and added it the pangenome VCF),
and (d) the focus only on variants mappable to GRCh38.

While I think the paper is technically sound and definitely very interesting from my perspective as a methods geek, it does feel of questionable relevance to a broader, less methods focused audience.

(Remarks on code availability)

Referee #4

(Remarks to the Author)
The authors have addressed my comments.

(Remarks on code availability)
The authors have addressed my comments.

Version 2:

Reviewer comments:

Referee #1

(Remarks to the Author)
The authors have done a nice job of addressing our previous gripes. We think the methodological aspects of the article are much improved — this will be a very nice contribution! Just one minor point: caption for Figure 4C should include how values were calculated (e.g., are these the total lengths of regions not covered by short reads from any of the 19 libraries?). Nothing else from us.

(Remarks on code availability)

Referee #2

(Remarks to the Author)
I co-reviewed this manuscript with one of the reviewers who provided the listed reports.

(Remarks on code availability)

Referee #3

(Remarks to the Author)
Thanks, I think the paper is stronger for the changes made. I also continue to appreciate the level of technical development and ambition, particularly with respect to the Weaver mapper, which looks like a useful, well architected advance. I really want to leave this review at that - really I do, however, I still think the paper, as it stands, continues to have major issues: (1) it is unfortunately selectively presenting somewhat marginal improvements, (2) it continues to focus on a now archaic (in terms of sequence technology) assembly pipeline that results in very small downstream improvements for genotyping, and (3) doesn't actually make any of the data (even in derived, de-identified form) available.

In terms of the first issue, selective presentation, I would say:

- The authors are using very old versions of software to compare against, particularly the Dragon software they use (the version is only given in the supplement!) is from July 2023. I am aware that subsequent versions have substantially

improved performance since that time. Similarly, I know versions of the DeepVariant for Giraffe have been steadily improving. It may be a chore, but it is not reasonable to compare to these very old software at this point, and I think things would look quite different compared to the latest Dragen.

- In Figure 4 and the supplement, the authors only show relative to the HG002 T2T benchmark in the low-confidence regions? This seems crazy - firstly, the high-confidence fraction of the assembly is the large majority (>90%) of the genome containing the large majority of variants, secondly, the variants in the low confidence fraction are, by design, much more suspect and more likely to be wrong. Figure 1 needs to be changed to show the HG002 benchmark high-confidence (and low and all-) benchmark results. Similarly, a complete set of results needs to be in the supplement. The fact that the current version is worse than the three year old version of Dragen on the high-confidence regions and tied with old versions of Giraffe is very concerning.

- The mapping “dark regions of GRCh38” is not a reasonable way of assessing accuracy because we do not know if the reads mapped in these regions are reliably mapped, and no attempt is made to check this. The simulated mapping results (which were created by the authors) show that the actual differences in performance of the mappers are relatively small. I think all the dark region analysis is showing is differences in the way the mappers give up in mapping to highly repetitive sequences according to minimizer repetition thresholds.

- The authors spend a lot of time comparing to Dragon and Giraffe using DV as their genotyping tool for Weaver, but once they shift to genotyping across the Icelandinc cohort they switch to their in house genotyper. This is justified as being necessary given the computational cost of DV, but if that is the case they MUST include weaver/graphtyper results in Figure 4 and the supplement. Presumably they don’t, because they know it will be weaker than Dragen et al.

In terms of the second issue, overall marginal improvements, as the authors acknowledge the Emblask pipeline presented is for a sequencing technology that is now (long!) discontinued. I stressed this is my earlier reviews, but the advice to reduce this does not seem to have been taken. Further, all of the work done with the assemblies results in a very slightly improved set of small variants to feed to Weaver for the HPRC-ICE pangenome. Nothing continues to be done with the SVs. However, as Supplementary Figure 17 shows (again, for some reason only in the low-confidence regions - this is not justifiable, btw) the improvement in variant calling accuracy is negligible to non-existent with the addition of all the Icelandic data (btw, that result should be in Figure 4!). No attempt is made to show that the ICE portion improves variant ascertainment in the Icelandic population, or really anything else that would convince you that HPRC-ICE is better than HPRC. As such, half the paper CONTINUES to feel like mostly a waste of time, particularly given that the assemblies will never be released for privacy reasons.

Finally, in terms of data release and software- the authors should release the data and software instead of saying they will release it. The github link for Weaver is empty, and the code ocean link provided is for testing with a toy region.

(Remarks on code availability)

Version 3:

Reviewer comments:

Referee #1

(Remarks to the Author)
The authors have addressed our previous concerns. For this review, we are focusing only on comments and revisions related to issues relayed by reviewer #3 in the last round. Overall, we are not concerned at all about the use of somewhat older sequencing technology and bioinformatics tools in this work. This is how time works, and just because something was done in the past does not mean it is not useful. We want to highlight again here: to us, the main point of this ms is the biological/clinical knowledge gain of better variant detection, not necessary massive methods advances.

Below, the previous comment from reviewer #3 is quoted, and our responses to this follow in a paragraph flagged with **.

“[The manuscript] is unfortunately selectively presenting somewhat marginal improvements, (2) it continues to focus on a now archaic (in terms of sequence technology) assembly pipeline that results in very small downstream improvements for genotyping, and (3) doesn’t actually make any of the data (even in derived, de-identified form) available.”

** Authors’ responses to this comment are appropriate. More specific comment responses are addressed below.

“The authors are using very old versions of software to compare against, particularly the Dragon software they use (the version is only given in the supplement!) is from July 2023. I am aware that subsequent versions have substantially improved performance
since that time. Similarly, I know versions of the DeepVariant for Giraffe have been steadily improving. It may be a chore, but it is not reasonable to compare to these very old software at this point, and I think things would look quite different compared to the latest Dragen”

** In their rebuttal, the authors state “We have now explicitly stated in the manuscript that we chose the latest published versions for our benchmarks (line 154)”. This is true, however, it is usually required that the authors also include the actual versions/releases of the software they used. I assume this will be resolved during the checklist process, so no concern from us.

“In Figure 4 and the supplement, the authors only show relative to the HG002 T2T benchmark in the low-confidence regions? This seems crazy - firstly, the high-confidence fraction of the assembly is the large majority (>90%) of the genome containing the large majority of variants, secondly, the variants in the low confidence fraction are, by design, much more suspect and more likely to be wrong. Figure 1 needs to be changed to show the HG002 benchmark high-confidence (and low and all-) benchmark results. Similarly, a complete set of results needs to be in the supplement. The fact that the current version is worse than the three year old version of Dragen on the high-confidence regions and tied with old versions of Giraffe is very concerning.”

** The authors address this comment by adjusting Fig. 4 and some associated text. While we don’t fully agree with reviewer #3’s reason or tone, there is a bit of a point - even though the vast majority of pangenome improvements should be in hard-to-call regions, the effects of bad variant calling in challenging parts of the genome can sometimes bleed over into the single-copy regions. So, it’s probably a good idea to show whole-genome statistics. That said, we were also fine with the previous presentation. Regardless, we feel like the changes to the text and the responses to reviewer #3 in their first comment are fine and no additional adjustments need to be made.

“The mapping “dark regions of GRCh38” is not a reasonable way of assessing accuracy because we do not know if the reads mapped in these regions are reliably mapped, and no attempt is made to check this. The simulated mapping results (which were created by the authors) show that the actual differences in performance of the mappers are relatively small. I think all the dark region analysis is showing is differences in the way the mappers give up in mapping to highly repetitive sequences according to minimizer repetition thresholds.”

** Author responses to this are fine. The addition of that one sentence doesn’t add much but does address this comment to some extent.

“The authors spend a lot of time comparing to Dragon and Giraffe using DV as their genotyping tool for Weaver, but once they shift to genotyping across the Icelandinc cohort they switch to their in house genotyper. This is justified as being necessary given the computational cost of DV, but if that is the case they MUST include weaver/graphtyper results in Figure 4 and the supplement. Presumably they don’t, because they know it will be weaker than Dragen et al.”

** The authors have added some clarification to the text related to this comment. These changes are appropriate and help with communication.

“In terms of the second issue, overall marginal improvements, as the authors acknowledge the Emblask pipeline presented is for a sequencing technology that is now (long!) discontinued. I stressed this is my earlier reviews, but the advice to reduce this does not seem to have been taken. Further, all of the work done with the assemblies results in a very slightly improved set of small variants to feed to Weaver for the HPRC-ICE pangenome. Nothing continues to be done with the SVs. However, as Supplementary Figure 17 shows (again, for some reason only in the low-confidence regions - this is not justifiable, btw) the improvement in variant calling accuracy is negligible to non-existent with the addition of all the Icelandic data (btw, that result should be in Figure 4!). No attempt is made to show that the ICE portion improves variant ascertainment in the Icelandic population, or really anything else that would convince you that HPRC-ICE is better than HPRC. As such, half the paper CONTINUES to feel like mostly a waste of time, particularly given that the assemblies will never be released for privacy reasons.”

** The modest changes to the text are in line with how we would have approached this comment. There really isn’t much to be done about old technology, and it’s a bit of a bummer this reviewer latched on to that. The data is there and still needs to be analyzed. As for the SVs, we think the treatment for those and the SI fig are good additions.

“Finally, in terms of data release and software- the authors should release the data and software instead of saying they will release it. The github link for Weaver is empty, and the code ocean link provided is for testing with a toy region.”

** Response here is good, but please ensure this information makes it into the final manuscript and not just the rebuttal.

(Remarks on code availability)

Referee #2

(Remarks to the Author)
I co-reviewed this manuscript with one of the reviewers who provided the listed reports.

(Remarks on code availability)
Code for all new programs is publicly available, as are data from the manuscript.

Open Access This Peer Review File is licensed under a Creative Commons Attribution 4.0 International License, which permits use, sharing, adaptation, distribution and reproduction in any medium or format, as long as you give appropriate credit to the original author(s) and the source, provide a link to the Creative Commons license, and indicate if changes were made.
In cases where reviewers are anonymous, credit should be given to 'Anonymous Referee' and the source.
The images or other third party material in this Peer Review File are included in the article's Creative Commons license, unless indicated otherwise in a credit line to the material. If material is not included in the article's Creative Commons license and your intended use is not permitted by statutory regulation or exceeds the permitted use, you will need to obtain permission directly from the copyright holder.
To view a copy of this license, visit https://creativecommons.org/licenses/by/4.0/

Referee #1 (Remarks to the Author):

Holly and colleagues have developed new genome assembly, pan-genome graph construction, read mapping and variant detection methods and applied these to both existing human resequencing data (biobank) and a new set of resequencing and ONT data from Icelandic-origin samples. The novel methods appear to offer some improvements over existing software (but see below), the new reference genomes are likely of reasonably high quality (but see below), and the discovery of previously undetected variants with potential clinical applications is interesting from a biological and potentially computational perspective.

Overall, I very much enjoyed the biological aspects of this paper, but I have one general gripe: throughout I had trouble detangling the lines of logic supporting method benchmarking and novel biological discovery. I think simply splitting out the analyses so that method validation (benchmarking) is always done for all new methods and existing methods with *exactly* the same input data, then biological analysis and interpretation is done using only the optimal method.

Below, I outline a number of comments that I hope will help improve future drafts.

- John T. Lovell (11-Apr 2025)

> Thank you for your summary and interest in the paper.
>
> We agree that benchmarking should be done on all the methods with the same input. We followed that principle as much as possible in our method benchmarks of simulated data, HG002 data and the selected 19 Icelandic samples data.
>
> In our analysis of the Icelandic cohort and related discoveries of variants associating to disease, we only ran Weaver and compared the results with our previous cohort based on linear reference mapping. Running all existing pangenome mapping methods on such a large scale would be very expensive and time-consuming. We understand these comparisons are intertwined with the method benchmarks, but we think they are valuable to put our work into context. In the benchmarks, they show that even if pangenome methods demonstrate incremental improvements compared to linear reference methods, these small improvements accumulate and result in large gains at a cohort level, as shown in the manuscript.

Major comments

#####################
Genome assembly quality:
The choice of low accuracy ONT reads appears to be the major motivation for developing a novel assembly tool (see below). I was surprised to see that a genome wide base quality of Q60 in these assemblies. I would like to see more evidence that this per base quality is actually achieved (before not just being surprised, but also impressed), especially highlighting both

regions that can be polished with short reads and those that are too repetitive for accurate illumina reads to uniquely map. This analysis should be relatively straightforward if there is ONT data sitting around for one of the HPRC genotypes, or just simulating 7% error rate ONT reads from the outbred HG002, re-assembling with Emblask, and counting the number of mismatches (see comment above about benchmarking). I ask for this because I can envision a situation where the reported higher numbers of variants detected in the pangenome (especially short INDELs) is due to unresolved ONT sequencing errors, and better unique mapping of short reads could be due to random variance in repetitive regions across the pangenome that reduces the number of exactly duplicated mappings.

Thank you for the suggestion. It should be noted that the quality value achieved by the Emblask pipeline is less than Q60 in our benchmarks, it is instead closer to Q50. This is shown in Figure 1a and described in more details in Supplementary Methods 6. We have added to the Supplementary Methods the evaluation of Emblask on the HG002 dataset (Supplementary Methods 7). For this assembly, we used long and short reads from public datasets that have similar characteristics as the reads used for the ICE-ONT/ILMN assemblies. As a result, the produced HG002 haplotype assemblies have a completeness greater than 99% and Q49.35. All QC metrics for the HG002 haplotype assemblies computed by Emblask are on par with the ICE-ONT/ILMN assemblies.

#####################
Weaver and read mapping and variant detection improvements:
Throughout, the authors claim that the new Weaver software better identifies variants than existing software. I am very amenable to this idea, but I am having trouble finding compelling evidence of this reported result. For example:
(1) lines 63-65 highlights improvements from linear to reference detection (and not comparison to variant detection from existing graph-based software);

It is correct that the called variants in our Icelandic cohort are only compared to our previous variant callset, computed from linear reference-mapped reads, and not to other graph-based methods. In that analysis, we do not show whether Weaver is better at identifying variants compared to other existing pangenome mapping methods. Yet, we think highlighting those improvements is valuable for those considering switching from a linear genome to a pangenome reference at a large scale. This is largely unexplored by existing methods.

Furthermore, we have also benchmarked the existing graph-based methods on a smaller scale. Weaver w/HPRC-ICE was the fastest and had the most accurate results for small variant calling on the safe variants of the assemblies. These results suggested it would be (a) much more time consuming to run the other methods and (b) unlikely to yield better results based on their small variant calling accuracy. Based on these benchmarks, we decided to only apply Weaver w/HPRC-ICE on the Icelandic cohort

(2) other analyses show comparisons where methods and datasets are not reciprocally investigated so it is impossible to tell whether the improvements are due to using new resequencing data (lines 115-117; 207-219), different pangenome graphs (lines 144-151; and

see next section of comments), different sets of individuals (220-229: even though there are fewer individuals in the Weaver set, n samples need not have bearing on the number of variants expected), genotyping error rate via comparison of monozygotic twins, which would be a great benchmark, is only shown for one method (195-197);

> We agree it was not always clear what contributed to the improvements highlighted in the manuscript. Maintaining complete consistency and uniformity across all such parameters and data throughout the analysis is challenging, i.e. selecting one pangenome graph would necessarily mean excluding the methods that cannot make use its format or running them in a suboptimal manner. However, we added the following two analyses to reciprocally investigate methods and datasets:
>
> a) In the mapping method benchmarks, including Giraffe w/HPRC graph and Weaver w/HPRC-ICE graph, we reran the benchmarks using Weaver w/HPRC graph. As those results share the same pangenome graph as the Giraffe results, the only difference is the mapping method. Neither BWA-MEM nor DRAGEN support any of the HPRC pangenome graph formats and we cannot run Weaver on the DRAGEN graph because it is proprietary. These results are discussed in Supplementary Methods 13.
>
> b) In the full Icelandic cohort, when comparing the callset resulting from Weaver mappings to the previous callset using BWA-MEM mappings, we have added a comparison of monozygotic twin pairs (n = 88) as suggested. We made sure for this comparison that the same individuals with the exact same read sequence data were used (no new resequencing). Among variants where at least one of the individuals was a carrier, the Weaver set contained 4.9% more reliable variants. Similarly to the full set, we observed a sizeable increase in the number of variants after applying pangenome mapping.
>
> We hope that these new experiments and analysis better show that the improvements we observed are due to different methods and are not due to differences in input data.

(3) lines 142-143 and figure 4a-b are interpreted as showing significant improvement of Weaver relative to comparable software; however, variance associated with the very similar profiles does not indicate to me that there is much of a difference and the sample size n = 1 needs to be increased to at least 2;

> We simulated reads from the two haplotypes currently available in the T2T-Q100 project with of 1x sequence coverage (0.5x per haplotype) or 20 million reads 151 bp short-reads. Higher confidence in the results could be obtained with more simulated read coverage, rather than increasing the number of haplotypes, so we would rather think of the n as 20 million reads (or 1x). We think this is sufficient and larger than prior analysis, for example it is 10-fold more read coverage than in the published simulated mapping accuracy experiment in Sirén, J. *et al.* 2021. DOI:10.1126/science.abg8871.

(4) lines 160-174 purportedly report improved mapping with Weaver-DeepVariant; however, this approach only is the most accurate in low-quality regions which may or may not represent an overall improvement in variant detection.

We also reported evaluations showing the Weaver-DeepVariant pipeline performed slightly better on average than the others when evaluated across the entire genome. We highlight the improvements in low confidence regions since those regions have been less explored in previous genomic analysis studies. The overall improvements in variant calling accuracy are minor compared to the current state-of-the-art methods, however, our mapping method has clear advantages in other aspects, for example in compute time and it being open source.

(5) some comparisons (e.g. 243-245) test Weaver against linear single-reference mapping. In these cases, it’s impossible to tell whether the authors are providing evidence of the value of a graph per se or the graph construction/mapping method.

We do not see a practical way to evaluate the graph and the mapping method independently of each other. The graph would not be of any value for mapping without a graph mapping method, and vice versa.

#####################
Comparisons to existing graph-based methods:
The new approaches described in this manuscript may offer significant gains over existing graph-based methods, especially with respect to identifying rare variants in genomic regions that are difficult to characterize. However, the way existing graph methods were applied does not necessarily reflect current best-practices for those workflows (i.e., lines 110-112). Specifically, when comparing Weaver against Giraffe and DRAGEN, an allele frequency-filtered version of the HPRC graph was used. A more appropriate comparison might be to use a personalized pangenome approach to subsample graph haplotypes, construct a sample-specific subgraph, and then align reads to haplotypes that could already contain rare variants (Sirén et al. 2024, doi:10.1038/s41592-024-02407-2).

Thank you for the suggestion. We have added results to the manuscript for the VG-Giraffe personalized pangenome reference, shortened as GiraffePPR. Our results indicate that in most cases, this pipeline offers some slight improvements over the allele frequency (AF) filtered workflow we previously used. We did not remove the previous results with the AF-filtered pipeline as our prior communications with contributors of HPRC and VG indicated that the recommended Giraffe workflow is the one using that AF-filtered workflow. Instead, the main text only shows results for the better performing Giraffe pipeline. This also keeps the plots less cluttered. The main figure plots showing all methods are in Supplementary Methods 13.

As for DRAGEN, we believe we are using a workflow that reflects the best practices usage. We are using the proprietary graph distributed with the DRAGEN software, not an HPRC graph.

#####################
Comparison to “personalized pan-genomes”
Ideally, Weaver would have been compared against an application of this personalized approach to a graph comprising the HPRC genomes plus the ICE haplotypes. Attempting to construct a minigraph/Minigraph-Cactus graph for even a single chromosome with all haplotypes would be a heavy lift, computationally, and potentially an additional clear and convincing argument for Weaver over existing methods that require full graph construction prior

to subsampling. If such a test build is computationally prohibitive, the rationale for not applying existing personalized pangenome methods could at least be clearly addressed in the manuscript, especially given the emphasis on detection of rare variants that would be specifically screened by the existing graph allele frequency-filtering approaches referenced in the manuscript.

I do think the idea of partitioning SVs in an rGFA and small variants in a VCF is clever and potentially a great solution to the problem of haplotype collection outpacing the computational capability to compile all available haplotypes into a single data structure prior to relevant subsampling.

> As discussed in a previous comment, we have added a comparison to the personalized pangenome reference approach used with Giraffe. Thank you for the suggestion. We have not been able to make a pangenome with the Minigraph-Cactus pipeline using all HPRC-ICE assemblies due to the very high memory and time requirements.

#####################
Minor comments

It is not clear how small variants nested within structural variants are handled in the Weaver workflow. It seems as though these variants are not considered, given lines 128-129 (“...pangenome construction can scale to hundreds of human haplotypes if restricted to non-nested small variants.”), but this should be reiterated somewhere in the Methods.

> Weaver handles small variants nested within SVs. These variants would be positioned on supplementary contigs (not part of the baseline assembly) in the input VCF. This is equivalent of having small variants in the blue or green contigs of Figure 3a. The quoted sentence refers to the differences of pangenome construction algorithms in which discovering nested variation requires expensive all-to-all alignments of assembly sequences. By restricting to non-nested small variants, we can scale up to the hundreds of human assemblies in HPRC-ICE. Yet, we think including small variants within SVs would likely offer little value to this work. The pangenome graph only includes very common SVs (MAF>10%) and all the analysis is based on the primary contigs of the baseline assembly. The Results section of the manuscript provides a brief overview of the reliable variants called in supplementary contigs (lines 230-233).

I had trouble understanding why graphTyper and not the weaver-DeepVariant (which was the benchmarked method) was used to call variants in the biobank data (line 181).

> GraphTyper requires substantially less resources (~0.9 million CPU hours) compared to DeepVariant (>10 million CPU hours). We have added a note regarding the DeepVariant CPU estimates in the revised manuscript to elaborate on our reasoning for using GraphTyper. Additionally, GraphTyper produces joint-genotypes whereas DeepVariant only produces single-sample genotypes. Merging the DeepVariant genotypes would require more compute resources and to our knowledge, the only merging tool for this purpose is GLnexus, a software that has not been updated in 4 years and that has not been tested in a GWAS setting. Finally, a minor benefit of using GraphTyper is that its

results are directly comparable to our previous variant callset that was also generated with GraphTyper, reducing the bias due to using different variant calling tools.

There is not much reference to how the Y chromosome was handled in the assemblies or variant detection. Assuming it was fully assembled and included, but this needs to be more precisely described since it represents a challenge to all bioinformatic aspects of this project.

It is assembled and included in the Icelandic pangenome. We have not yet assessed how it can impact the analysis of the Y chromosome but it is certainly a research direction that we might explore in the future.

How were the small variant calls for the ICE haplotypes integrated into the Weaver reference set? Were the coordinates for those calls retained in the specific coordinate systems of their respective dual assemblies and annotated with respect to those rGFA nodes in a VCF or were they somehow converted to the GRCh38 reference coordinate system prior to indexing?

The process of how HPRC-ICE was constructed is described in Supplementary Methods 8. Briefly, we use a modified version of dipcall to outputs variant calls for each of the assembled ICE haplotypes. Variants were then filtered, normalized and merged. Dipcall represents the variant calls with respect to the baseline assembly (GRCh38 in our work). We have made updates to this description.

I think it could be made more clear throughout that all analyses in this paper are based on short variants. I think when folks read “pangenome,” they think “all variants.” Which are definitely present in the haplotypes they contribute, but that’s not what this paper is about.

Thank you for the suggestion. We have made more explicit in the description of our variant analysis that we are only investigating small variants.

Thank you for reviewing the manuscript and providing helpful feedback on it.

Referee #2 (Remarks to the Author):

I co-reviewed this manuscript with one of the reviewers who provided the listed reports.

Thank you for reviewing the manuscript.

Referee #3 (Remarks to the Author):

Review: Icelandic Pangenome

This paper presents the "Icelandic Pangenome", an extension of the HPRC pangenome to include several hundred haplotypes from the Icelandic population ascertained from long-read data. The paper uses this pangenome to ascertain small variants using short reads from tens of thousands of Icelanders. The authors demonstrate an increase in the yield of variants relative to using a standard linear reference and investigate two medically relevant variants that were only ascertained using the pangenome approach. The paper contains a very extensive amount of new methods work, principally in developing the Emblask de novo assembly pipeline for ONT sequencing as well as (and IMO more significantly), in developing a new short-read to pangenome mapping algorithm, Weaver. The paper clearly represents a large body of work by a team of field leaders and is impressive in its scope, it is also exciting that they demonstrate some concrete improvements in variant ascertainment leading to biomedically interesting results.

> Thank you for your feedback. We are glad our work resonated with you.

My major concerns/criticisms are as follows:

Emblask pipeline

(1) The Emblask pipeline/assembler is well described and contains some novel innovations. However, for a journal like Nature I question the value of spending a substantial amount of time describing this, given that:

> We believe that a pipeline like Emblask is necessary for this study and agree with the reviewer that such work is highly temporal due to the rapid advance in sequencing methods. We therefore acknowledge that future studies are likely to use more recent sequencing methods, still much of the processing may be similar. We note that the description of Emblask is less than 10% of the results section (288 words out of 3033).

(1A) It is really aimed at a previous generation of Oxford Nanopore Sequencing (R9.4), which is largely historical at this point because it exhibits a much higher error rate than contemporary ONT R10 which supersedes it in all ways and which has been widely available for more than a year. It is not as if the cohort of samples sequenced with R9 is that large - there are other studies already available that show larger such cohorts.

> We understand your concern regarding the ONT R9 and Emblask. We think our coverage of the Emblask algorithm in the main manuscript is adequate considering that our Icelandic samples would have had to be assembled from ONT R9 reads only without Emblask, which according to our findings would result in a large decrease of quality, i.e. from ~Q50 to ~Q31-39. The feasibility of the Icelandic pangenome reference with inferior assembly quality is unclear and consequently, it is uncertain whether our findings in the *GBA1* and the *CBS* genes would have been possible since the discovered variants were only found in the Icelandic haplotypes of the HPRC-ICE. While we acknowledge that ONT R9.4 has been recently phased out, large cohorts based on ONT R9 are still being published (e.g. Structural variation in 1,019 diverse humans based on long-read

sequencing. Schloissnig et al., Nature, July 2025, DOI: doi.org/10.1038/s41586-025-09290-7) as the time required to fully process and analyze large cohort might span several years. Furthermore, the HPRC-ICE cohort is only an instrument for Weaver to map and subsequently analyze the much larger Icelandic cohort of 57,630 individuals in this study. As large cohorts of ONT R10 or even PacBio HiFi long reads are still limited, we are confident that the release of Emblask is timely. In addition, Emblask can be adapted to ONT R10 with moderate changes to the codebase. The core phases of the pipeline would remain the same but each step could be adapted to a lower input error rate. Among the main changes is whether indels can be integrated in the phasing and whether the PMDV pipeline can be swapped for only DeepVariant which now integrates an ONT R10 model.

(1B) While the Emblask method is interesting and well done, there has been a lot of innovation in assembly algorithms and the only real comparison is to a Shasta/HapDup assembly, a comparison which is buried in the supplementary methods and shows a good improvement in the indel accuracy but not much more. If this is to be published in a high-profile journal I would suggest shrinking the text dedicated to it and definitely including a comparison to the latest ONT error correction methods - I have personally seen quite good assemblies for R9 from Hifiasm with its latest built-in error correction, as well when using Herro plus either Hifiasm, Verkko or the latest "Mode 3" from Shasta.

Thank you for your suggestions. We agree that there has been a lot of innovations in assembly algorithms over the past few years and it is our belief that assembling simultaneously short and long reads into a dual assembly is one such innovation. Hybrid assembly has been underexplored but our results show tangible improvements over R9-only assemblies for human genomes. It is difficult to compare assembly methods that have different input data requirements but we agree that such comparisons are necessary to establish a baseline and demonstrate performance. We have added a comparison of Emblask to the Shasta/HapDup and HERRO/hifiasm-trio pipeline in Supplementary Methods 7 using the same ONT R9 long reads for the HG002 sample in input. We show that the dual assembly generated by Emblask outperforms the ones produced by the other pipelines on several key metrics such as quality, completeness and variant call accuracy.

(1C) Finally, overall, the Emblask pipeline has a distinctly "not invented here" feel. The authors, for example, invent their own error correction methods and wholly omit discussion of the latest assembly innovations (which are pertinent even for these R9.4 reads) from other groups.

We would like to firstly note that many assembly pipelines use tools developed by others, as assembly requires a number of sub-algorithms which need not all be reimplemented. The main novelty of Emblask resides in its short/long reads hybrid assembly method which improves the assembly quality with respect to assembling from ONT R9 alone. We reviewed in Supplementary Methods 3 other existing hybrid assembly tools and their limitations. It is correct that Emblask is not entirely a new algorithm. Yet, as we couldn’t find any assembler producing ~Q50 dual assemblies from

ONT R9 and/or short reads for human genomes, we also did not see the benefit of implementing from scratch yet another assembler which reinvents algorithms that are already available. Instead, the Emblask pipeline only implements new algorithms or algorithms that are not readily available and makes use of the best state-of-the-art error correction, assembly and variant calling tools for the data available for the Icelandic population. The global and local error correction sections of the Emblask pipeline rely on our experience designing the Ratatosk algorithm that our group has published 4 years ago and that we are using within Emblask. The assembly and the polishing are performed by Flye as it is the most versatile assembler to work with corrected long reads. All the variant calling and phasing is done with PMDV with and without the model that we have trained for corrected ONT. We do not take credit for these algorithms which are explicitly cited in the relevant parts of our manuscript and Supplementary Methods. While there certainly exists other tools that could perform these individual tasks (e.g Verkko or Shasta for the assembly), each tool used in Emblask was carefully selected for its performance, its ability to work with our data as well as its modularity enabling it to fit in a larger pipeline.

Major Technical Nits

(2) (This also relates to the value of the Emblask pipeline here - so also consider in the point above) Despite putting a huge amount of effort into assembling the genomes, the authors don't actually appear to use the structural variations that are inferred (Supplementary Figure 12). Rather they go through a process of mapping the assemblies back to a reference and then inferring small variants from them. I think it is natural to ask if it would have been more straightforward to simply use the ONT data with PMDV/Clair to directly call small variants for the ONT data. I presume the authors did intend to use the SVs from the assemblies but at some point gave up. Regardless, this seems like a major missed opportunity that leaves you scratching your head and needs some admission/discussion. I would minimally like to see evidence that the small variants discovered using Emblask are better than using the more direct approach of reference-based variant calling (PMDV will do phasing). If there are benefits to the Emblask pipeline for this use case over that approach I think the authors should avoid obfuscation and make it easier to understand (I read the supplementary methods in full and am still not sure!).

We agree there is a lot of untapped potential with the SVs from the Icelandic assemblies, which we plan on exploring in our next iteration of the HPRC-ICE. We took the decision not to include Icelandic SVs in the first version of the HPRC-ICE as it would have drastically increased the complexity of the project and its duration. Pipelines for small variants and SVs typically use very different algorithms. In addition, many published studies on pangenome references have already focused on exploiting SVs while small variants have been mostly overlooked.

We have added in Supplementary Methods 7 a comparison of the phased variant calls from the Emblask dual assembly and from ONT reads aligned directly to GRCh38 for the HG002 sample. The same ONT R9 reads were used in both cases and variants were filtered using the same parameters when possible. More details on methodology are provided in the Supplementary Methods. We demonstrate in this comparison that the

Emblask callset yielded more variants (1.34% to 9.76%) than when called directly from the aligned ONT reads. SNP calling performance was similar between the callsets in both benchmarks but indel calls in the Emblask set outperformed the aligned ONT by more than 30% in both cases. We also show that while the callset from the aligned ONT reads has thousands of phase sets, the Emblask callset naturally has one phase set per chromosome. As a result, the switch NGC50 of the Emblask callset is more than twice as long as the one from the aligned ONT.

(3) The text describes using rGFA for the pangenome, and the supplementary methods give an overview of the model. rGFA is exciting in that it provides a way to define non-reference coordinates. However, as far as I can tell, the additional variation added to the graph from the ICE samples does not encompass these non-reference sequences (variants are only added on the reference). Further, from what I can tell, there is no attempt to use Graphtyper to call variants against these additional non-reference sequences. This feels like a major missed opportunity to demonstrate calling variants within non-reference, commonly polymorphic sequence. There is a discussion about non-primary GRCh38 contigs, but I think this is talking about contigs in GRCh38 that are not on the primary reference. I think the authors should either remove the rGFA discussion and make it clearer about the limitations of the current approach (being limited to small variants on the primary reference - as is true of other pipelines too (to be clear)), or attempt to call and quantify small variants in this expanded coordinate system (which would make things a lot more significant in my view).

It is correct that non-reference sequences were only added from the HPRC assemblies and not the Icelandic assemblies. The two main reasons for this decision are that the HPRC assemblies are more complete and of higher quality than the Icelandic assemblies but also that as only common non-reference sequences (AF>=10%) are considered, we do not expect many such common sequences to be absent in the HPRC assemblies. However, we have genotyped the non-reference sequences with GraphTyper. We found 483,408 reliable variants in 858 such contigs in Iceland. We think these results show great potential but as the downstream analysis pipeline is linear reference-based, we have not been able to phase, impute or associate those variant calls yet.

(4) The paper discusses how other pangenome pipelines filter variants and that Weaver does not. The paper says: "Because rare variants may have a large impact on the regulation of gene expression with significant consequences in clinical outcomes and disease studies, we show that including them in our mapping model enabled the discovery of pathogenic variants associating with disease in Iceland and in the UK." However, it is not clear to me that the results shown are actually due to this inclusion. Further, it is surprising that mapping is not adversely affected by rare variants - as the authors no doubt know, mostly rare variants have been excluded precisely because they do impact mapping accuracy. Some analysis and demonstration of these claims would really help.

We have reworded the sentence such that instead of “showing”, we are rather “demonstrating” that including rare variants enabled us to discover pathogenic variants. We are referring in this context to the analysis of two rare variants (<1% minor allele frequence) in *GBA1* and *CBS* that would have been excluded with an allele frequency filter. If they were excluded, our pipeline calls them at a much lower frequency than what we observed in the assembled sequences. Thus, we would argue that those two examples demonstrate well that rare variants should not be excluded from the pangenome.

We know of the adverse effects of including rare variants in mapping. We think this is largely because when other mapping methods are selecting the most likely mapping for a read, they make no distinction to whether the mapping is with a sequence observed in one assembly or in all assemblies. We address the issue by using a weighted alignment score in the mapping model. Personalized pangenome references are another way to address this issue, as the sequence present in all assemblies will necessarily be a part of the personalized subgraph while the unique/rare sequence is not a part of it for most individuals. We are looking forward to exploring different solutions to these issues in the future.

(5) The authors do a pretty reasonable job of benchmarking their Weaver/Graphtyper pipeline against single samples (It is interesting that in the only benchmark that is widely compared against (the HG002 T2T one) the Dragen pipeline appears better and the vg/DeepVariant pipeline is about the same.) However, what the authors do not really convincingly do is show that the increase in "reliable variants" ascertained by calling against the Icelandic populations is not a statistical artifact of differences between the Weaver/Graphtyper pipeline and the BWA-MEM/Graphtype based pipeline. The 6% of additional "reliable" variants might be due to differences in parameterization, and indeed might be an overestimate of the true difference because these additional variants are not validated in a reasonable way. The authors could show the calling of these additional variants using their held-out pacbio data, but do not. So, while I think the reported result is promising, it really needs more careful validation before I'm ready to accept it - and certainly more controls are needed for publication here.

Thank you. We first would like to point out that one form of validation that was performed is the imputation of the variants into a larger reference panel using long-range phasing. After imputing the Weaver/GraphTyper variants, we observe a large increase of well-imputing variants compared to the imputed BWA-MEM/GraphTyper variants set (nearly 7 million more well-imputing variants). In addition, we have added further validation of the Weaver/Graphtyper and BWA-MEM/Graphtyper comparison as other reviewers also pointed out. To address this concern, we have evaluated the same set of monozygotic twins in the two datasets. Finally, we note that in single sample benchmarks, the DRAGEN pipeline is only slightly better when restricting to the high confidence regions. In those regions, all methods perform extremely well and very similarly. An argument could be made that using a pangenome reference for those regions is not as relevant as for other more complex regions. The motivation of our method was to capture more of the human genome, beyond the high confidence regions, and we do see a larger

difference among those regions where Weaver/DV pipeline performed better than DRAGEN.

(6) The authors compare Weaver-Graphtyper to BWA-MEM/Graphtyper, but it would also be useful to know if other, existing pipelines would call the GBA1 variants. It says that Dragen does not. Does vg/DV? Does BWA-MEM/DV? Again, this is an important claim and it should be carefully interrogated.

In this analysis, we only assessed the different pipelines that have been applied on the 500k UKB dataset as this is a very time-consuming process. The problem with the previous genotypes of BWA-MEM and DRAGEN was that the *GBA1* variant had an underestimated frequency.

Minor points:

There are some missing (??) figure references in the supplementary material. Looks like a latex issue.

Thank you for pointing out those missing references. We have now fixed them.

The authors discuss pangenome personalization in their discussion as a future direction. However, they don't attempt to use it when comparing to vg - it would be good to update this. That pipeline has been available and published for around a year.

We have updated our comparison to VG Giraffe using the personalized pangenome reference (see details in the response to reviewer #1).

I struggled with this description: "As a minimizer might span multiple segments, Weaver requires k to be an odd number in order to index each minimizer using the position of the base at its center. This guarantees that minimizer occurrences are always indexed with respect to a single segment of the graph. It also prevents to query the graph for minimizers in both directions as the orientation of the read sequence compared to the sequences in the graph is unknown." Are you just saying you avoid palindromes by using an odd-length minimizer?

Thank you for pointing out that poorly worded description, which we have reworded in the revised manuscript. We use odd k not to avoid palindromes but rather to guarantee that k-mers have an unambiguous center base position.

The version of some tools used is pretty old and some results might be a little out of date as a result.

We have updated the benchmarks against Giraffe to the more recent personalized pangenome reference pipeline. Some other tools we used have more recent minor

versions released but please note that running experiments and analysis for large datasets necessarily takes a long time. We do not think rerunning our experiments with those newer versions will have an impact on the interpretation of the results.

Referee #4 (Remarks to the Author):

Holley, Eggertsson et al present a manuscript that describes two software tools and methods to enable pangenome graphs construction and mapping at biobank scale: Emblask and Weaver. They apply the methods to data from several datasets from Iceland and the UKBiobank. As a proof of concept, they find SNPs in genes known to cause severe disorders.

The advances are mainly technical. I don't think the results are of particularly broad interest. I found the manuscript to be descriptive, but missing a discussion of many key questions, which I ask about below. I am concerned about the apparent bias in genotyping visible in the allele frequency distribution in Figure 5. The results about discovering SNPs in known pathogenic genes are a good proof of concept of the approach, but I wonder how much more discovery there is to be had besides them.

I think that these methods will be useful for people who are building pangenome graphs and mapping to them, including those working in species besides human. I think that allowing VCF files to be used will enhance usability of pangenome graphs. The software appears to run, although I did not find a detailed walk-through/tutorial.

> Thank for your feedback. We have added a README showing how to run Weaver.

As far as I can tell the manuscript appropriately references prior work. The methods section was appropriately detailed. Some of the treatment of uncertainty was inadequate (see specific comment below).

Major concerns
- Figure 5: allele frequency spectrum for SNPs has a massive uptick at 50% -- why? I believe this is an artifact of something in the author's pipeline.

> This uptick is indeed most likely caused by artifacts but it only corresponds to approximately 3,000 variants out of nearly 100 million. More specifically, these SNPs are genotyped with excess amount of heterozygous genotype calls which can occur in regions with common segmental duplications. Each SNP allele is then only the difference between the duplicated sequence and the reference sequence. This problem is ubiquitous in variant calling pipelines. To address this, we applied a statistical Fisher's exact test to filter out variants with excess number of heterozygous calls. See more details in Supplementary Methods 4. However, the data shown in Figure 5 has not been filtered using this test to be consistent with the previous linear reference callset we are comparing to, which did not have this filtering criteria.

- Figure 5: could the authors explain the periodic behavior of the indels?

This periodic pattern is likely caused by tandem repeat mutations in the human genome. In particular, short tandem repeats (STRs) are highly prevalent and have a high mutation rate with motif sizes of 2-6 bp. Indel sizes dividable by those lengths are thus observed more often. For example, a 12bp indel is dividable by 2, 3, 4 and 6bp, so it is far more frequently observed than 11bp indels.

- Line 216: why is this comparison across a different set of samples? I am interested in comparing the number of variants called across the same set of samples. If the samples differ, it's harder to interpret the difference.

This is a valid concern and was also brought up by another reviewer which suggested to evaluate the same monozygotic twins in the two callsets. We have added this comparison and see a clear increase of the number of twin consistent variants after applying Weaver with the HPRC-ICE (Figure 5f).

- Line 231: here again, the sample sizes differ, making it hard to compare the number of variants called.

We agree that these numbers can be challenging to compare as the set of samples is not the same. We have addressed this by performing a new analysis based on the monozygotic twin pairs present in both callsets. In that analysis, we compared the results based on the exact same read data. We also see an increase in the number of variants in the Weaver mapped data.

- Line 219: what is the comparison at dark regions, as previously mentioned?

We cannot make a comparison as we did before because the other results only contain GRCh38 and do not have these supplementary contigs.

- Line 168: Some of these differences are quite small. Are the differences in F1 score significant across the methods?

Those differences are probably not considered significant between the mappers. However, safe variants are more likely to be located within easily mappable regions where the mapping to a pangenome has limited benefits. A similar observation can be made when comparing against the high confidence regions of the GIAB truthset. Small differences in high confidence regions have also been reported in other studies such as the PrecisionFDA truth challenge v2 (Olson, N. *et al.* 2022. DOI: doi.org/10.1016/j.xgen.2022.100129).

However, we see larger differences when restricting the benchmark to low confidence regions (Figure 4f). The genome-wide improvements accumulate and are more meaningful when scaling up to a larger variant callset, as we demonstrated in our Icelandic population analysis.

We also note that our method has more advantages in other areas compared to the other methods, for example in compute time and it being open source.

Minor concerns
- Line 79: the text would benefit from clarifying what polishing is.

We have described in more details what polishing is in the introductory description of the Emblask pipeline. More algorithmic and methodological details about polishing are provided in Section 3.5 of the Supplementary Methods, including an illustration of the different polishing steps in Figure 8 of the Supplementary Methods.

- Line 107: Does the rate of increases differ among variants?

There is a slightly increased rate of indels, as observed by the analysis of singletons. This might be explained by indels being more likely to have a detrimental functional impact as well as indels have higher error rate in the input read data.

- Line 109: Are singleton variants in SNPs and indels found in different parts of the genome?

Yes. We do not see any unusual or interesting pattern in the distribution of the singleton variants across the genome.

- Line 119-120: Do these results refer to a specific display item?

No, we are just pointing out that we designed our mapping model to have no allele frequency filter because we think rare variants should not be excluded during mapping.

- Line 147: how is mappability measured here?

Mappability is defined by the GRCh38 stratifications regions from the GIAB consortium. In short, low-mappable regions are mainly composed of substrings that are not unique across the reference genome and therefore represent regions that are challenging for short read mapping. The publication for those regions is referenced for further details.

- Line 162: what is the allele frequency distribution of the safe variants? are common alleles more likely to be safe?

The safe variant set is sample-based and are not annotated with allele frequencies. Our filters described in Supplementary Methods 8 should not favor common alleles. One exception though is that we require the genotype calls to pass a Mendelian consistency check, so true *de novo* calls would not be considered safe but as those are usually very rare, they would not have much impact on our benchmark results.

- Figure 2 legend: A description of how completeness and quality are measured in the legend would be helpful.

Thank you for the suggestion. We have added a description for completeness and quality values in the caption text.

- Figure 2d: Can you use this curve to estimate when the number of small variants will saturate?

We do not think we can estimate such a saturation point from just 788 haplotypes, especially considering that different populations having their own small variant saturation points are represented in the HPRC assemblies. However, our takeaway from Figure 2d is that there is relatively little to be gained from assembling more Icelandic haplotypes and we should rather focus on individuals from different ethnic backgrounds for the next iteration of the pangenome.

- Figure 4d: is it correct to say that the runtime complexity (i.e. O(n)) with respect to depth of all three methods is the same, but Weaver has a smaller constant?

> Yes. This was also our observation from this benchmark so we think this is a correct assessment.

Referee #4 (Remarks on code availability):

Code provided shows a sample build and run of Weaver. There is no README, but there is an bash script that builds and runs Weaver. I think having a detailed tutorial would be helpful for users.

> Thank you for reviewing the code and the suggestion. As we mentioned above, we have added a README to the Code Ocean container. Further details on how to run Weaver are on the main code repository at: https://github.com/DecodeGenetics/weaver

Referee #1 (Remarks to the Author):

The authors have provided some updates to their manuscript and detailed explanations in the response to reviewers. Overall, I think the manuscript is somewhat improved; however, all of the general concerns I raised in the previous review (and many of the concerns raised by other reviewers) remain.

Thank you for your comments on the original manuscript and its updated version. We acknowledge that some of the comments raised in the previous round were not fully addressed while some explanations provided in the responses were not added to the main manuscript. We have now addressed all these concerns in this revised manuscript.

Manuscript main text updates

In the revised manuscript, we have responded to your general concerns by splitting more explicitly the method benchmarks and biological analysis. The manuscript has been restructured accordingly. In the method benchmarking, we focus solely on comparing Weaver to existing methods with the same input data, as described in a new section of the main text titled “Weaver improves mapping accuracy and single sample variant calling performance” (lines 156-197).

The sections “Weaver scales to biobank-size analysis” (lines 198-277) and “Weaver mapping to the HPRC-ICE uncovers sequence variants associating with disease” (lines 278-326) discuss the biological analysis and interpretation. In these sections, we fix the mapping and genotyping framework to Weaver with HPRC-ICE based on the benchmarking results provided earlier in the manuscript. The biological analysis is still put into context with respect to existing results computed from a linear reference but we explicitly note in the main text that these are not based on the exact same input data (lines 199-201).

In many places, the authors provide sufficient responses to the issues raised by reviewers in the rebuttal document, but they do not address these issues in the text (not just my comments but this is true of the other reviewers’ as well). This is not sufficient - we are asking about these things because they need to be clarified in the manuscript. If you believe that it is not important enough to change in the main text, then that choice needs to be defended in the rebuttal.

We recognize that several clarifications were previously confined to the response letter or the Supplementary Material. These have now been incorporated in the manuscript where they are necessary for correct interpretation. We reviewed every concern from the previous review and ensured each one is now addressed in the manuscript. The changes made to the manuscript are listed below.

Manuscript main text updates

In the last revision, reviewers suggested two additional experiments to evaluate Emblask against other existing methods. We have added a summary of those results to the main text, further emphasizing the value of Emblask. We added the following into the main text (lines 88-93):

” We evaluated Emblask with HG002 data and compared it to leading ONT R9.4 assembly pipelines. Our results indicate our approach yields assemblies with superior assembly quality and completeness (Q49.35 and 99.01%). Compared to variants called directly from mapped ONT R9.4, our HG002 assemblies yielded 1.34% to 9.76% more variants, longer phased regions (6.157 Mbps switch NGC50) and more than 30% greater indel accuracy (Supplementary Methods 7)”

We have updated the main text regarding the separation of benchmarks and biological discoveries as discussed in the point above.

The main text has been updated to discuss the value of the new benchmarks performed with Weaver w/HPRC (lines 193-197). Details for the experiments are in Supplementary Methods 13.

For the simulated benchmark, we have added the clarification that reads were simulated from both haplotypes of the T2T-Q100 HG002 dual assembly (lines 159-160).

We have added a discussion about the recently discontinued ONT R9.4 chemistry (lines 359-362).

We have added clarifications to why the non-reference contigs were not analyzed further (lines 259-260).

For the large-scale analysis of the *GBA1* variants with methods other than Weaver, we had argued that the experiment would be too time-consuming. We had not considered restricting the experiment to a subset of the input. We have now run all methods on the two Icelanders who are carriers of the pathogenic *GBA1* variant in HPRC-ICE. Except for Weaver, all methods missed or filtered out the variant (lines 295-298).

We added an explanation for the periodic indel size pattern (lines 218-219).

Throughout, I still had trouble distinguishing between whether the results are about performance of the methods or new biological discoveries. As before, the ground truth methods are confusing as well. For example, Figure4 provides both methodological comparisons between equivalent methods (graph alignment method comparisons 4a-b) and non-apples-to-apples comparisons related to the approach in general (e.g. single reference issues 4c).

Thank you for noticing the issues in the analysis visualized in Figure 4c. This analysis is part of the methods benchmarks, so it should have been following the same principles as the other benchmarks (all methods tested, exact same input, etc). We have updated the analysis discussion in the main text (lines 166-168) and Figure 4c, such that it now includes an evaluation of all the methods. Unlike the previous Figure 4c, all non-primary contigs were excluded from the dark regions computation, resulting in a lower number of dark bases for Weaver and BWA-MEM compared to the first manuscript. Finally, we had incorrectly reported 210 CDS regions overlapping bases dark to BWA-MEM but not Weaver, while this number was in fact number of genes with at least one CDS overlapping the dark regions unique to BWA-MEM. We now report the correct number of CDS regions which are not dark anymore after Weaver mapping (360).

Manuscript main text updates

Lines 164-165 reports that non-primary contigs are excluded from the dark regions computation

Also, I don't find the reasoning that it is computationally challenging to to be a persuasive argument to not provide a comparison to a baseline within identical input data for each method (this is provided as a reason to not do several comparisons). New software requires such comparisons, and the improvements need to be stated clearly. This is especially important here, as the lead contribution of this work is a new assembler. Perhaps just use smaller datasets and compare those directly?

Thank you for pointing this out, we initially did not appreciate adequately the importance of evaluating the other mapping methods when it was infeasible to run them on the full dataset. We have now run the suggested experiments on a smaller dataset to address these concerns. For context, we had argued it would be too computationally challenging in response to: (1) Checking whether vg/DV and BWA-MEM/DV would be able to call the associating *GBA1* variant (which was done when we only applied Weaver to the 63K Icelandic and the 500K UK Biobank datasets) and (2) in an explanation to why we did not run all methods in our efforts to find disease associating variants in the 63K Icelandic and the 500K UK Biobank datasets. Subsampling the dataset means we greatly lose power to find disease associations. But by subsampling and running the suggested methods, we could show that these existing state-of-the-art mapping methods cannot capture the variant in *GBA1* among the known carriers, whereas Weaver/GraphTyper captured it with the exact same input data. We believe this new experiment has strengthened the manuscript, and so we are grateful for your suggestion.

Furthermore, we would like to clarify that Emblask is not a new assembler, but an assembly pipeline built around an existing assembler (Flye, see lines 80, 630, 638), hybrid error correction tool (Ratatosk, see line 77, 627) and variant caller (PEPPER-Margin-DeepVariant, see line 83, 620) among other tools. In our work, we also use the existing assembler hifiasm/hifiasm-trio for the PacBio data (see lines 105, 687-691). The contribution of Emblask lies in enabling hundreds of dual assemblies using ONT R9.4/Illumina and suitable for pangenome construction in terms of quality and completeness, which was not previously feasible in this cohort.

However, it is our opinion that Emblask is not the lead contribution of the paper, our contributions also include Weaver, the Icelandic pangenome reference and applying these at population-scale to discover novel disease associations. Among those, we think the Icelandic pangenome reference is the lead contribution, hence the manuscript title, but we can see that the Emblask and Weaver methods might be more of a focal point to some readers. We have now added further clarifications of our contributions in the Discussion (details below).

Manuscript main text updates

We have added the following to the main text based on the analysis of the *GBA1* variant in Iceland with BWA-MEM/DeepVariant, GiraffePPR/DeepVariant and DRAGEN:

”The variant was missed or failed quality filters in the short reads of the two Icelanders whose assemblies had the variant after mapping and genotyping with BWA-MEM/DeepVariant, GiraffePPR/DeepVariant and DRAGEN.”

We also added the following to the main text (Discussion) to make our contributions more explicit.

”Our contributions in the Emblask assembly pipeline, the Weaver mapper and the Icelandic pangenome reference HPRC-ICE are made available to the research community.”

Other less critical stuff that was not addressed sufficiently in the revised manuscript:

The improvements of the new methods are not obvious when compared to GiraffePPR (outside of computational time, e.g. 4d); the specific places where improvements are made should be clear. In particular, GiraffePPR could be expected to offer significant improvements in calling rare variants over the frequency-filtered approach.

Thank you, the text in our previous submission lacked clarity. Our benchmarks show that Weaver outperforms GiraffePPR in both precision and sensitivity while requiring substantially fewer computational resources. In addition, GiraffePPR could not be applied to the HPRC-ICE pangenome due to reference size constraints, which precludes its use in the central analysis of this manuscript.

The improvements of Weaver over GiraffePPR might not have been clear and apparent in the previous version of the manuscript. Outside of reducing mapping time by 40%, Weaver/DV also outperforms GiraffePPR/DV in variant calling performance based on the benchmarks against the HG002 T2T-Q100 truth set (Figure 4f, lines 190-192) and the variants yielded by the 19 Icelandic haplotypes (Extended Data Table 1, lines 178-181). For example, Weaver had on average 8,861 (11.5%) fewer false positives and 368 (0.2%) fewer false negatives than GiraffePPR in the 19 Icelanders. As a result, Weaver had higher sensitivity and precision than GiraffePPR. We think comparing those counts of FP/FN will make the improvements more explicit in the main text.

Resource requirements and variant calling performance are highly important aspects to consider when selecting a mapper. So even if GiraffePPR had been published when we started our biobank scale genotyping, our benchmark results suggest Weaver to be the preferable option due to its greater variant calling performance and much lower resource requirements.

Another reason to choose Weaver over Giraffe/GiraffePPR, which was not explicitly stated in previous version of the manuscript, is that we have not been able to use Giraffe with HPRC-ICE due to the high number of haplotypes in it. Since the manuscript is partly about the construction of HPRC-ICE, it would have been a missed opportunity to restrict the analysis to the smaller HPRC pangenome.

The second part of this comment concerns the comparison of the frequency-filtered approach (Giraffe) and the personalized pangenome reference (GiraffePPR). Both methods differ from our approach in Weaver (lines 123-127). Giraffe authors have made the claim that GiraffePPR is better than Giraffe at calling rare variants (i.e. in the abstract of Sirén J., 2024, DOI: doi.org/10.1038/s41592-024-02407-2). We think the authors of Giraffe have already made good arguments and analysis to support that claim. Our current benchmark results suggest GiraffePPR has overall higher variant calling performance than Giraffe, consistent with their published findings. Therefore, we think comparisons between those previous approaches have already been made adequately in previous work.

Manuscript main text updates

We have added to the main text two more explicit reasons for using Weaver over GiraffePPR.

“In our benchmark of 19 samples, Weaver called 11.5% fewer false positives and required 40% less time than GiraffePPR, the best performing previous method.”

We also added to the main text as a further rationale that we did not manage to run GiraffePPR with HPRC-ICE (lines 193-194).

At several points you state that a big improvement of this method is the handling of complex nested variants; but you also state in the response to reviews that “we think including small variants within SVs would likely offer little value to this work”; this is an important sentiment that was not conveyed in the manuscript. In the presented workflow, such variants are situated on non-reference contigs and functionally invisible to downstream analytical steps. Please add this text into the manuscript with some descriptions of why it is so; that way, the reader can contextualize your decisions.

Thank you for your suggestion. You are correct that due to limited support of downstream methods, these variants are functionally invisible to them. Each sample has highly variable ploidy which often cannot be inferred directly from the mapped sequences, resulting in downstream tools often assuming incorrectly 2 haplotypes (diploid). Another issue is that these contigs have no functional annotation available (excluding the CHM13 haplotype). A wide range of annotations is important to further understand variants and allow us to compare them to prior callsets.

Manuscript main text updates

Added the following in lines 259-260:

“Because of their variable ploidy, lack of available functional annotations and limited support in downstream methods, we excluded them from other analysis.

Referee #2 (Remarks to the Author):

I co-reviewed this manuscript with one of the reviewers who provided the listed reports.

> Thank you for your valuable feedback and your time in reviewing the manuscript.

Referee #3 (Remarks to the Author):

Thanks - the authors have provided reasonable responses to all my queries and quibbles, although much of it has not changed the overall shape of the manuscript. However, I still have concerns about the significance of the developed methods:

> Thank you. We think the experiments you suggested better show why Emblask was chosen over other assembly pipelines and calling directly from the long reads. We have ensured that these are now fully incorporated into the manuscript.

(a) the rather out of date nature of the Icelandic long-read sequencing data and knock-on value of the Emblask pipeline,

> We acknowledge that ONT R9.4 has been recently phased out for the more accurate R10 chemistry. The release of new sequencing technologies or the major update of existing ones is always welcome. However, ONT R10 was only introduced in 2023 and ONT R9.4 discontinued in 2024 which makes it challenging to strictly use new data from these updated versions on large-scale projects that span several years. An example of this is the analysis of SVs in 1,109 genomes, published recently, which also uses ONT R9.4 (Structural variation in 1,019 diverse humans based on long-read sequencing. Schloissnig et al., Nature, July 2025).
>
> Finally, Emblask assembles ONT R9.4 and Illumina together which enabled us to produce assemblies with mean Q50 and near 99% completeness. We think these assemblies are of sufficient quality to be included in any future pangenome set built from ONT R10 reads alone.
>
> **Main manuscript text updates**
>
> We have the following text to the Discussion section:
>
> “In addition, while ONT R9.4 is being discontinued for the more accurate R10 chemistry, our hybrid assemblies with Emblask reach Q50 quality and near 99% completeness, indicating that they can be included in any future pangenome built upon ONT R10 assemblies.”

(b) the relatively marginal improvements in variant calling results achieved as viewed through the lens of GRCh38 projection (it feels like diminishing returns because everything is ultimately forced into GRCh38 space),

We agree that lifting back our analysis to GRCh38 is suboptimal but it is also a necessary step to fit into existing pipelines and demonstrate the value of pangenomes over linear reference genomes. Pangenomes references are not in widespread use nowadays due to the lack of available annotations and software for pangenome analysis. Updating linear references from one build version to another has taken the community several years and we believe that fully updating annotations to pangenomes will take several more years.

We have added a rationale to the main text for our decision to project to GRCh38 linear coordinate system.

Main manuscript text updates

Lines 63-65 we added:

“The linear coordinate system built into the pangenome reference allows for direct access to available genome annotations and enables comparison with linear reference callsets.”

(c) the lack of value achieved by ignoring the SVs in the Icelandic assemblies (which again makes me really question the value of the Emblask pipeline as presented - I stand by my comment that they could have just used this data to do variant calling against 38 and added it the pangenome VCF),

Comparing our approach to directly using mapped long reads (with PMDV) for variant calling was a great suggestion that strengthened the manuscript. We had previously added to the Supplementary Methods a comparison of the variants called from the HG002 assembly made with Emblask to the ones called directly from ONT R9.4 reads. This benchmark showed that 1.34% to 9.76% more small variants could be extracted from the assemblies, that the phased regions were nearly twice as long (6.15 Mbps switch NGC50) and that the indel F1 was about 30% greater. These numbers are now reported in the main manuscript. It is our opinion that these three results alone demonstrate the value of the Emblask pipeline for this work. We agree there is more to be achieved using the SVs in the assemblies but in the exploratory phase of this project, we initially saw limited benefits of adding rare Icelandic SVs to the common SVs from the HPRC assemblies already present in HPRC-ICE. Given that the ICE assemblies were of lower quality and contiguity than the HPRC assemblies but also that including the rare Icelandic SVs would substantially increase the complexity of the graph, we took the decision not to include these SVs to avoid delaying the mapping and the analysis of our Icelandic cohort. We have added to the main text a justification to why the Icelandic SVs were not included. Moreover, we knew that the HPRC consortium was already well underway in assembling their next set of individuals composed of 5-fold more haplotypes compared to their first release so leaving the Icelandic SVs for the next iteration of HPRC-ICE, which would include include HPRC r2, made sense. Finally, considering that other published studies on pangenome references have already focused on exploiting SVs while insufficiently exploiting small variants, we decided to put our focus on the small variants.

Main manuscript text updates

We added on lines 88-93:

“We evaluated Emblask with HG002 data and compared it to leading ONT R9.4 assembly pipelines. Our results indicate our approach yields assemblies with superior assembly quality and completeness (Q49.35 and 99.01%). Compared to variants called directly from mapped ONT R9.4, our assemblies yielded 1.34% to 9.76% more variants, longer phased regions (6.157 Mbps switch NGC50) and more than 30% greater indel accuracy (Supplementary Methods 7).”

And on lines 111-113:

“Since adding SVs increase graph complexity and the HPRC assemblies were of higher quality and contiguity than the ICE assemblies, we chose not to add SVs from Icelandic individuals to the graph.”

and (d) the focus only on variants mappable to GRCh38.

We have added further clarification why we did not focus our analysis beyond GRCh38 primary contigs.

Manuscript main text updates

Added the following in lines 259-260:

“Because of their variable ploidy, lack of available functional annotations and limited support in downstream methods, we excluded them from other analysis.”

While I think the paper is technically sound and definitely very interesting from my perspective as a methods geek, it does feel of questionable relevance to a broader, less methods focused audience.

Thank you for your interest in the paper. We agree that the manuscript has a large focus on the methods, but we also think some non-methodical aspects might be interesting to some. In particular, our discovery of the disease associating uncovered variants with their disease associations will speak to a less methods-focused audience. While there exist other papers in the literature focusing on computational pangenomics methods, this paper is in our opinion the first to demonstrate that pangenome mapping at population-scale is not only possible but also delivers new meaningful results. Our findings demonstrate that reference bias is not a minor issue but a systematic impediment to comprehensive genetic analyses of diseases.

Manuscript main text updates

We added the last sentence of this response to the main text (lines 352-353).

Referee #4 (Remarks to the Author):

The authors have addressed my comments.

Referee #4 (Remarks on code availability):

The authors have addressed my comments.

Thank you for your valuable feedback and your time in reviewing the manuscript.

Referee #1 (Remarks to the Author):

The authors have done a nice job of addressing our previous gripes. We think the methodological aspects of the article are much improved — this will be a very nice contribution! Just one minor point: caption for Figure 4C should include how values were calculated (e.g., are these the total lengths of regions not covered by short reads from any of the 19 libraries?). Nothing else from us.

> Thank you for your comments and suggestions. As suggested, further details have been added to the caption of Figure 4C. Dark regions are computed by first aggregating together, for each reference base, the coverage, base qualities and number of aligned reads with low mapping qualities from the aligned short reads of the 19 samples, then averaging per base. A more detailed description of dark regions is provided in Supplementary Methods 8, subsection “ICE-ONT/ILMN evaluation”.

Referee #2 (Remarks to the Author):

I co-reviewed this manuscript with one of the reviewers who provided the listed reports.

> Thank you for your comments and suggestions.

Referee #3 (Remarks to the Author):

Thanks, I think the paper is stronger for the changes made. I also continue to appreciate the level of technical development and ambition, particularly with respect to the Weaver mapper, which looks like a useful, well architected advance. I really want to leave this review at that - really I do, however, I still think the paper, as it stands, continues to have major issues: (1) it is unfortunately selectively presenting somewhat marginal improvements, (2) it continues to focus on a now archaic (in terms of sequence technology) assembly pipeline that results in very small downstream improvements for genotyping, and (3) doesn’t actually make any of the data (even in derived, de-identified form) available.

> We thank the reviewer for his continued engagement and his contributions to the improvements of the main manuscript through the suggested additional analysis and comments.

1) In our view, the main contributions and results of the study are not marginal improvements. While improvements in high-confidence regions are necessarily modest (lines 183-186) due to near-saturation of existing methods, often >99% F1 (Olson *et al*, Cell Genomics, 2022, doi.org/10.1016/j.xgen.2022.100129), the impact at population-scale is substantial (lines 241-249). Specifically, mapping to the HPRC-ICE pangenome results in a 6.17% increase in reliably called variants and enables the discovery of disease-associated variants that were missed by previous linear reference-based approaches.

We understand that presenting only the low-confidence regions in Figure 4F (the HG002 benchmark) and Supplementary Figure 17 could be perceived as selective. This choice was motivated by the fact that most errors from short reads variant calling pipelines are concentrated in difficult genomic contexts such as low-mappability regions where pangenome reference mapping is expected to bring the most gains. In this revision, we have updated Figure 4F and Supplementary Figure 17 to include all regions (high- and low-confidence). Discussions about the stratified results are in the main text (lines 189-192) and in Extended Data Table 1. This presentation matches how the results for the 19 Icelandic samples are presented (Figure 4E). We appreciate the suggestion, which strengthened the clarity and readability of the manuscript.

2) We appreciate the reviewer's perspective and have reduced the presentation of Emblask by ~10% in the revised manuscript.

We describe the Emblask assembly pipeline because it was essential for building the Icelandic pangenome reference and uncovering the two highlighted disease-associated variants. We therefore retained the description of the main methodological steps necessary to understand the downstream analyses.

We have also noted the Emblask methodology is not specific to ONT R9 (lines 359-361). The R9 chemistry was discontinued in July 2024, less than eight months prior to submission, and datasets generated using this technology continue to support recent large-scale studies (e.g. Schloissnig *et al*, Nature, 2025, doi.org/10.1038/s41586-025-09290-7). It is expected that large-scale sequencing projects are not necessarily based on the most recent sequencing technology as such projects span multiple years, from data generation through analysis, interpretation and manuscript submission. Our work encompasses sequencing, assembly, pangenome construction, population-scale mapping of 57,630 individuals, variant calling, imputation, and downstream disease

association analyses. The sequencing technology used reflects what was available at the time of data generation.

3) The Icelandic pangenome reference (HPRC-ICE) will be made available, as stated in the main text in Data availability (lines 762-768) and Discussion (lines 345-347). We are providing instructions for reviewers to download HPRC-ICE.

In terms of the first issue, selective presentation, I would say:

- The authors are using very old versions of software to compare against, particularly the Dragon software they use (the version is only given in the supplement!) is from July 2023. I am aware that subsequent versions have substantially improved performance since that time. Similarly, I know versions of the DeepVariant for Giraffe have been steadily improving. It may be a chore, but it is not reasonable to compare to these very old software at this point, and I think things would look quite different compared to the latest Dragen.

We understand that our rationale for selecting software versions was not sufficiently explained in the manuscript. In the methods benchmarks, we selected the most recently published and validated versions of each method. We appreciate the reviewer's note regarding the release of newer software versions. We have now explicitly stated in the manuscript that we chose the latest published versions for our benchmarks (line 154).

Our experiments also use the most recent version of the HPRC graph (v1.1) for which the provided index files are compatible with Giraffe v1.51 (the version used in this work) according to the README. We verified that these index files are not compatible with the more recent versions 1.53 and 1.73 of Giraffe. We therefore determined that this version of Giraffe is recommended for HPRC graph mapping. Furthermore, Giraffe's development has heavily shifted towards long reads mapping in more recent versions. It is unlikely those will have meaningful impact on the results derived from short reads mapping.

While recent DeepVariant versions have introduced a new pangenome-aware model (Asri *et al*, 2025, doi.org/10.1101/2025.06.05.657102), both the software and the accompanying preprint were released after the submission of our manuscript. We benchmarked the latest DeepVariant v1.10 nevertheless: it yields worse performance (lower F1) than the pipeline we benchmarked against in all samples. We hypothesize that this is due to the presence of only one

pangenome-aware genotyping model which is not Giraffe-refined (Hickey *et al*, Nature Biotechnology, 2024, doi.org/10.1038/s41587-023-01793-w) as we have used in our benchmarks. We also verified that DeepVariant v1.10 is not compatible with the Giraffe-refined model used in our benchmarks. We therefore believe the Giraffe/DeepVariant pipeline used in our benchmarks represents the latest validated and published configuration compatible with the latest HPRC graph.

We also compared against the DRAGEN v4.2.4 pipeline, which was used by its authors in their recent publication (Behera *et al*, Nature Biotechnology, 2025, doi.org/10.1038/s41587-024-02382-1). This is the most recent major DRAGEN release and is more recent than the version currently used in the DRAGEN popgen pipeline (v3.7.8, developer.illumina.com/dragen/dragen-popgen, accessed May 2026). Several large-scale sequencing projects use this pipeline such as All of Us, the UK Biobank and NashBio. DRAGEN v4.2.4 therefore represents a published and validated version that is appropriate for this study.

In addition, continuously updating software versions during peer review would result in a moving benchmark and reduce reproducibility. We provide a stable comparison based on the most recently published and validated versions of each method.

Main manuscript text changes
Added in line 154: "We used the most recently published versions of each method."

- In Figure 4 and the supplement, the authors only show relative to the HG002 T2T benchmark in the low-confidence regions? This seems crazy - firstly, the high-confidence fraction of the assembly is the large majority (>90%) of the genome containing the large majority of variants, secondly, the variants in the low confidence fraction are, by design, much more suspect and more likely to be wrong. Figure 1 needs to be changed to show the HG002 benchmark high-confidence (and low and all-) benchmark results. Similarly, a complete set of results needs to be in the supplement. The fact that the current version is worse than the three year old version of Dragen on the high-confidence regions and tied with old versions of Giraffe is very concerning.

We thank you for pointing out that the HG002 benchmark may be seen as selectively presenting results as Figure 4F and Supplementary Figure 17 included only low-confidence regions. We have updated both figures as detailed above.

- The mapping “dark regions of GRCh38” is not a reasonable way of assessing accuracy because we do not know if the reads mapped in these regions are reliably mapped, and no attempt is made to check this. The simulated mapping results (which were created by the authors) show that the actual differences in performance of the mappers are relatively small. I think all the dark region analysis is showing is differences in the way the mappers give up in mapping to highly repetitive sequences according to minimizer repetition thresholds.

While we agree that benchmarking in dark regions is inherently limited by the lack of comprehensive ground truth, these regions represent genomic contexts where pangenome-based mapping is expected to provide the greatest benefit. We therefore view the dark region analyses as complementary to the other evaluations in the manuscript. While imperfect, it provides practical insight into mapper behavior in difficult genomic contexts.

We have added clarifications on why the comparison of dark regions is relevant for our study.

Main manuscript text changes
Added in lines 162-163: “These regions represent challenging genomic contexts in which the advantages of pangenome mapping are expected to be the most pronounced”

- The authors spend a lot of time comparing to Dragon and Giraffe using DV as their genotyping tool for Weaver, but once they shift to genotyping across the Icelandinc cohort they switch to their in house genotyper. This is justified as being necessary given the computational cost of DV, but if that is the case they MUST include weaver/graphtyper results in Figure 4 and the supplement. Presumably they don’t, because they know it will be weaker than Dragen et al.

Thank you for pointing this out, we had indeed not made explicit in the manuscript that GraphTyper is designed for population-scale joint genotyping rather than single sample calling. Graphtyper has been successfully used for genotyping population-scale cohorts such as in the Icelandic population (Eggertsson *et al*, Nature Genetics, 2017, https://doi.org/10.1038/ng.3964) and half a million individuals in the UK Biobank (The UK Biobank Whole-Genome Sequencing Consortium, Nature, 2025, https://doi.org/10.1038/s41586-025-09272-9). For this reason, DeepVariant was more appropriate for the single sample evaluations. We have now explicitly stated this in the revised manuscript.

Main manuscript text changes

Updated lines 209-215: "Compared to the single-sample evaluations for which DeepVariant was the most appropriate variant caller, GraphTyper is designed for the joint genotyping of population-scale cohorts and places less emphasis on single-sample calling performance. Furthermore, genotyping the cohort with DeepVariant was estimated to require an order of magnitude more computation time than GraphTyper, exceeding 10 million CPU hours, and therefore GraphTyper was used for this task, consistent with our previous cohort of Icelanders."

In terms of the second issue, overall marginal improvements, as the authors acknowledge the Emblask pipeline presented is for a sequencing technology that is now (long!) discontinued. I stressed this is my earlier reviews, but the advice to reduce this does not seem to have been taken. Further, all of the work done with the assemblies results in a very slightly improved set of small variants to feed to Weaver for the HPRC-ICE pangenome. Nothing continues to be done with the SVs. However, as Supplementary Figure 17 shows (again, for some reason only in the low-confidence regions - this is not justifiable, btw) the improvement in variant calling accuracy is negligible to non-existent with the addition of all the Icelandic data (btw, that result should be in Figure 4!). No attempt is made to show that the ICE portion improves variant ascertainment in the Icelandic population, or really anything else that would convince you that HPRC-ICE is better than HPRC. As such, half the paper CONTINUES to feel like mostly a waste of time, particularly given that the assemblies will never be released for privacy reasons.

We appreciate the reviewer's perspective and we have reduced the Emblask presentation (~10% fewer words) in the revised manuscript while retaining methodological details necessary to understand the downstream analyses.

Regarding "Nothing continues to be done with the SVs", we acknowledge we may not have sufficiently highlighted in our previous response that the HPRC structural variants are incorporated into the pangenome graph and analyzed in the manuscript (lines 256-260). We had provided in our previous response to the reviewers a justification to only include the HPRC structural variants but not the Icelandic structural variants in the graph as well as explaining it in the main manuscript text (lines 108-110).

We have updated Supplementary Figure 17 to show all regions (high- and low-confidence). While we agree that the improvements in per-sample calling are modest, at the population-scale these improvements resulted in a 6.17%

increase in the number of accurately called variants, among which were disease-associating variants missing from previous efforts.

Regarding the comment "No attempt is made to show that the ICE portion improves variant ascertainment in the Icelandic population, or really anything else that would convince you that HPRC-ICE is better than HPRC", the analysis comparing mapping to HPRC-ICE and HPRC is presented in the supplementary materials (Supplementary Figures 16-18) and discussed in lines 189-192 of the manuscript. Our results showed HPRC-ICE improved both variant calling sensitivity and precision compared to HPRC, both in Icelanders and HG002. It is expected that the improvements in the benchmark are modest as the variants unique to the ICE portion are rare variants and each sample only carries a small portion of these rare variants. Consequently, including them shows limited benefit at the level of individual sample genotyping. However, the main benefit of the ICE portion is at large-scale to identify rare disease-associating variants that are missing from HPRC. The examples of GBA/Parkinson's and CBS/Homocystinuria demonstrate the value of the ICE portion as they would not have been discovered without it (lines 291-293 and 314-315). We appreciate your comment and realize that the value of the ICE portion may not have been emphasized sufficiently. We have updated the manuscript (lines 353-355) to address that.

Main manuscript text changes

The text on Emblask has been reduced by ~10%.

Updated sentence in lines 353-355: "Both uncovered variants were detected only in Icelandic assemblies, emphasizing the importance of including rare variants in the pangenome and building pangenomes from sequenced individuals affected with genetic disorders".

Finally, in terms of data release and software- the authors should release the data and software instead of saying they will release it. The github link for Weaver is empty, and the code ocean link provided is for testing with a toy region.

We appreciate the feedback regarding software and data availability. The reviewer is correct that CodeOcean provides instructions for testing on a toy region as a demonstration on how to run the software. However, the software provided can also be run with any other dataset. We acknowledge that this point could have been made clearer. The public GitHub repository for Weaver (github.com/DecodeGenetics/weaver) contains the Weaver binary and usage instructions since March 2025:

https://github.com/DecodeGenetics/weaver/releases/tag/v0.2.0
https://github.com/DecodeGenetics/weaver/blob/e07b078a16b1b88ad2fc4eba54
57d95ee96d8d16/README.md

The binary can also be used with other datasets. In this revision, we have additionally added to that repository the Weaver source code, which was previously shared only with reviewers via CodeOcean. The Emblask code (github.com/DecodeGenetics/Emblask) has been publicly available since the initial submission.

As for data, we are sharing the HPRC-ICE pangenome reference files with reviewers.
HPRC-ICE is accessible via Zenodo using the following URL
https://zenodo.org/records/19609699?preview=1&token=eyJhbGciOiJIUzUxMiIsI
mlhdCI6MTc3NjQxNTgyNCwiZXhwIjoxODA3ODMzNTk5fQ.eyJpZCI6ImY4ZTljZj
MzLTczOTctNDA4My1iM2JkLTNlYTEyNDdmYWNjYyIsImRhdGEiOnt9LCJyYW5
kb20iOiI5YWJiNjIzYmZlZjVhOGRiN2U4YWFjM2JmODA2OWNmMCJ9.kaPHbqn
bDoplVMeUI5QcNEgsUy2oHuD-
hsMoOuW2kP8C7yncmWlKAzKF6P90cAnTA33x9EsxfQQfmIXlUJBfKQ

We are very thankful for all reviewers’ suggestions and comments, which we believe have strengthened our work and improved the readability and comprehension of the manuscript.

Referee #1 (Remarks to the Author):

The authors have addressed our previous concerns. For this review, we are focusing only on comments and revisions related to issues relayed by reviewer #3 in the last round. Overall, we are not concerned at all about the use of somewhat older sequencing technology and bioinformatics tools in this work. This is how time works, and just because something was done in the past does not mean it is not useful. We want to highlight again here: to us, the main point of this ms is the biological/clinical knowledge gain of better variant detection, not necessary massive methods advances.

Below, the previous comment from reviewer #3 is quoted, and our responses to this follow in a paragraph flagged with **.

“[The manuscript] is unfortunately selectively presenting somewhat marginal improvements, (2) it continues to focus on a now archaic (in terms of sequence technology) assembly pipeline that results in very small downstream improvements for genotyping, and (3) doesn’t actually make any of the data (even in derived, de-identified form) available.”

** Authors’ responses to this comment are appropriate. More specific comment responses are addressed below.

Thank you.

“The authors are using very old versions of software to compare against, particularly the Dragon software they use (the version is only given in the supplement!) is from July 2023. I am aware that subsequent versions have substantially improved performance since that time. Similarly, I know versions of the DeepVariant for Giraffe have been steadily improving. It may be a chore, but it is not reasonable to compare to these very old software at this point, and I think things would look quite different compared to the latest Dragen”

** In their rebuttal, the authors state “We have now explicitly stated in the manuscript that we chose the latest published versions for our benchmarks (line 154)”. This is true, however, it is usually required that the authors also include the actual versions/releases of the software they used. I assume this will be resolved during the checklist process, so no concern from us.

We have added versions number to the main text (lines 152-153).

“In Figure 4 and the supplement, the authors only show relative to the HG002 T2T benchmark in the low-confidence regions? This seems crazy - firstly, the high-confidence fraction of the assembly is the large majority (>90%) of the genome containing the large majority of variants, secondly, the variants in the low confidence fraction are, by design, much more suspect and more likely to be wrong. Figure 1 needs to be changed to show the HG002 benchmark high-confidence (and low and all-) benchmark results. Similarly, a complete set of results needs to be in the supplement. The fact that the current version is worse than the three year old version of Dragen on the high-confidence regions and tied with old versions of Giraffe is very concerning.”

** The authors address this comment by adjusting Fig. 4 and some associated text. While we don’t fully agree with reviewer #3’s reason or tone, there is a bit of a point - even though the vast majority of pangenome improvements should be in hard-to-call regions, the effects of bad variant calling in challenging parts of the genome can sometimes bleed over into the single-copy regions. So, it’s probably a good idea to show whole-genome statistics. That said, we were also fine with the previous presentation. Regardless, we feel like the changes to the text and the responses to reviewer #3 in their first comment are fine and no additional adjustments need to be made.

Thank you.

“The mapping “dark regions of GRCh38” is not a reasonable way of assessing accuracy because we do not know if the reads mapped in these regions are reliably mapped, and no attempt is made to check this. The simulated mapping results (which were created by the authors) show that the actual differences in performance of the mappers are relatively small. I think all the dark region analysis is showing is differences in the way the mappers give up in mapping to highly repetitive sequences according to minimizer repetition thresholds.”

** Author responses to this are fine. The addition of that one sentence doesn’t add much but does address this comment to some extent.

Thank you.

“The authors spend a lot of time comparing to Dragon and Giraffe using DV as their genotyping tool for Weaver, but once they shift to genotyping across the Icelandinc cohort they switch to their in house genotyper. This is justified as being necessary given the computational cost of DV, but if that is the case they MUST include

weaver/graphtyper results in Figure 4 and the supplement. Presumably they don't, because they know it will be weaker than Dragen et al."

** The authors have added some clarification to the text related to this comment. These changes are appropriate and help with communication.

Thank you.

"In terms of the second issue, overall marginal improvements, as the authors acknowledge the Emblask pipeline presented is for a sequencing technology that is now (long!) discontinued. I stressed this is my earlier reviews, but the advice to reduce this does not seem to have been taken. Further, all of the work done with the assemblies results in a very slightly improved set of small variants to feed to Weaver for the HPRC-ICE pangenome. Nothing continues to be done with the SVs. However, as Supplementary Figure 17 shows (again, for some reason only in the low-confidence regions - this is not justifiable, btw) the improvement in variant calling accuracy is negligible to non-existent with the addition of all the Icelandic data (btw, that result should be in Figure 4!). No attempt is made to show that the ICE portion improves variant ascertainment in the Icelandic population, or really anything else that would convince you that HPRC-ICE is better than HPRC. As such, half the paper CONTINUES to feel like mostly a waste of time, particularly given that the assemblies will never be released for privacy reasons."

** The modest changes to the text are in line with how we would have approached this comment. There really isn't much to be done about old technology, and it's a bit of a bummer this reviewer latched on to that. The data is there and still needs to be analyzed. As for the SVs, we think the treatment for those and the SI fig are good additions.

Thank you.

"Finally, in terms of data release and software- the authors should release the data and software instead of saying they will release it. The github link for Weaver is empty, and the code ocean link provided is for testing with a toy region."

** Response here is good, but please ensure this information makes it into the final manuscript and not just the rebuttal.

All data and code have now been made publicly available with all relevant links added to the manuscript.

Referee #2 (Remarks to the Author):

I co-reviewed this manuscript with one of the reviewers who provided the listed reports.

Thank you.

Referee #2 (Remarks on code availability):

Code for all new programs is publicly available, as are data from the manuscript.

Thank you.

We would like to thank all the reviewers for taking the time to evaluate our manuscript thoroughly and for providing insightful comments that have significantly improved it.
